# Evolutionary mode and timing of dissemination of high-grade serous carcinomas

**DOI:** 10.1172/jci.insight.170423

**Published:** 2024-01-04

**Authors:** Anita Sveen, Bjarne Johannessen, Solveig M.K. Klokkerud, Sigrid M. Kraggerud, Leonardo A. Meza-Zepeda, Merete Bjørnslett, Katharina Bischof, Ola Myklebost, Kjetil Taskén, Rolf I. Skotheim, Anne Dørum, Ben Davidson, Ragnhild A. Lothe

**Affiliations:** 1 Department of Molecular Oncology, Institute for Cancer Research, Oslo University Hospital, Oslo, Norway.; 2 Institute of Clinical Medicine, Faculty of Medicine, University of Oslo, Oslo, Norway.; 3 Department of Tumor Biology, Institute for Cancer Research,; 4 Genomics Core Facility, Department of Core Facilities, Institute for Cancer Research,; 5 Department of Gynecological Oncology, The Norwegian Radium Hospital, and; 6 Department of Cancer Immunology, Institute for Cancer Research, Oslo University Hospital, Oslo, Norway.; 7 Department of Clinical Science, University of Bergen, Bergen, Norway.; 8 Department of Informatics, University of Oslo, Oslo, Norway.; 9 Department of Pathology, Oslo University Hospital, Oslo, Norway.

**Keywords:** Genetics, Oncology, Bioinformatics, Clonal selection, Molecular biology

## Abstract

Dissemination within the peritoneal cavity is a main determinant of poor patient outcomes from high-grade serous carcinomas (HGSCs). The dissemination process is poorly understood from a cancer evolutionary perspective. We reconstructed the evolutionary trajectories across a median of 5 tumor sites and regions from each of 23 patients based on deep whole-exome sequencing. Polyclonal cancer origin was detected in 1 patient. Ovarian tumors had more complex subclonal architectures than other intraperitoneal tumors in each patient, which indicated that tumors developed earlier in the ovaries. Three common modes of dissemination were identified, including monoclonal or polyclonal dissemination of monophyletic (linear) or polyphyletic (branched) subclones. Mutation profiles of initial or disseminated clones varied greatly among cancers, but recurrent mutations were found in 7 cancer-critical genes, including *TP53*, *BRCA1*, *BRCA2*, and *DNMT3A*, and in the PI3K/AKT1 pathway. Disseminated clones developed late in the evolutionary trajectory models of most cancers, in particular in cancers with DNA damage repair deficiency. Polyclonal dissemination was predicted to occur predominantly as a single and rapid wave, but chemotherapy exposure was associated with higher genomic diversity of disseminated clones. In conclusion, we described three common evolutionary dissemination modes across HGSCs and proposed factors associated with dissemination diversity.

## Abstract 2

<p>Clonal evolution modeling shows that most high-grade serous carcinomas have polyclonal cancer dissemination that occurs&nbsp;as a single and rapid wave late in their evolutionary trajectories.</p>

## Introduction

High-grade serous carcinomas (HGSC) have a unique pattern of tumor development and cancer dissemination. The cancers originate in the serous epithelial cell layer of the distal fallopian tube in approximately 80% of the cases (1, 2) and more rarely in the ovaries or the peritoneum. HGSCs have the propensity for early and direct seeding of exfoliated tumor cells via the ascites in the peritoneal cavity (3). Patients are therefore commonly diagnosed with disseminated disease and locoregional involvement of the ovaries, other intraperitoneal organs, and the omentum. Approximately two-thirds of the cancers are stage III or IV at diagnosis (1), and these patients have a median overall survival of only approximately 40 months (4).

*TP53* mutation is an early event in almost all HGSCs (5–7). The “p53 signature” of abnormal TP53 immunostaining in the morphologically normal tubal epithelium is considered the earliest precancer lesion (6). *TP53* aberrations contribute to a permissive state, with frequent DNA damage (8), development of high genomic complexity, and simultaneous coevolution of multiple mutational processes (9). Homologous recombination deficiency (HRD) occurs in approximately half of the cancers (10); it is caused by germline mutations of *BRCA1* or *BRCA2* in at least 15% (1). Cancers with HRD are particularly sensitive to platinum-based chemotherapy and PARP inhibition, providing an opportunity for molecularly guided treatment (11, 12). However, most (80%) of the cancers progress after initial response to such treatment, and molecular tumor heterogeneity is a major contributing factor to treatment failure (13–15).

Molecular reconstruction of cancer evolution has supported the notion of a highly diverse dissemination pattern of HGSCs. Most possible routes to cancer dissemination have been described, including monoclonal and polyclonal seeding as well as both unidirectional seeding and reseeding among tumor sites in the peritoneum (16, 17). Both the clonal diversity of the cancers and the dissemination process are likely shaped by the tumor microenvironment, and there is a propensity for dissemination to the adipose tissue of the omentum (18, 19). However, most phylogenetic studies have been based on a small number of patients (6 to 15) (5, 16, 17, 20–25), and this has precluded conclusions regarding the general patterns of clonal dissemination among HGSCs. In view of the prognostic impact, there is a need to better understand the molecular determinants and patterns of cancer dissemination. In this study, we modeled the clonal evolution of dissemination across multiple tumors and tumor regions from each of 23 patients with HGSC.

## Results

### Somatic mutation profiles of HGSCs are shaped by DNA damage repair deficiency.

**Figure 1:**
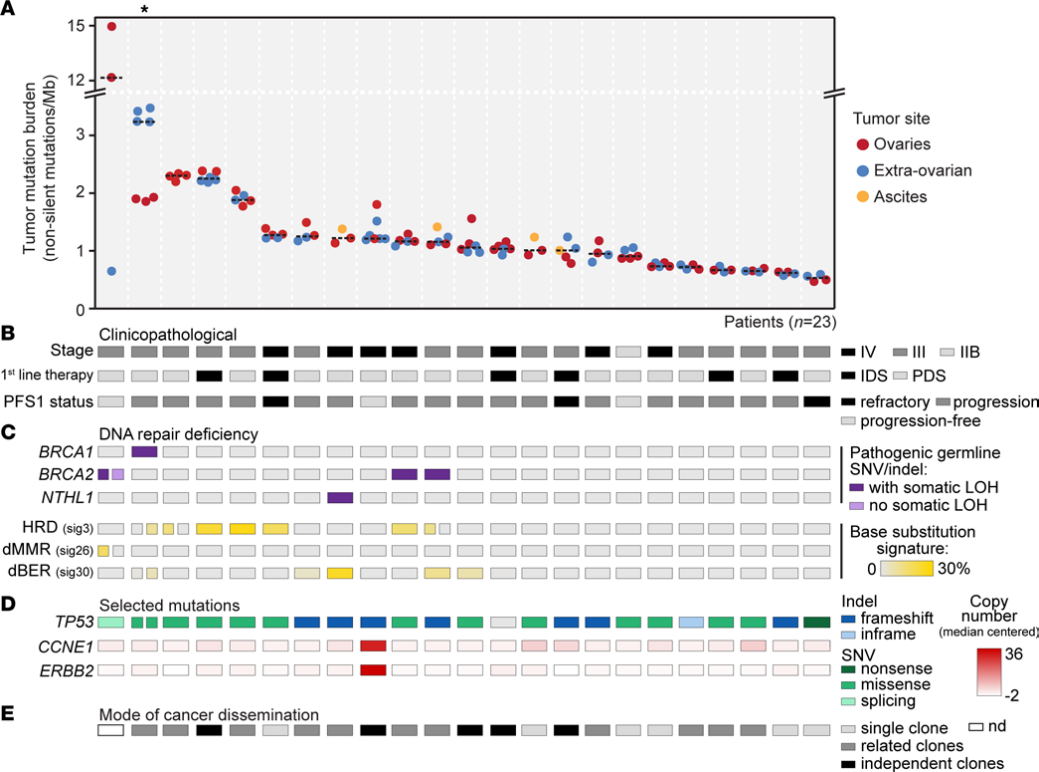
Mutation profiles across multiple tumors and tumor regions from disseminated high-grade serous carcinomas. (**A**) Mutation burden from whole-exome sequencing of 108 tumor or ascites samples from 23 patients with disseminated high-grade serous carcinoma. Samples are grouped by patient and colored according to tumor site. The black dashed lines indicate the median mutation burden per patient, and patients are ranked in order of a decreasing median mutation burden. The asterisk marks a patient with polyclonal cancer origin. (**B**–**D**) Selected (**B**) clinicopathological and (**C** and **D**) molecular characteristics per patient. Color labels and scales are defined to the right of each parameter. The proportion of each base substitution signature represents the median per patient. Split boxes indicate intrapatient heterogeneity between the ovarian (left) and extraovarian (right) samples. Remaining molecular characteristics were homogeneous in all samples from each patient. (**E**) Three modes of cancer dissemination were determined by clonality modeling, as illustrated in Figure 3. dBER, deficient base excision repair; dMMR, deficient mismatch repair; IDS, interval debulking surgery (with neoadjuvant chemotherapy); LOH, loss of heterozygosity; Mb, megabase; nd, not determined; PDS, primary debulking surgery (with adjuvant chemotherapy); PFS1, progression-free survival after first-line therapy; sig, signature.

A median of 5 multisite or multiregional tumor samples from each of 23 patients with disseminated HGSC (Supplemental Table 1; supplemental material available online with this article; https://doi.org/10.1172/jci.insight.170423DS1; total *n* = 108 cancer samples; Supplemental Figures 1–3) were analyzed by deep whole-exome sequencing (mean depth of coverage 668×; Supplemental Table 2). The median tumor mutation burden (TMB) per sample was 1.1 nonsilent mutations per megabase (10–90 percentile range 0.6–2.3 mutations). The TMB was not associated with the tumor purity, tumor site (ovarian, extraovarian, or ascites), the depth of sequencing coverage, previous exposure to chemotherapy (Supplemental Figure 4), patient age (Spearman’s rank correlation = –0.1, *P* = 0.8), or cancer stage at diagnosis (*P* = 0.8 from Wilcoxon’s test of FIGO stage II or III versus IV). There was little intrapatient heterogeneity of the TMB, with the notable exceptions of the 2 patients with the highest median TMB (Figure 1 and Supplemental Figure 5).

The TMB was highest in patients with mutation signatures of deficient DNA damage repair. HRD scoring, based on the Catalogue of Somatic Mutations in Cancer (COSMIC) base substitution signature 3 (26), is proposed to have superior performance in predicting survival benefit from PARP inhibition compared with HRD genomic instability scores and germline mutations of *BRCA1* and *BRCA2* (27). Signature 3–associated HRD was identified in 7 (30%) of the patients, 3 (43%) of whom had pathogenic germline mutations and concomitant somatic loss of heterozygosity of *BRCA1* or *BRCA2* (Figure 1). Deficient DNA mismatch repair (COSMIC signature 26) was found in the ovarian tumor samples from the patient with the highest median TMB but not in the extraovarian sample. This was consistent with the hypermutation phenotype (>12 mutations per megabase) and with results from PCR-based microsatellite instability (MSI) testing, confirming intrapatient heterogeneity of MSI. The patient had a pathogenic germline mutation of *BRCA2* but not of genes involved in DNA mismatch repair. Notably, there was no loss of heterozygosity at the *BRCA2* locus in the DNA damage repair–proficient extraovarian sample. Strong base excision repair deficiency (COSMIC signature 30) was found in all samples from a patient with a nonsense germline mutation and somatic loss of heterozygosity of *NTHL1* (*NTHL1*^Q90*^), indicating an NTHL1-associated polyposis syndrome (28). Patients with any of the 3 types of deficient DNA damage repair (*n* = 9, 39%) had a higher median TMB than the remaining and “triple-proficient” patients (*P* = 5 × 10^–5^ by Wilcoxon’s test). The difference in TMB was similar when including all cases with weak signals for COSMIC signature 30 (two additional patients) in the DNA damage repair–deficient group (*P* = 1 × 10^–5^). One triple-proficient patient with an intermediate TMB had amplifications of *CCNE1* and *ERBB2* in all tumor samples (>20 additional copies; Figure 1), supporting oncogene amplification as a driving mechanism in the absence of DNA damage repair deficiency (29, 30).

Principal components analysis of samples based on their mutation profiles (the relative contribution of each COSMIC base substitution signature) showed that principal component 1 was most strongly correlated to the signatures of HRD and base excision repair (positively and negatively, respectively; Supplemental Figure 6A). This supported that deficient DNA damage repair was a prominent driver of mutation diversity across the tumors. Tumor samples from each patient clustered together in the principal components analysis, suggesting lower intrapatient than interpatient mutation heterogeneity (with the exception of the 2 patients with the highest median TMB; Supplemental Figure 6B).

### Clonal diversity of mutations and mutation processes.

All patients except 1 (96%) had *TP53*-mutated tumors. The *TP53* wild-type cancer had a somatic *BRCA1* mutation, and the HGSC diagnosis was confirmed by histopathology (Supplemental Figure 2B). Only 1 patient had *TP53* mutation heterogeneity. Different *TP53* missense mutations were found in the ovarian and extraovarian tumors from the patient with the second highest median TMB, suggesting a polyclonal cancer origin (Figure 1). DNA copy number profiles across tumors and patients were similar to previously reported data for ovarian cancer (31) (Supplemental Figure 7A), and the level of genomic complexity was high (median proportion of genes affected by copy number aberrations per sample, 61%; 10–90 percentile range, 39%–71%). The lowest copy number aberration burden was found in the single patient wild-type for *TP53* (Supplemental Figure 7B).

**Figure 2:**
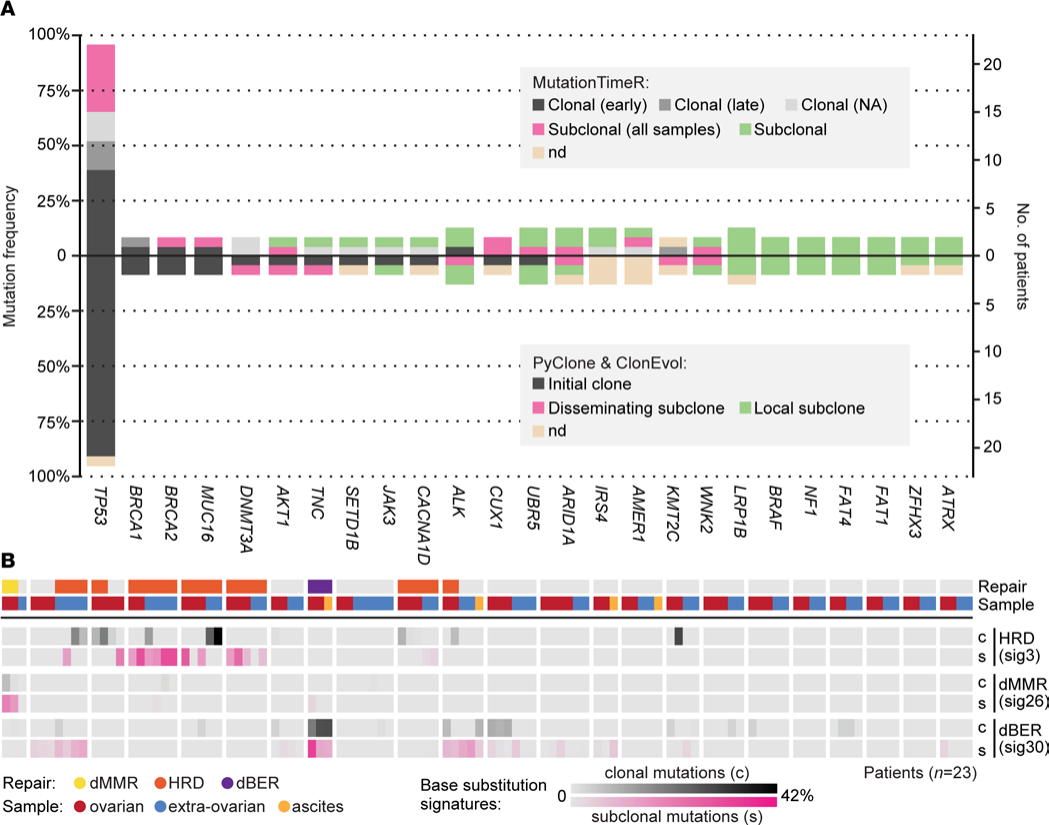
Clonal designations of recurrent mutations and mutation signatures. (**A**) The vertical axes show the mutation frequency (calculated patient-wise among 23 patients) of all oncogenes and tumor suppressor genes (defined by the Cancer Gene Census) with recurrent nonsilent SNVs or indels across the cancers. Mutations are colored according to designations of clonality based on MutationTimeR (top part) or PyClone and ClonEvol (bottom part; reverse vertical axes orientation). Mutations were considered clonal according to MutationTimeR if designated as such in at least 1 sample per patient, and subclonal mutations were divided according to their homogeneous (pink) or heterogeneous (green) presence across samples per patient. Results from PyClone and ClonEvol are from clonality modeling across all samples per patient. Two polyclonal cancers in 1 patient were analyzed separately and summarized patient-wise. (**B**) The sample-wise proportions of 3 selected base substitution signatures of deficient DNA damage repair, calculated separately for clonal (black) and subclonal (pink) mutations, as designated by MutationTimeR. Results are presented per patient (separated by white spaces) in the same order as in Figure 1 (ranked according to a decreasing median tumor mutation burden) and per sample ordered by tumor site. The two top rows indicate the type of DNA damage repair deficiency detected in each patient (Repair) and the tumor site of each sample (Sample). c, clonal; dBER, deficient base excision repair; dMMR, deficient mismatch repair; nd, not determined; s, subclonal.

No oncogenes or tumor suppressor genes (defined in the Cancer Gene Census [CGC]) beyond *TP53* had frequent nonsilent single nucleotide variants (SNVs) or insertions and deletions (indels) across the cancers (Figure 2A; the most frequent mutations in general are shown in Supplemental Figure 8). MutationTimeR (32) was used to categorize the mutations as clonal (early, late, NA) or subclonal in each sample. *TP53* mutations were identified as clonal in at least one sample in the majority of patients (65%). Somatic *BRCA1* and *DNMT3A* mutations were also identified as recurrently clonal. The remaining recurrently mutated genes were either diversely categorized or identified as subclonal in all patients.

In contrast to the total TMB, the TMB of clonal mutations varied substantially across samples from each patient, indicating intrapatient heterogeneity at the subclonal level (Supplemental Figure 9). The clonal TMB did not vary according to tumor site (ovarian versus extraovarian; *P* = 0.5 by paired *t* test) or previous chemotherapy exposure (*P* = 0.2 by Wilcoxon’s test of the median clonal TMB per patient). Reestimation of the COSMIC base substitution signatures for clonal and subclonal mutations separately showed that the HRD signature was detectable among clonal mutations in 6 of the 7 affected patients, including patients with germline mutations and loss of heterozygosity of *BRCA1* or *BRCA2* (Figure 2B). However, HRD was never clonal in all samples from a patient, suggesting intratumor heterogeneity and evolution of HRD during progression. There was no apparent propensity for clonal involvement of ovarian tumors in particular. Five of the patients also had subclonal HRD in some samples, suggesting that genomic instability was maintained during dissemination. Exclusively subclonal HRD was found in 1 patient wild-type for *BRCA1* and *BRCA2*, possibly reflecting late development of HRD. The signature of base excision repair deficiency was clonal in all samples from the patient with a germline *NTHL1* mutation, and the phenotype was maintained among subclonal mutations, strongly supporting its involvement in the development and progression of this cancer. In contrast, DNA mismatch repair deficiency was predominantly subclonal in the MSI positive ovarian tumor.

### Diverse and polyclonal dissemination of most HGSCs.

**Figure 3:**
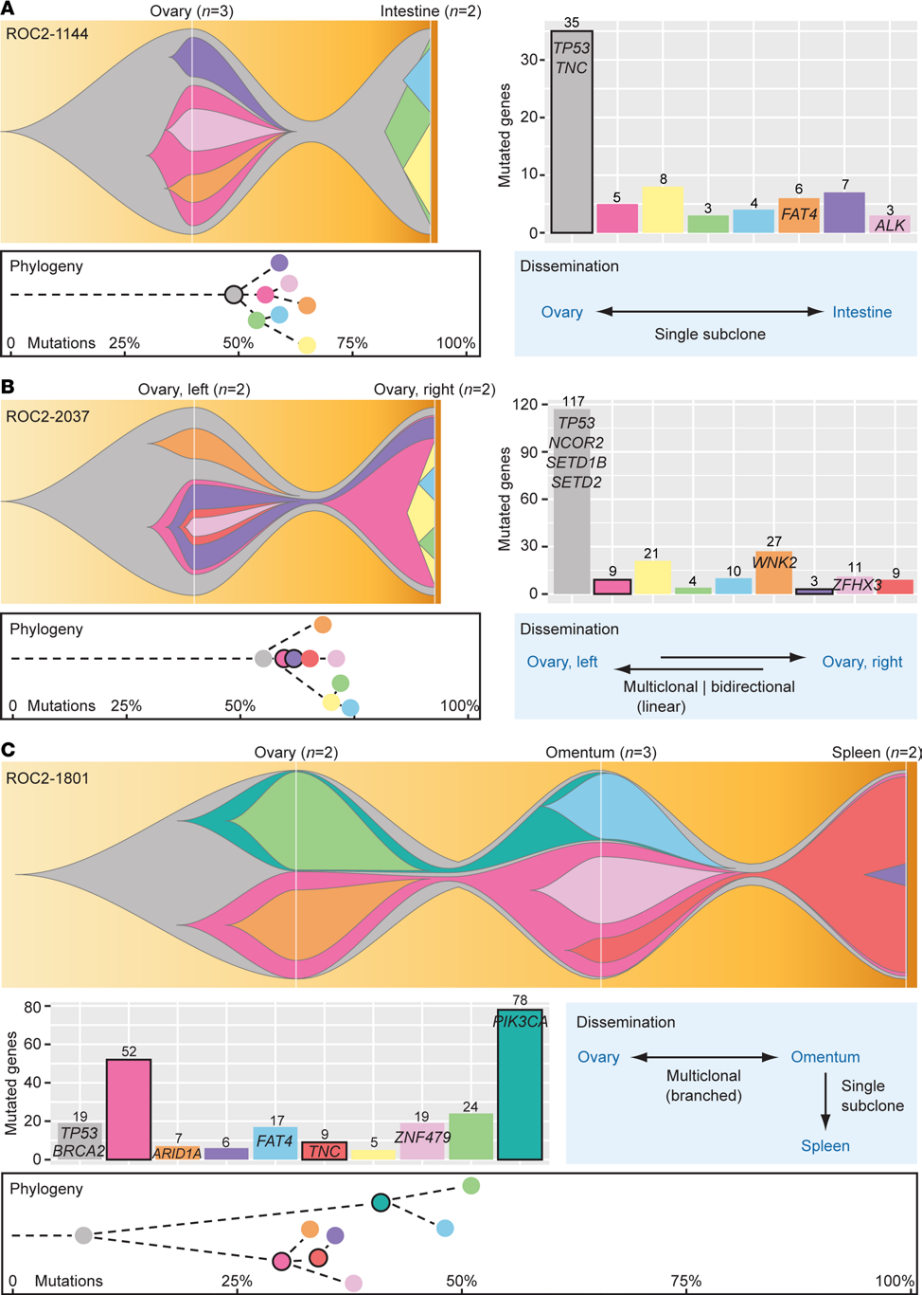
Three common modes of cancer dissemination. The 3 categories of cancer dissemination are illustrated by example cases, including (**A**) dissemination of a single clone, (**B**) polyclonal dissemination of monophyletic subclones of a linear evolutionary lineage (related subclones), and (**C**) polyclonal dissemination of polyphyletic subclones of a branched evolutionary lineage (independent subclones). Fish plots illustrate the subclonal architecture of each cancer, with the estimated cellular prevalence of each subclone at each tumor site indicated along the vertical axis (white lines). Bar plots show the number of mutations (nonsilent and silent) per subclone, and nonsilent mutations in oncogenes and tumor suppressor genes (defined by the Cancer Gene Census) are indicated. White boxes with black outlines illustrate the phylogeny of each cancer, with dots representing subclones, dashed lines indicating a linear evolutionary lineage, and the horizontal axis representing the relative mutation time for development of each clone, plotted as the proportion of mutations in each clone relative to the total number of mutations in the cancer model. Charts with arrows illustrate the direction of seeding between tumor sites, with two parallel arrows indicating bidirectional seeding and a bidirectional arrow indicating that the direction is undetermined. Subclones have consistent colors in all plots per cancer. Disseminated subclones are marked by black outlines in the bar plots and phylogenetic representations. None of the illustrated cancers were exposed to neoadjuvant chemotherapy or refractory to first-line treatment. The cancer in **B** had bidirectional seeding of the pink subclone from the left to the right ovary, followed by the purple subclone in the opposite direction. There were several possible models for the relationship of the red, yellow, and orange subclones in this cancer, but this had no effect on the designated mode of dissemination.

The subclonal tumor architecture and dissemination of each HGSC was further modeled across samples per patient using PyClone (33) and ClonEvol (34). The hypermutated HGSC could not be accurately modeled due to a large number of mutation clusters, but the hypermutated ovarian tumor contained all mutations and mutation clusters of the nonhypermutated peritoneal tumor, consistent with a monoclonal cancer origin and supporting late and subclonal development of MSI (Supplemental Figure 10). The estimated cellular prevalence of each predicted clone in each modeled cancer is illustrated in Supplemental Figures 11–13. The fitted clonal architectures are illustrated with fish plots for selected example cancers in Figure 3 (corresponding mutation lists in Supplemental Table 3) and for other cancers in Supplemental Figures 14–20. LICHeE was evaluated as a second computational method for subclone predictions and phylogenetic inferences (35). There was strong proportionality in the number of predicted subclones per cancer according to the two methods (Spearman’s rank correlation = 0.8, *P* = 5 × 10^-6^; Supplemental Figure 21) and good overall correspondence of mutation clusters and predicted phylogenetic lineages (Supplemental Figures 22–24). Discordances were primarily due to merging of mutation clusters by LICHeE, consistent with the known proclivity of PyClone to predict a large number of subclones (36), or mutation filtering due to incompatibility in the modeling process. In discordant cases, the models predicted by LICHeE had poorer compatibility with cellular prevalence estimates (Supplemental Figure 11–13), and the models obtained by PyClone and ClonEvol were used for further analyses.

All cancers except 1 (96%) had a monoclonal origin and at least 1 common clone among all tumor sites and samples (Table 1). The patient with the second highest median TMB had no common mutations between the ovarian and extraovarian tumors, confirming a polyclonal cancer origin. There was dissemination between the 2 extraovarian tumor sites in this patient (Supplemental Figure 14). All cancers except 1 also showed a branched evolutionary pattern, with at least 2 unrelated and independently evolved subclones present in each tumor or across tumor sites. The single HGSC without branched evolution was exposed to neoadjuvant chemotherapy, and the model estimates were uncertain (ROC2-0812; Supplemental Figure 11 and Supplemental Figure 15B). LICHeE also predicted a linear phylogenetic lineage of this cancer (Supplemental Figure 22).

The median number of predicted clones was 5.5 per cancer (range, 3–9; Supplemental Figure 21), which is similar to results from a recent study based on whole-genome sequencing of end-stage HGSCs exposed to multiple lines of chemotherapy (25). The number of clones did not vary according to chemotherapy exposure (*P* = 0.7) or refractoriness to first-line treatment (*P* = 0.6, both by Wilcoxon’s test). There was a tendency toward a larger number of clones in cancers with low stromal infiltration scores of the ovarian tumor (evaluated by RNA sequencing and the ESTIMATE gene expression signature; ref. 37), but this was not found among chemonaive cancers separately (Supplemental Figure 25). A median of 35% of clones per cancer disseminated between at least 2 tumor sites, and the number of disseminated clones was not associated with the total number of clones in the cancer (*P* = 0.6 by Kruskal-Wallis test). The relatively wide 95% CI of the median (22%–40%) suggested diversity in the dissemination process, and 3 modes of dissemination were proposed. These included dissemination of a single clone and dissemination of multiple related or unrelated subclones of a linear or branched evolutionary lineage, respectively (Table 1 and example plots in Figure 3).

Six HGSCs (27%) had monoclonal dissemination (first category; Figure 3A and Supplemental Figure 11, Supplemental Figure 15, and Supplemental Figure 22). Dissemination occurred prior to subclonal development in 4 of these cases, with the initial clone as the disseminated clone and with independent branched evolution at the separate tumor sites after dissemination. The direction of seeding of the initial clone was not possible to determine, but diagnostic histopathology indicated a fused fallopian tube and ovary in 1 case, suggesting tubal origin and seeding from the ovary to the intestine (Figure 3A). In the 2 cancers with monoclonal dissemination after subclonal development, seeding appeared to occur from the extraovarian tumor (omentum) to the ovary (Supplemental Figure 15B; 1 case had a known cancer origin in the fallopian tube).

The majority of HGSCs (73%) had polyclonal dissemination, most commonly of 2 or 3 subclones (*n* = 10 and 5 HGSCs, respectively), but there was also 1 example with 5 disseminated subclones. Polyclonal dissemination occurred predominantly with subclones of a linear evolutionary lineage (second category, monophyletic polyclonal seeding; *n* = 11 HGSCs; Figure 3B and Supplemental Figure 12, Supplemental Figure 14, Supplemental Figure 16, and Supplemental Figure 23). The direction of seeding was mostly undetermined in these cases, with the exception of 2 cancers with bidirectional seeding either between bilateral ovarian tumors (Figure 3B) or between a uterine and an ovarian tumor (Supplemental Figure 16B; known cancer origin in the fallopian tube) and 1 cancer with dissemination from the ovary to the omentum and ascites (Supplemental Figure 16C). The final 5 HGSCs had disseminated subclones of a branched evolutionary lineage (third category, polyphyletic seeding), with a discernible direction of seeding between at least 2 tumor sites in 4 of the cancers (Figure 3C and Supplemental Figure 13, Supplemental Figures 17–20, and Supplemental Figure 24). The direction of seeding was determined in altogether 9 HGSCs (41%; Table 1). There was no difference in the frequency of seeding to or from ovarian versus extraovarian tumors. However, ovarian tumors had a more complex subclonal architecture than extraovarian tumors, suggesting earlier tumor development in the ovaries. This was observed as a larger number of unique subclones (*P* = 0.04 by paired *t* test) and a tendency for more frequent branched evolution, also within the subset of chemonaive cancers (Supplemental Figure 26).

HGSCs with the least complex dissemination pattern (first category, monoclonal dissemination) had a lower patient-wise median TMB than HGSCs with polyclonal dissemination; this was also true within the subset of chemonaive cancers (*P* = 0.04 from Wilcoxon’s test; Figure 1). Among cancers with polyclonal dissemination, the number of disseminated clones was not associated with previous chemotherapy exposure (*P* = 0.5 by Wilcoxon’s test), but polyphyletic seeding was more common among treated cancers (3 of the 4 cancers exposed to chemotherapy 2–3 months before surgery had polyphyletic seeding; odds ratio, 11.8; *P* = 0.06 by Fisher’s exact test relative to chemonaive cancers). There was an increase in the stromal infiltration scores of ovarian tumors according to the complexity of cancer dissemination, observed as a nonsignificant trend from the first to the third dissemination category (Supplemental Figure 25). All HGSCs with the most complex dissemination pattern (third category, polyphyletic seeding) had more than 2 sampled tumor sites (*P* = 2 × 10^–4^ from Fisher’s exact test of enrichment) and were analyzed by a larger number of samples (*P* = 0.005 from Wilcoxon’s test). There was no association between the mode of dissemination and the site of the extraovarian tumor or any of the clinicopathological parameters listed in Supplemental Table 1.

### Recurrent driver mutations based on clonal composition.

Mutations were grouped according to their presence in the initial clone, a disseminated subclone, or a local subclone in the patient-wise models. The estimated number of mutations in the initial clone correlated with the median patient-wise TMB of clonal mutations summarized from the MutationTimeR sample-wise calls, showing correspondence of the 2 orthogonal modeling approaches (Spearman’s rank correlation = 0.56, *P* = 0.006; Supplemental Figure 9D). The recurrently mutated oncogenes and tumor suppressor genes also showed a good correspondence of their clonal designations (Figure 2). The largest discordance was found for mutations designated as subclonal in all samples per patient by MutationTimeR, while considered to belong to the initial clone in the patient-wise models.

All nonsilent mutations in *TP53*, *BRCA1*, *BRCA2*, and *MUC16* were estimated to belong to initial clones. Three additional oncogenes or tumor suppressor genes were recurrently mutated in either an initial or a disseminated clone. *AKT1* had a frameshift deletion of 11 base pairs in an initial clone (Supplemental Figure 14) and the oncogenic variant *AKT1*^E17K^ in a disseminated clone (Supplemental Figure 17) of 2 triple-proficient cancers. *PIK3CA*^E545Q^ represents another activating mutation of the PI3K/AKT1 pathway, and it was found in the disseminated clone of a third triple-proficient cancer (Figure 3C). *DNMT3A* had a missense mutation predicted to be damaging to protein function in the initial clone of a triple-proficient and chemonaive cancer (*DNMT3A*^R488Q^, PolyPhen-2 score, 0.48; Supplemental Figure 16A) as well as a frameshift deletion of 37 base pairs in a disseminated clone of a cancer with HRD exposed to neoadjuvant chemotherapy (Supplemental Figure 20). *TNC* had missense mutations in either the initial clone or a disseminated clone of triple-proficient and chemonaive cancers (Figure 3, A and C), but only the clonal mutation was predicted to be damaging (*TNC*^P1790L^, PolyPhen-2 score, 0.99; *TNC*^R1637C^, PolyPhen-2 score, 0.38).

### Diversity in the evolutionary timing of dissemination.

**Figure 4:**
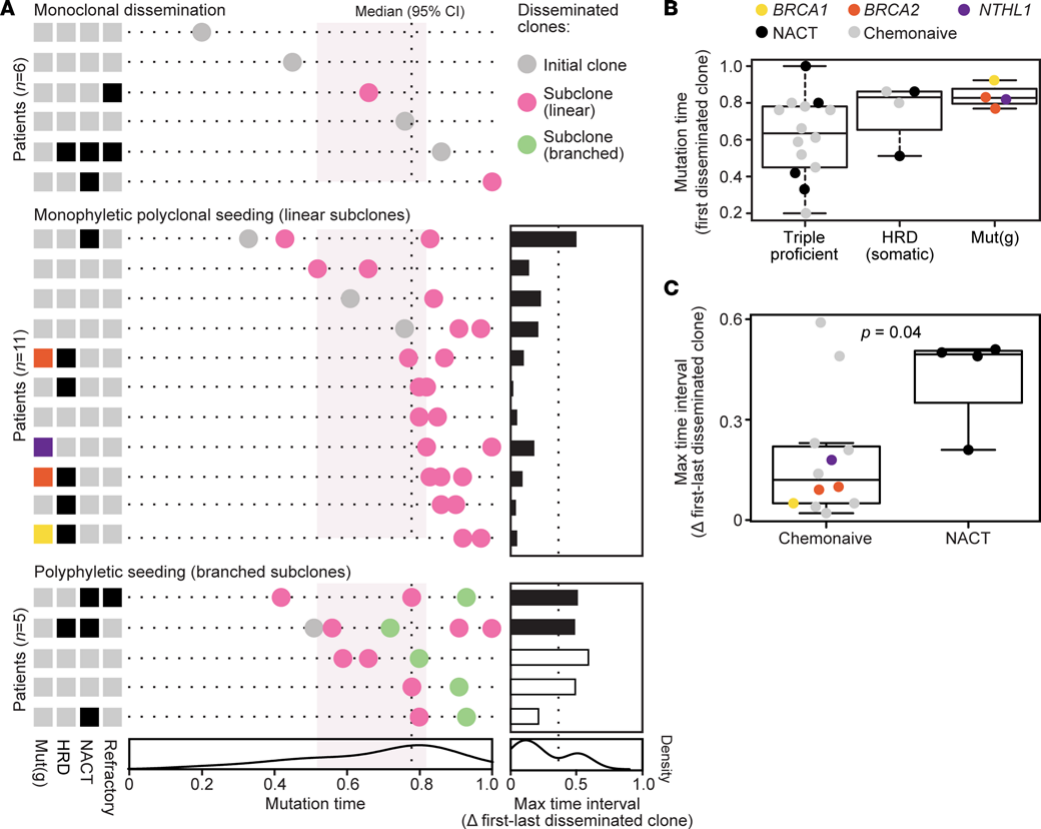
Diversity in the evolutionary timing of dissemination. (**A**) Each horizontal line represents a patient (*n* = 22; categorized according to the 3 modes of dissemination), and each dot represents a disseminated subclone (local/nondisseminated clones are not plotted). Pink and green indicate monophyletic and polyphyletic clones, respectively. Boxes to the left indicate germline mutations of DNA repair genes [Mut(g); color code shown in **B**], positivity for COSMIC base substitution signature 3 (HRD; black = “yes”), exposure to chemotherapy prior to sampling (neoadjuvant chemotherapy [NACT]), and refractory disease after first-line treatment. The horizontal axis shows the relative evolutionary timing of development of disseminated subclones as the mutation time on a scale from 0 to 1, representing the proportion of mutations per subclone relative to the latest developed subclone per cancer. Vertical dashed line and pink-shaded background represent the median mutation time and 95% CI of the first/only disseminated subclone across the cancers. Horizontal bar plots show the maximum mutation time between disseminated clones in each patient (black, time between the first and last disseminated clones of a monophyletic origin; white, time interval estimated as the total number of unique mutations in 2 polyphyletic clones, relative to the total number of modeled mutations in the cancer). (**B**) Box plot of the relative timing of development of the first disseminated clone according to DNA damage repair deficiency status. (**C**) Box plot of the time interval of dissemination (mutation time between the first and last disseminated clone in cancers with polyclonal dissemination) according to chemotherapy exposure (*P* value from Wilcoxon’s test). Bounds of boxes represent the interquartile range, lines within boxes represent the median, and whiskers represent 1.5× the interquartile range above the 75th percentile or below the 25th percentile.

The relative timing of dissemination along the evolutionary trajectory model of each HGSC was evaluated in mutation time (on a scale from 0 to 1), estimated as the proportion of mutations in the disseminated subclone(s) relative to the latest developed subclone per cancer (the subclone with the largest number of mutations). Disseminated clones developed relatively late in the evolutionary models of most cancers, and the median mutation time of the first or only disseminated clone was 0.77 (Figure 4A). However, there was considerable variation among the cancers, and the 95% CI of the median ranged from 0.52 to 0.82. The timing of dissemination was not associated with chemotherapy exposure (*P* = 0.9 by Wilcoxon’s test) but was correlated to the median patient-wise TMB, most strongly among chemonaive patients (Spearman’s rank correlation = 0.7, *P* = 0.003).

Patients with germline mutations of DNA damage repair genes (*BRCA1*, *BRCA2*, or *NTHL1*) had a later evolutionary onset of dissemination than patients with triple-proficient cancers (*P* = 0.03 by Wilcoxon’s test; Figure 4B). There was no difference between patients with germline mutations and somatic development of HRD. Results were similar when analyzing chemonaive cancers only, showing a later onset in DNA damage repair–deficient cancers (germline or somatic) than in triple-proficient cancers (*P* = 0.003 by Wilcoxon’s test). Two additional cancers with weak somatic signals for deficient base excision repair (both chemonaive; Figure 1) also had late onset of dissemination, and inclusion of these in the DNA damage repair–deficient group strengthened the difference relative to triple-proficient cancers (*P* = 9 × 10^–4^ by Wilcoxon’s test). There was no association between the onset of dissemination and patient age (Spearman’s rank correlation = –0.3, *P* = 0.1), cancer stage at diagnosis (*P* = 0.4 from Wilcoxon’s test of FIGO stage II or III versus IV), or progression-free survival (*P* = 0.8 by Cox’s proportional hazards analysis; results were similar within the subset of chemonaive cancers). Early discontinuation of seeding was associated with higher intrapatient intertumor heterogeneity, identified as a negative correlation of the mutation time of the last disseminated clone and the number of unique subclones in the ovarian versus extraovarian tumors (Spearman’s rank correlation = –0.62, *P* = 0.002; chemonaive cancers only, Spearman’s rank correlation = –0.67, *P* = 0.004). The total number of unique subclones did not increase with a high TMB (*P* > 0.9).

In most HGSCs with monophyletic polyclonal seeding (the most common dissemination mode), the dissemination appeared to occur as a single wave and within a relatively short time interval, reflecting low mutational diversity of the disseminated clones (Figure 4A). The median difference in the mutation time between the first and last disseminated clone was 0.1 (95% CI, 0.04–0.23). Polyphyletic seeding was associated with a higher mutational diversity of the disseminated clones and a longer time interval of dissemination (*P* = 0.01 by Wilcoxon’s test). A caveat of this analysis was the requirement for a different approach to estimate the relative mutation time of branched clones (estimated as the total number of unique mutations in the 2 most divergent disseminated clones, relative to the total number of modeled mutations in the cancer). Cancers exposed to neoadjuvant chemotherapy also had high mutational diversity of disseminated clones (long relative time interval of dissemination; Figure 4C), and this was associated with a poor progression-free survival among the patients (neoadjuvant chemotherapy versus chemonaive, hazard ratio, 7; *P* = 0.01 by Cox’s proportional hazards analysis).

## Discussion

Dissemination within the peritoneal cavity is a main determinant of poor patient survival from HGSC (38). The dissemination process is highly diverse (16, 17) and poorly understood from an evolutionary perspective. We modeled the subclonal architecture across tumor sites in 23 disseminated HGSCs and identified common features. The cancers were categorized according to the complexity of the dissemination process, initially separating monoclonal from polyclonal dissemination, followed by polyclonal dissemination of a linear versus branched evolutionary lineage. These categories provided a useful framework to interpret the evolutionary timing of development of disseminated subclones in relation to molecular and clinicopathological factors. In particular, HGSCs with DNA damage repair deficiency disseminated relatively late in their evolutionary trajectory models, consistent with reports of improved short-term survival and sensitivity to platinum-based chemotherapy in patients with *BRCA1* or *BRCA2* mutations (39). Our study suggested that both hereditary and somatic development of DNA repair deficiency was associated with late cancer dissemination, but the study was not powered to evaluate a potential effect on patient survival. Furthermore, polyclonal dissemination appeared to occur predominantly as a single and rapid wave of dissemination, that is, within a relatively short evolutionary time interval and with low mutational diversity of the disseminated clones. Higher diversity was observed in cancers exposed to chemotherapy, involving a longer time interval of dissemination and/or a branched evolutionary relationship of the disseminated clones (polyphyletic seeding). Previous studies have suggested that primary treatment does not change the clonal structure and complexity of most HGSCs (40). Our study supported this, and a potential effect of chemotherapy was observed on the dissemination pattern only, although the rationale for the association was not clear. Chemotherapy could exert a direct or indirect effect by eradicating sensitive subclones or by accelerating subclonal evolution (15, 25), the former being more likely in this study based on the relatively short time interval between treatment exposure and sampling. As an alternative rationale, the pattern and timing of clonal dissemination might be associated with the surgical resectability of the cancers and might have contributed to the decision to give neoadjuvant treatment in some cases. Neoadjuvant chemotherapy followed by interval debulking surgery has been shown to be noninferior to primary debulking surgery and adjuvant chemotherapy with respect to survival outcomes in randomized clinical trials (41). However, patients treated by neoadjuvant chemotherapy commonly have a high morbidity risk profile and low likelihood of complete primary cytoreduction, and they had poorer progression-free survival also in our study. The study was not sufficiently powered to resolve the potential links among chemotherapy exposure, treatment response, dissemination patterns, and patient prognosis.

Diverse models and nomenclature with partly competing views have been developed to describe the evolution of cancer metastasis (42, 43). Evidence of most models was found across the HGSCs analyzed in this study. This high degree of diversity is consistent with that found in previous studies (16, 17, 20) and with the limited physiological constraints on locoregional dissemination within the peritoneal cavity by a predominantly passive dissemination mechanism (44). Lowest complexity and monoclonal dissemination was found in cancers with a low mutation burden. However, this was not reflected in a low complexity of the subclonal architecture of the tumors. Parallel evolution after dissemination resulted in mutational heterogeneity between ovarian and extraovarian tumors, and heterogeneity was dependent on the evolutionary timing of dissemination rather than on polyclonal seeding and inheritance of genetic diversity among the sites (45). This is consistent with a parallel progression model (43), suggesting potential for greater spatial tumor heterogeneity with early dissemination. This stresses the importance of diagnosis and start of treatment as early as possible, even if the cancers have already disseminated. Most cancers in this study disseminated late in their evolutionary trajectory models, and the molecular timing of dissemination was similar to that found in another study of 6 HGSCs with predominantly metachronous metastases (24). Late evolutionary dissemination is consistent with symptom development and diagnosis relatively rapidly after cancer dissemination, and it does not translate into a potential for diagnosis and onset of treatment prior to dissemination. Single-cell multiomics sequencing has suggested that metastatic cell lineages preexist in the subclones of primary tumors (46), and there are no fundamental genomic differences between early-stage and late-stage HGSCs to aid in early diagnosis (47). Furthermore, a randomized screening study concluded that diagnosis prior to dissemination is unlikely to translate into reduced mortality, suggesting an intrinsically poor prognosis with HGSC (48).

We did not find evidence of more frequent dissemination from ovarian to extraovarian tumors, but ovarian tumors had more complex subclonal architectures. This indicated a longer time for tumor development in the ovaries, which is consistent with results found in other studies of disseminated HGSCs (40). Notably, the direction of seeding was determined in only a subset of the cancers. This partly reflects the biology and frequency of dissemination and partly the sample availability, in particular the lack of samples from the fallopian tube. Most HGSCs originate in the fallopian tube, and our analyses could not determine if the cancers spread sequentially from the tubes to the ovaries and extraovarian sites or directly from the tube to multiple intraperitoneal sites. The first scenario is most likely, based on the more complex subclonal architecture of the ovarian tumors. Our findings also supported a monoclonal origin of bilateral ovarian tumors, together with a relatively late dissemination to the contralateral ovary (49), and even evidence of bidirectional seeding between the ovaries.

Nearly three-fourths of the HGSCs had polyclonal dissemination, but the most complex pattern with polyphyletic seeding of subclones of a branched evolutionary lineage was found only in cancers with more than 2 tumor sites analyzed. Polyphyletic seeding indicates repeated acquisition of dissemination potential during cancer evolution (42). From a biological perspective, there can be a greater potential for subclonal diversity and parallel evolution when tumors grow at multiple distinct sites. However, dissemination of branched subclones occurred primarily between 2 sites in our study, and it could not be determined whether the subclones developed at the same site or at separate sites with dissemination in opposite directions. From a technical perspective, it is likely that the increased spatial resolution obtained with analysis of 3 separate sites and a larger number of samples enabled improved reconstruction of the subclonal architectures. This was the case with multiregional tumor sampling, which increased the detection rate of branched subclones within each tumor. We can, therefore, not conclude whether the underpinnings of the complex polyphyletic dissemination pattern were primarily related to distinct cancer biology or to improved spatial resolution. The lack of systematic sampling of peritoneal sites outside of the adnexa and omentum is a limitation of our study, and the frequency of polyphyletic dissemination might be underestimated by sampling of only 2 tumor sites from most patients. Additional technical caveats were related to the computational reconstruction of tumor architectures and mutation timing (50). Such models are often associated with high noise levels and potentially also with poor reproducibility. We obtained fairly correspondent tumor architecture models with two computational methods, in many cases aided by the multiple sampling approach and distinction of subclones present in different samples. Mutation time is proportional to real time under the assumption of a constant mutation rate and cell division time. However, the evolutionary timing of dissemination was correlated to the mutation burden of the cancers in this study, suggesting that the assumption did not hold true and that care should be taken in the interpretation of the mutation time beyond the estimate of mutational diversity. A main methodological advantage of the current study was the high sequencing depth. This supported the detection of rare subclones and the resolution of subclones with small differences of their cellular prevalence. Whole-genome sequencing offers broader coverage and more powerful modeling with genetic variants also outside of coding regions, but this currently comes at the cost of a lower sequencing depth. Indeed, the number of clones detected in our study was similar to that found in a recent whole-genome sequencing study with more comprehensive tumor sampling, including a median of 17 samples per cancer (25).

Polyclonal cancer origin and 2 distinct *TP53* missense mutations were identified in 1 patient with a germline mutation of *BRCA1*, but with pronounced HRD in only 1 of the 2 synchronous cancers. The frequency of polyclonal HGSC is unknown, but potential for polyclonal cancer development has been indicated in *BRCA1* mutation carriers, who commonly present with several p53 signatures and different pathogenic mutations of *TP53* at risk-reducing salpingo-oophorectomy (51). We also identified a germline variant of *NTHL1* in a patient with base excision repair-deficient tumors. This phenotype is rare and described in only 6 of >12,000 tumors across cancer types in the 100,000 Genomes Project, only 2 of which were associated with germline variants (52). The phenotype was clonal and likely developed early in the evolutionary trajectory of the cancer in this study. All somatic nonsilent mutations of *BRCA1* and *BRCA2* were also early events, consistent with a previous study reporting similar somatic mutation frequencies of these genes in early-stage and late-stage HGSCs (47). *MUC16* mutations were also identified in the initial clone of 2 cancers. This gene encodes the diagnostic biomarker CA125, which is used for blood-based monitoring of ovarian cancer (53), but the effect of mutations on disease progression and patient prognosis is not clear (54).

Activating mutations of the PI3K/AKT1 pathway were found in the disseminated subclones of 2 triple-proficient HGSCs. Overactivity of this pathway has been implicated in chemoresistance in HRD-positive HGSCs (25), but the interpretation of a treatment prediction value in the context of HR proficiency and generally low platinum sensitivity is not clear. The *AKT1*^E17K^ mutated cancer was chemorefractory, but the patient with a *PIK3CA*^E545Q^ mutation had long-term progression-free survival after adjuvant chemotherapy. The latter cancer also had *CCNE1* amplification, which is commonly mutually exclusive and synthetically lethal with HRD (55). Therapeutic inhibition of the PI3K/AKT1 pathway has been proposed as a strategy to disrupt HR and sensitize cancer cells to PARP inhibition (56), and a nonrandomized trial of the pan-AKT inhibitor capivasertib suggested that AKT1^E17K^ is a therapeutic target in metastatic solid tumors (57). Furthermore, PI3K/AKT1 inhibition has been shown to be effective in HGSC organoids of a specific evolutionary state characterized by multiple subclones, polyclonal seeding, and frequent PI3K/AKT1 pathway aberrations (40). Consistently, both HGSCs in our study with activating mutations of this pathway had polyphyletic seeding. However, the mutations were found in only 1 of several disseminated clones, and targeted inhibition would likely have had limited efficacy. One additional tumor suppressor gene was predicted to be recurrently involved in cancer development or dissemination. Hotspot mutation of the DNA methyl transferase-encoding gene *DNMT3A* is a frequent and early event associated with poor patient survival in myeloid leukemias (58). The mutations detected in this study were not in the same hotspot region, but mutations of other loci have been proposed as risk factors for development of secondary myeloid neoplasms after PARP inhibition for ovarian cancer (59). None of the patients in this study received PARP inhibitors, and the patient with a pathogenic missense mutation was also chemonaive, suggesting that the mutations are not exclusively treatment induced. It has been suggested that knockdown of DNMT3A can attenuate the proliferation and invasiveness of ovarian cancer cells (60), but the impact of mutations in this respect is unknown. In general, the collection of mutations in the initial and disseminated cancer clones varied largely among the patients. This is consistent with the low prevalence of most mutations across HGSCs (61) and underlines the challenge of identifying common treatment targets beyond the HRD phenotype. Frequent polyclonal dissemination adds to this challenge and is consistent with observations in end-stage and HRD-positive HGSCs that the mechanisms of resistance to platinum-based chemotherapy are frequently subclonal and diverse across metastatic sites (25). Multiomics approaches and integrated characterization of tumor phenotypes on the transcriptomic and epigenomic levels with tumor microenvironment components are likely needed to aid in the delineation of the translational relevance of evolutionary models (40).

In summary, this study describes a complex and diverse subclonal dissemination pattern of HGSCs. Disseminated clones developed relatively late in the evolutionary trajectory models of most of the cancers, in particular in cancers with DNA damage repair deficiency. Dissemination was predominantly polyclonal but occurred as a single and rapid wave of clones with relatively low mutational diversity and within a short evolutionary time interval, except in cancers exposed to chemotherapy.

## Methods

### Sex as a biological variable.

This study investigated cancer of the female reproductive system.

### Patients and tumor samples.

Fresh frozen tumor (*n* = 104) and ascites (*n* = 4) samples were collected from patients (*n* = 23) treated by cytoreductive (debulking) surgery for disseminated HGSC at The Norwegian Radium Hospital, Oslo University Hospital, between 2002 and 2012. Tumor sampling was performed of surgical specimens submitted to the Department of Pathology, and all samples were collected at a single surgery of each patient. The surgical specimens included most commonly the adnexa and omentum, and other peritoneal sites were sampled in a minority of cases. Patients (Supplemental Table 1) were selected based on the availability of multiple tumor samples from the ovaries and extraovarian sites. The median number of cancer samples per patient was 5 (range, 3–7), including 2–4 tumor samples from the ovaries and 1–5 extraovarian tumor or ascites samples (except 1 patient with bilateral ovarian samples only; Supplemental Figure 1 and Supplemental Table 2). Samples from 3 different sites were available from 7 patients. Bilateral ovarian tumors (*n* = 3 patients) and ascites were both considered separate sites. Blood samples were used for reference and collected at the time of diagnosis before (*n* = 14 patients) or after (*n* = 4) first-line treatment or at a median of 4 years (range, 2–6 years) after diagnosis (*n* = 5).

Patients were treated according to national guidelines and readmitted to the hospital at relapse. Primary treatment consisted of radical debulking surgery with the aim of less than 1 cm rest tumor, followed by 6 courses of chemotherapy by paclitaxel and carboplatin every 3 weeks (*n* = 17 patients), or neoadjuvant chemotherapy with 3 courses before and after interval debulking surgery (*n* = 6 patients; Supplemental Table 1). Interval debulking surgery was performed at a mean of 85 days after chemotherapy (range, 67–117 days). Patients treated by primary debulking surgery were chemonaive at the time of sampling. All primary pathology diagnostics were performed by experienced gynecologic pathologists, and clinical data were extracted from the patients’ medical records. Evaluation of response to therapy was based on computerized tomography scans of the thorax, abdomen, and pelvis as well as the tumor marker CA125 (53). After neoadjuvant chemotherapy, partial response was observed in 4 patients, stable disease in 1, and decrease of CA125 levels from 800 to 43 units/mL in 1; 1 patient was undetermined. After first-line treatment, 3 patients had refractory disease, 17 had progression after a median of 10 months (range, 2–33 months), and 3 were progression free after a median of 9 years (range, 3–10 years; Supplemental Table 1).

DNA was isolated using the AllPrep DNA/RNA/miRNA Universal kit according to the manufacturer’s instructions (Qiagen). H&E stains of all fresh frozen tissue samples were evaluated by a specialist in gynecologic pathology (BD) to confirm the diagnosis of HGSC and to evaluate tumor purity of the samples (median 70%, 10–90 percentile range 40%–90%; Supplemental Table 2 and Supplemental Figure 3A). Available diagnostic specimens of the distal fallopian tube (from all patients) with fimbriae (7 patients) were retrospectively evaluated, and a tubal cancer origin was confirmed in 6 (75%) of 8 evaluable patients, including 1 patient with fused tube and ovary (Supplemental Table 1; representative images are shown in Supplemental Figure 2A). The remaining cases were inconclusive for tubal versus ovarian cancer origin. Morphological evaluations indicated angiogenesis and a varying extent of solid growth patterns in the diagnostic specimens of all included tumor samples, but the solid, pseudoendometrioid, transitional cell carcinoma-like morphology (SET feature) was not observed. The density of lymphocytes and fibroblasts was scored in 3 groups (Supplemental Table 2).

### Whole-exome sequencing and mutation calling.

Whole-exome sequencing was performed to a mean depth of 668× coverage of tumor samples (range, 365–806) and 736× coverage of blood samples (range, 657–828; Supplemental Table 2). Exome libraries were generated from 1 μg genomic DNA using the Agilent SureSelect All Exon v6+COSMIC capture kit. Sequencing was performed in 2 × 100 base-pair paired-end mode using the Illumina HiSeq 4000 System. Raw sequencing reads were aligned to the human reference genome hg38 using BWA (version 0.7.8) (62). Sequence alignments were converted to binary files with Picard (version 1.102; http://broadinstitute.github.io/picard) and sorted and indexed with SAMtools (version 0.1.19) (63). Binary alignment map (BAM) files were preprocessed following the Genome Analysis Toolkit (version 3.8; https://gatk.broadinstitute.org/hc/en-us). Somatic SNVs were called with MuTect (version 1.1.7) (64) and indels with Strelka (version 1.0.14) (65). Germline variants were called with Haplotypecaller (version 3.6) (66). All variants were annotated using ANNOVAR (version 2015-12-14) (67) and the Ensembl Variant Effect Predictor (version 79). Candidate somatic mutations were filtered to include loci with ≥15× coverage in the tumor- and patient-matched blood samples as well as mutant allele fractions ≥5% in the tumor sample and <1% in the blood sample. To accommodate the high sequencing depth, a mutant allele fraction up to 5% in the blood sample was accepted for candidate loci with a fraction ≥15% in the tumor sample and ≥100× coverage in both the tumor and blood sample, if the mutant allele fraction was also ≥4 times higher in the tumor sample than in the blood (to allow for the presence of circulating tumor cells and potential technical errors). Quality control was performed with Samtools, and only reads with mapping quality ≥30 were included for analysis. Germline variants were filtered to include loci with ≥10× coverage, ≥5 variant reads, and a variant allele fraction ≥5%.

Nonsynonymous exonic SNVs (missense, nonsense, stoploss), frameshift indels, and splice site mutations (SNVs or indels) were considered nonsilent. A total of 12,618 somatic SNVs and 1,008 somatic indels were identified, including 9,449 nonsilent SNVs and 821 nonsilent indels (Supplemental Table 2). Classification of oncogenes and/or tumor suppressor genes was adopted from the CGC (tier 1 and 2; downloaded from https://cancer.sanger.ac.uk/census in March 2022) (68) if based on the relevant mutation types (missense, nonsense, frameshift, or splice site mutations). Mutated genes were “flagged” if included in a list of genes (*n* = 100) with frequent mutations across exome sequencing studies and associated with common features such as a long protein coding sequence and a large number of paralogs (69).

Variants of selected cancer predisposing genes involved in DNA damage repair were annotated for pathogenicity using curated databases provided by the ARUP Laboratories and the University of Utah Department of Pathology for *BRCA1* and *BRCA2* (accessed in April 2022, now available from https://www.ncbi.nlm.nih.gov/clinvar/; accessed in April 2022) or the InSiGHT (70) (https://www.insight-database.org/classifications/) and LOVD databases for *MLH1*, *MLH3*, *MSH2*, *MSH6*, and *PMS2* (https://databases.lovd.nl/shared/variants; both accessed in April 2022). Germline variants of *BRCA1* and *BRCA2* have previously been confirmed by Sanger sequencing (71). The effect of selected somatic SNVs on protein structure and function was predicted using the web interface of PolyPhen-2 (http://genetics.bwh.harvard.edu/pph2/; accessed in June 2023).

### DNA copy number estimation.

Allele-specific DNA copy numbers and tumor purity were estimated from BAM files of patient-matched tumor and blood samples using the R package FACETS with default settings (72). The estimated tumor purity per sample was correlated to the histopathological evaluations (Spearman’s rank correlation = 0.6, *P* = 1 × 10^–11^; Supplemental Figure 3A). DNA copy number aberrations were called based on median-centered copy numbers above or below 0, and aberration frequencies were plotted with the cnFreq function in GenVisR (73). Loss of heterozygosity was called in segments with a minor allele copy number of 0.

### Mutation signatures.

COSMIC single base substitution signatures (26) were estimated with the R package SomaticSignatures (74). The contribution of each substitution type and its two surrounding bases was scored relative to the COSMIC reference signatures (v3.2; https://cancer.sanger.ac.uk/signatures/downloads) using the R package MutationalPatterns (v3.4.1) (75). Values were presented as the proportion of each signature per sample. MSI status was determined by the PCR-based Promega MSI Analysis assay in accordance with the manufacturer’s protocol.

### Sample-wise clonality estimates.

MutationTimeR (32) was run with default settings to estimate the timing of SNVs and indels relative to the local DNA copy numbers per sample and to assign clonal (early, late, NA) and subclonal states to each mutation. The distinction between clonal and subclonal mutation calls according to this algorithm is based on the estimated presence in all or a fraction of cancer cells. The distinction between early and late clonal mutations is based on the occurrence before or after local copy number gains. To summarize the frequencies of each mutation category per gene (clonal or subclonal), genes with several nonsilent mutations were counted once per category per sample. Mutations annotated as clonal in at least 1 sample were considered true clonal if also found in all tumor samples from the patient (otherwise categorized as subclonal).

### Patient-wise clonality models.

Inference of subclonal tumor populations based on mutation clustering and clonal ordering was performed with PyClone version 0.13.0 (33) and ClonEvol version 0.99.11 (34), respectively, on somatic SNVs and indels detected in any tumor sample per patient. Additional preprocessing of mutations prior to modeling was performed with MuTect2 (v4.1.2.0; https://gatk.broadinstitute.org/hc/en-us/articles/360037593851-Mutect2) to enable joint mutation calling on all samples from each patient. BAM files were used as input together with browser extensible data files of regions of the previously called mutations (±20 base pairs), to reduce processing time. Only mutations detected by both MuTect2 and MuTect/Strelka after the previously described filtering criteria were used. Patient-wise mutation files with information regarding mutant allele fractions were merged with the allele-specific copy number files from FACETS and used as input for PyClone to model putative clones and their cellular prevalence based on the adjusted allelic fraction of mutation clusters (adjusted for DNA copy numbers and tumor purity). PyClone was run with default parameters, with the exception of a minimum mutation cluster size of 3 (--min_cluster_size 3) and 10,000 iterations (--num_iters 10000). The optional parameter --purity was assigned with the estimated tumor purity from FACETS. Reconstruction of subclonal architectures based on cellular prevalence estimates was further performed with ClonEvol using the function infer.clonal.models and default parameters, except the parameter cluster.center was set to “mean” and num.boots was set to 100. The number of bootstraps was lowered to reduce the computation time, which was high due to the large sample number per patient and a high level of heterogeneity resulting in a large number of subclones. This had little impact on the results (data not shown). The parameter cancer.initiation.model was initially set to “monoclonal” and changed to “polyclonal” if unsuccessful. If still unsuccessful, conflicting clones were identified by a step-wise exclusion approach with a manual evaluation of all clones. Conflicting clones or mutation clusters with poor quality were adjusted or excluded. Clones with a similar estimated cellular prevalence, but with slight variation relative to each other across the individual samples from each patient, were estimated from the relationship established in the majority of samples, and slight adjustments were made to allow inclusion in the final models. Manual filtering and adjustment of subclones are summarized for each cancer in Supplemental Table 4. The clone with an estimated cellular prevalence of approximately 1 across all samples per cancer (after adjusting for tumor purity) was designated as the initial (truncal) clone. Clones present at a lower and/or varying cellular prevalence across tumor sites were designated as disseminated, and clones present in a single tumor site as local.

Multiple possible clonality models were identified for each patient, and a step-wise approach was used to identify the best fit. Initially, all samples from each patient were analyzed separately with PyClone and ClonEvol to obtain a full patient model and to retain the best possible spatial resolution. The relationship among subclones (parent, progeny, unrelated) was established in this step and used to inform the final models, which were presented as the mean per tumor site per patient. It was not possible to obtain full models for all patients with more than 2 tumor sites, and these models were presented as multiple pair-wise comparisons (producing up to 3 pair-wise comparisons in patients with 3 tumor sites). In cases retaining multiple possible models after this approach, the least complex and most parsimonious model was selected for presentation (selecting linear over branched evolution, and dissemination of a single clone over multiple). The total number of estimated subclones per patient was partly dependent on subtle differences in the cellular prevalence of mutation clusters, and therefore prone to inaccuracy. However, mutation clusters and clonality models were subjected to thorough manual inspections, and all subclones relevant for the overall conclusions regarding the mode and direction of dissemination were robustly identified (models prone to inaccuracy are described in Supplemental Figures 11–13).

Final models were visualized as fish plots with the R package fishplot (76). The phylogeny of each cancer was inferred from the fish plots and illustrated as a horizontal dot plot of the relative timing of development of each clone (proportion of mutations in the clone relative to the total number of mutations in the cancer model). The direction of seeding was determined based on the cellular prevalence of the disseminating clone, and the site with the smallest prevalence was considered the original. A single bidirectional arrow illustrated that the direction was not possible to determine.

The evolutionary timing of dissemination was evaluated in mutation time, representing the number of mutations in each disseminated subclone relative to the latest developed subclone per cancer (the subclone with the largest number of mutations).

Subclone predictions and phylogenetic inferences were also performed with LICHeE version 1.0 for comparison with PyClone and ClonEvol (35). LICHeE does not incorporate adjustment of mutant allele fractions based on DNA copy numbers or tumor purity, and adjusted values for each mutation were imported from PyClone, as recommended. LICHeE was run with default parameters, except that --minClusterSize was set to 3 to match the PyClone runs. Furthermore, --maxClusterDist was lowered to prevent excessive merging of subclones, and --maxVAFAbsent and --minVAFPresent were adjusted to prevent modeling failure. No combinations of parameter values allowed modeling of all cancers, and final values are listed in Supplemental Table 5 (values close to default were prioritized). Phylogenetic inferences from LICHeE were plotted using the as.Node function in the R package data.tree (version 1.0.0). Alluvial diagrams to compare the mutation clustering/subclone predictions with PyClone were plotted with the R package alluvial (version 0.1–2).

### Tumor microenvironment estimates.

Stromal and immune infiltration scores of each tumor sample were estimated with the R package ESTIMATE version 1.0.13 on total RNA-sequencing data (37). RNA sequencing was performed in 2 × 101 base-pair paired-end mode on the Illumina NovaSeq 6000 System to a mean depth of 70.4 × 10^6^ uniquely mapped reads per sample. Sample preparation was performed with the Ribo-Zero Gold rRNA removal kit (Illumina), and sequence library generation was performed with the TruSeq Stranded Total RNA Library Prep Gold kit (Illumina). Paired-end reads were trimmed with TRIMMOMATIC version 0.38 and aligned to the GRCh38 human reference genome using STAR version 2.7.6a. Reads mapping to protein-coding genes were quantified using HTseq-count v.2.0.2, and gene expression estimates were normalized as fragments per kilobase of transcripts per million mapped reads (FPKM). The ESTIMATE scores are provided in Supplemental Table 2. The stromal infiltration scores correlated with tumor purity evaluations and fibrosis scores from histopathology and the immune infiltration scores with lymphocyte scores, but both gene expression estimates varied according to tumor site and previous chemotherapy exposure (Supplemental Figure 3).

### Statistics.

Statistical analyses were performed in R (v3.6.1). Two-sided *P* values of less than 0.05 were considered significant. Spearman’s and Pearson’s correlation tests were performed with the cor.test function. Wilcoxon’s rank sum test and *t* tests (2 tailed) were run with wilcox.test and t.test. Fisher’s exact test and calculation of odds ratios were performed with fisher.test. CIs of the median were calculated using DescTools. In box plots, bounds of boxes represent the interquartile range, the lines within boxes represent the median, and whiskers represent 1.5× the interquartile range above the 75th percentile or below the 25th percentile. Principal components analysis was performed with the PCA function in FactoMineR (77) on a matrix representing the proportion of each COSMIC base substitution signature per sample (filtered to include only signatures with absolute value of ≥10 in at least 1 sample) and on gene-wise DNA copy number estimates (filtered to include the 2,000 genes with largest cross-sample variance). Patient survival was analyzed with progression-free survival as the endpoint, estimated from the last day of first-line therapy (surgery and chemotherapy) to cancer recurrence or progression diagnosed by radiology or increased blood levels of CA125. Cox proportional hazards were estimated with the coxph function in the survminer package and *P* values from Wald test.

### Study approval.

The project and patient consent for genomic analyses were approved by the Regional Committee for Medical and Health Research Ethics South East Norway (REC no. 2014/473). Samples were registered at the Biobank Registry of Norway for blood (S-01188) or tumor tissue (S-04300). All patients provided written informed consent, or exemption from consent was approved for deceased patients.

### Data availability.

Supporting data for each figure panel are available in the Supporting Data Values file. In accordance with Norwegian legislation and the ethical approval of the study by the Regional Committee for Medical and Health Research Ethics South East Norway, the raw high-throughput sequencing data generated in this study are considered patient identifiable and subject to secure storage regulations in accordance with the national Personal Data Regulations, chapter 2. Data can currently not be deposited to public repositories. Data will be made available upon reasonable request to AS, and this will require formalization of a data transfer agreement. All analyses were performed with published software packages and computer code and are described in the Methods.

## Author contributions

AS, AD, BD, and RAL provided study conception and design. AS, BJ, SMKK, SMK, KT, RIS, AD, BD, and RAL provided data analysis and interpretation. AS, BJ, SMKK, SMK, LAMZ, MB, and OM processed samples and acquired data. KB, AD, and BD acquired patient samples and histological and clinical annotations. AS wrote the manuscript draft. RAL supervised the study. All authors read and approved the final manuscript.

## Supplementary Material

Supplemental data

Supplemental tables 1-5

Supporting data values

## Figures and Tables

**Table 1:**
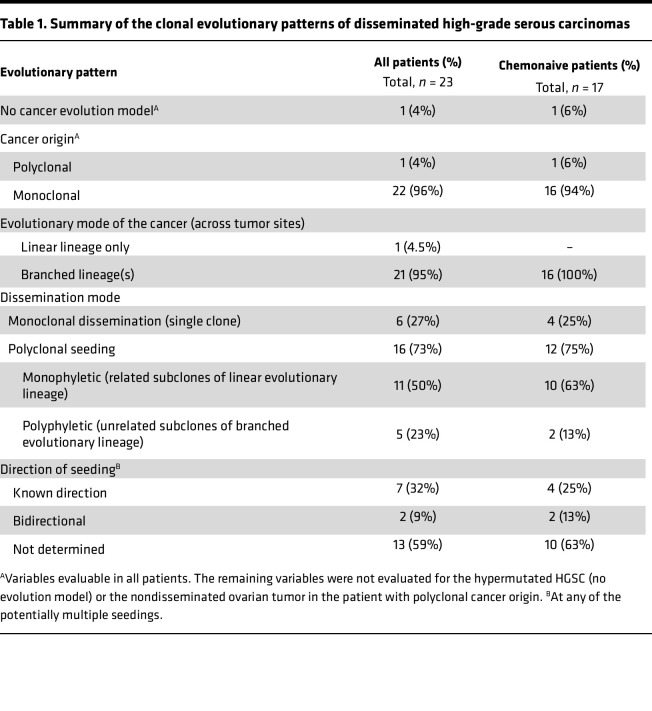
Summary of the clonal evolutionary patterns of disseminated high-grade serous carcinomas

## Version 1 01/04/2024

In-Press Preview

## Version 2 02/08/2024

Electronic publication

## References

[1] Berek JS (2021). Cancer of the ovary, fallopian tube, and peritoneum: 2021 update. Int J Gynaecol Obstet.

[2] Ducie J (2017). Molecular analysis of high-grade serous ovarian carcinoma with and without associated serous tubal intra-epithelial carcinoma. Nat Commun.

[3] Naora H, Montell DJ (2005). Ovarian cancer metastasis: integrating insights from disparate model organisms. Nat Rev Cancer.

[4] Gockley A (2017). Outcomes of women with high-grade and low-grade advanced-stage serous epithelial ovarian cancer. Obstet Gynecol.

[5] Labidi-Galy SI (2017). High grade serous ovarian carcinomas originate in the fallopian tube. Nat Commun.

[6] Shih IM (2021). The origin of ovarian cancer species and precancerous landscape. Am J Pathol.

[7] Wu RC (2019). Genomic landscape and evolutionary trajectories of ovarian cancer precursor lesions. J Pathol.

[8] Lee Y (2007). A candidate precursor to serous carcinoma that originates in the distal fallopian tube. J Pathol.

[9] Macintyre G (2018). Copy number signatures and mutational processes in ovarian carcinoma. Nat Genet.

[10] Wang YK (2017). Genomic consequences of aberrant DNA repair mechanisms stratify ovarian cancer histotypes. Nat Genet.

[11] Oza AM (2019). Advances in prediction for ovarian cancer treatment stratification. Nat Rev Clin Oncol.

[12] González-Martín A (2019). Niraparib in patients with newly diagnosed advanced ovarian cancer. N Engl J Med.

[13] Patch AM (2015). Whole-genome characterization of chemoresistant ovarian cancer. Nature.

[14] Christie EL, Bowtell DDL (2017). Acquired chemotherapy resistance in ovarian cancer. Ann Oncol.

[15] Kim S (2018). Tumor evolution and chemoresistance in ovarian cancer. NPJ Precis Oncol.

[16] McPherson A (2016). Divergent modes of clonal spread and intraperitoneal mixing in high-grade serous ovarian cancer. Nat Genet.

[17] Eckert MA (2016). Genomics of ovarian cancer progression reveals diverse metastatic trajectories including intraepithelial metastasis to the fallopian tube. Cancer Discov.

[18] Motohara T (2019). An evolving story of the metastatic voyage of ovarian cancer cells: cellular and molecular orchestration of the adipose-rich metastatic microenvironment. Oncogene.

[19] Zhang AW (2018). Interfaces of malignant and immunologic clonal dynamics in ovarian cancer. Cell.

[20] Bashashati A (2013). Distinct evolutionary trajectories of primary high-grade serous ovarian cancers revealed through spatial mutational profiling. J Pathol.

[21] Schwarz RF (2015). Spatial and temporal heterogeneity in high-grade serous ovarian cancer: a phylogenetic analysis. PLoS Med.

[22] Li C (2019). Mutational landscape of primary, metastatic, and recurrent ovarian cancer reveals c-MYC gains as potential target for BET inhibitors. Proc Natl Acad Sci U S A.

[23] Paracchini L (2021). Regional and temporal heterogeneity of epithelial ovarian cancer tumor biopsies: implications for therapeutic strategies. Oncotarget.

[24] Masoodi T (2020). Genetic heterogeneity and evolutionary history of high-grade ovarian carcinoma and matched distant metastases. Br J Cancer.

[25] Burdett NL (2023). Multiomic analysis of homologous recombination-deficient end-stage high-grade serous ovarian cancer. Nat Genet.

[26] Alexandrov LB (2020). The repertoire of mutational signatures in human cancer. Nature.

[27] Batalini F (2022). Mutational signature 3 detected from clinical panel sequencing is associated with responses to olaparib in breast and ovarian cancers. Clin Cancer Res.

[28] Grolleman JE (2019). Mutational signature analysis reveals NTHL1 deficiency to cause a multi-tumor phenotype. Cancer Cell.

[29] Konstantinopoulos PA (2015). Homologous recombination deficiency: exploiting the fundamental vulnerability of ovarian cancer. Cancer Discov.

[30] Stronach EA (2018). Biomarker assessment of HR deficiency, tumor BRCA1/2 mutations, and CCNE1 copy number in ovarian cancer: associations with clinical outcome following platinum monotherapy. Mol Cancer Res.

[31] Graf RP (2021). Association of copy number variation signature and survival in patients with serous ovarian cancer. JAMA Netw Open.

[32] Gerstung M (2020). The evolutionary history of 2,658 cancers. Nature.

[33] Roth A (2014). PyClone: statistical inference of clonal population structure in cancer. Nat Methods.

[34] Dang HX (2017). ClonEvol: clonal ordering and visualization in cancer sequencing. Ann Oncol.

[35] Popic V (2015). Fast and scalable inference of multi-sample cancer lineages. Genome Biol.

[36] Ahmadinejad N (2022). Accurate identification of subclones in tumor genomes. Mol Biol Evol.

[37] Yoshihara K (2013). Inferring tumour purity and stromal and immune cell admixture from expression data. Nat Commun.

[38] Kurman RJ, Shih I-M (2016). The dualistic model of ovarian carcinogenesis: revisited, revised, and expanded. Am J Pathol.

[39] Candido-dos-Reis FJ (2015). Germline mutation in BRCA1 or BRCA2 and ten-year survival for women diagnosed with epithelial ovarian cancer. Clin Cancer Res.

[40] Lahtinen A (2023). Evolutionary states and trajectories characterized by distinct pathways stratify patients with ovarian high grade serous carcinoma. Cancer Cell.

[41] Wright AA (2016). Neoadjuvant chemotherapy for newly diagnosed, advanced ovarian cancer: society of gynecologic oncology and American Society of Clinical Oncology Clinical Practice Guideline. J Clin Oncol.

[42] Birkbak NJ, McGranahan N (2020). Cancer genome evolutionary trajectories in metastasis. Cancer Cell.

[43] Naxerova K, Jain RK (2015). Using tumour phylogenetics to identify the roots of metastasis in humans. Nat Rev Clin Oncol.

[44] Lengyel E (2010). Ovarian cancer development and metastasis. Am J Pathol.

[45] Heyde A (2019). Consecutive seeding and transfer of genetic diversity in metastasis. Proc Natl Acad Sci U S A.

[46] Wang Y (2022). Single-cell dissection of the multiomic landscape of high-grade serous ovarian cancer. Cancer Res.

[47] Cheng Z (2022). The genomic landscape of early-stage ovarian high-grade serous carcinoma. Clin Cancer Res.

[48] Menon U (2021). Ovarian cancer population screening and mortality after long-term follow-up in the UK Collaborative Trial of Ovarian Cancer Screening (UKCTOCS): a randomised controlled trial. Lancet.

[49] Micci F (2010). Tumor spreading to the contralateral ovary in bilateral ovarian carcinoma is a late event in clonal evolution. J Oncol.

[50] Turajlic S (2015). Inferring mutational timing and reconstructing tumour evolutionary histories. Biochim Biophys Acta.

[51] Akahane T (2022). TP53 variants in p53 signatures and the clonality of STICs in RRSO samples. J Gynecol Oncol.

[52] Degasperi A (2022). Substitution mutational signatures in whole-genome-sequenced cancers in the UK population. Science.

[53] Salani R (2017). An update on post-treatment surveillance and diagnosis of recurrence in women with gynecologic malignancies: Society of Gynecologic Oncology (SGO) recommendations. Gynecol Oncol.

[54] Felder M (2014). MUC16 (CA125): tumor biomarker to cancer therapy, a work in progress. Mol Cancer.

[55] Etemadmoghadam D (2013). Synthetic lethality between CCNE1 amplification and loss of BRCA1. Proc Natl Acad Sci U S A.

[56] Ibrahim YH (2012). PI3K inhibition impairs BRCA1/2 expression and sensitizes BRCA-proficient triple-negative breast cancer to PARP inhibition. Cancer Discov.

[57] Kalinsky K (2021). Effect of capivasertib in patients with an AKT1 E17K-mutated tumor: NCI-MATCH subprotocol EAY131-Y nonrandomized trial. JAMA Oncol.

[58] Brunetti L (2017). DNMT3A in leukemia. Cold Spring Harb Perspect Med.

[59] Chiusolo P (2022). A common pattern of somatic mutations in t-MDS/AML of patients treated with PARP inhibitors for metastatic ovarian cancer. Am J Hematol.

[60] Lu J (2022). Downregulation of DNMT3A attenuates the Warburg effect, proliferation, and invasion via promoting the inhibition of miR-603 on HK2 in ovarian cancer. Technol Cancer Res Treat.

[61] The Cancer Genome Atlas Research Network (2011). Integrated genomic analyses of ovarian carcinoma. Nature.

[62] Li H, Durbin R (2009). Fast and accurate short read alignment with Burrows-Wheeler transform. Bioinformatics.

[63] Danecek P (2021). Twelve years of SAMtools and BCFtools. Gigascience.

[64] Cibulskis K (2013). Sensitive detection of somatic point mutations in impure and heterogeneous cancer samples. Nat Biotechnol.

[65] Saunders CT (2012). Strelka: accurate somatic small-variant calling from sequenced tumor-normal sample pairs. Bioinformatics.

[67] Wang K (2010). ANNOVAR: functional annotation of genetic variants from high-throughput sequencing data. Nucleic Acids Res.

[68] Futreal PA (2004). A census of human cancer genes. Nat Rev Cancer.

[69] Shyr C (2014). FLAGS, frequently mutated genes in public exomes. BMC Med Genomics.

[70] Thompson BA (2014). Application of a 5-tiered scheme for standardized classification of 2,360 unique mismatch repair gene variants in the InSiGHT locus-specific database. Nat Genet.

[71] Bjørnslett M (2012). Effect of the MDM2 promoter polymorphisms SNP309T>G and SNP285G>C on the risk of ovarian cancer in BRCA1 mutation carriers. BMC Cancer.

[72] Shen R, Seshan VE (2016). FACETS: allele-specific copy number and clonal heterogeneity analysis tool for high-throughput DNA sequencing. Nucleic Acids Res.

[73] Skidmore ZL (2016). GenVisR: genomic visualizations in R. Bioinformatics.

[74] Gehring JS (2015). SomaticSignatures: inferring mutational signatures from single-nucleotide variants. Bioinformatics.

[75] Blokzijl F (2018). MutationalPatterns: comprehensive genome-wide analysis of mutational processes. Genome Med.

[76] Miller CA (2016). Visualizing tumor evolution with the fishplot package for R. BMC Genomics.

[77] Le S (2008). FactoMineR: an R package for multivariate analysis. J Stat Softw.

