# Supplementary material for: Evolutionary mode and timing of dissemination of high-grade serous carcinomas
Source: JCI Insight. 2024 Jan 4;9(3):e170423. doi: 10.1172/jci.insight.170423 (PMC11143962; doi:10.1172/jci.insight.170423)
Supplement: Supplemental data [file jciinsight-9-170423-s040.pdf]

Supplemental Figures S1 to S26 for:

Evolutionary mode and timing of dissemination of high-grade serous carcinomas

Anita Sveen, Bjarne Johannessen, Solveig M. K. Klokkerud, Sigrid M. Kraggerud, Leonardo A. Meza-Zepeda, Merete Bjørnslett, Katharina Bischof, Ola Myklebost, Kjetil Taskén, Rolf I. Skotheim, Anne Dørum, Ben Davidson, Ragnhild A. Lothe

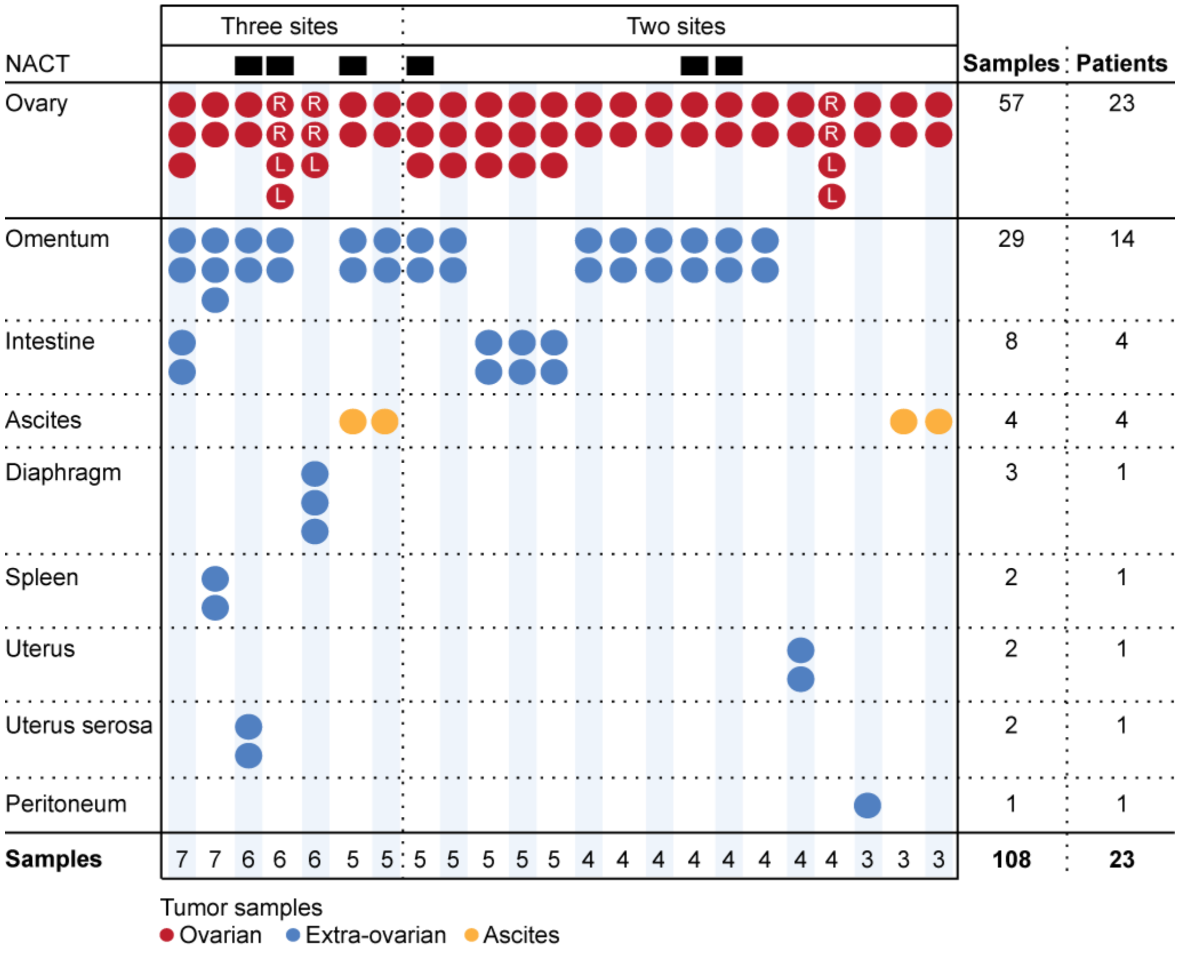

Supplemental Figure S1. Overview of tumor samples

Tumor samples (dots) are grouped by patient (columns) and tumor site (rows). Samples from three tumor sites were available from seven patients, and the remaining 16 patients had samples from two tumor sites. Bilateral ovarian tumor samples were available from three patients (L: left ovary; R: right ovary). Six patients received neoadjuvant chemotherapy (NACT, black boxes in upper row).

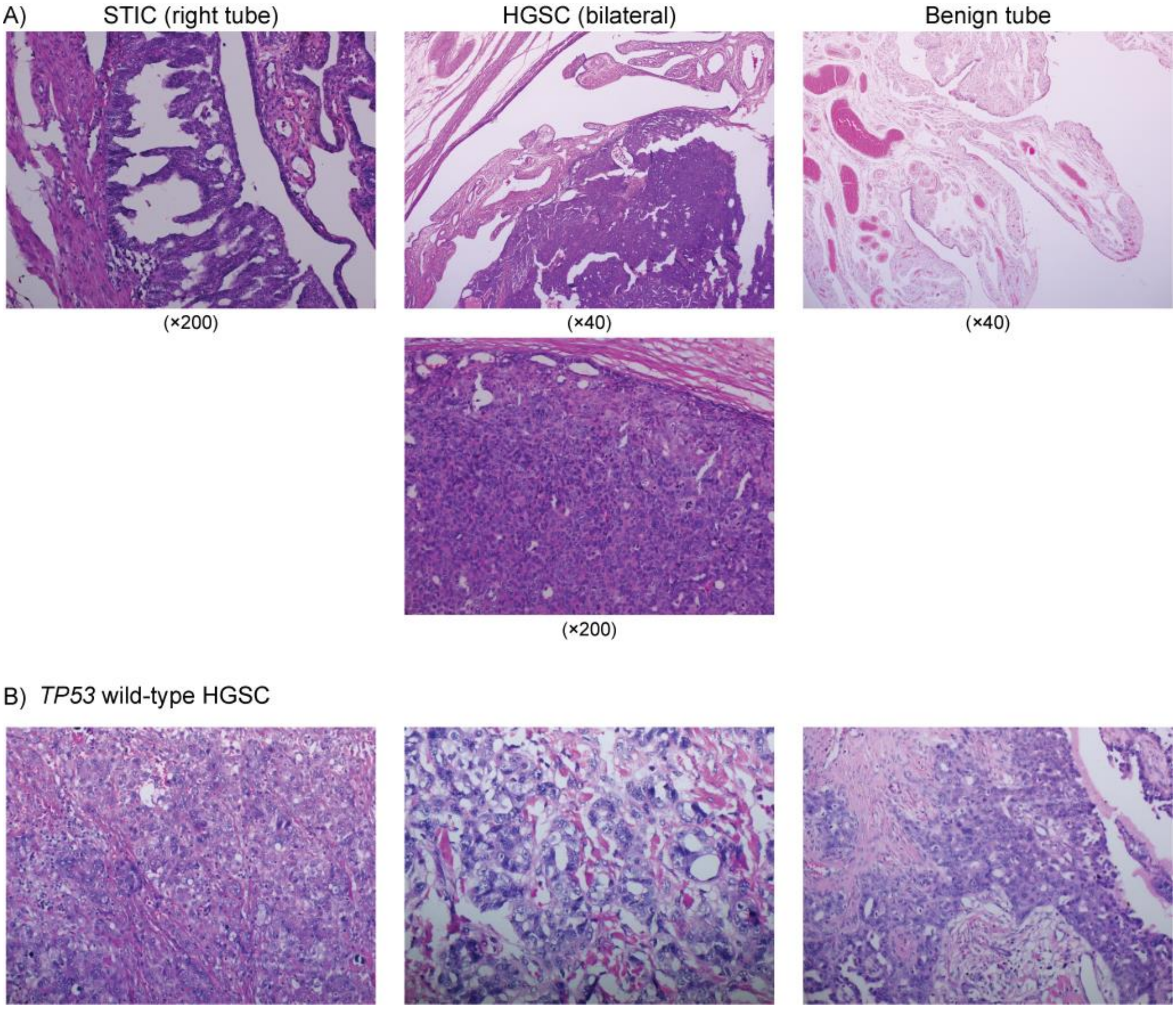

Supplemental Figure S2. Pathology evaluations of fallopian tube samples and the *TP53* wild-type HGSC

(A) Representative hematoxylin and eosin (HE stains) of diagnostic samples of fallopian tubes with serous tubal intraepithelial carcinoma (STIC; left panel), high-grade serous carcinoma (HGSC; middle panel), and a normal morphology (right panel) at time of diagnosis. The patient with benign fallopian tubes had cancer origin in the ovary (not shown). (B) HE stains confirm the diagnosis of HGSC in the *TP53* wild-type cancer.

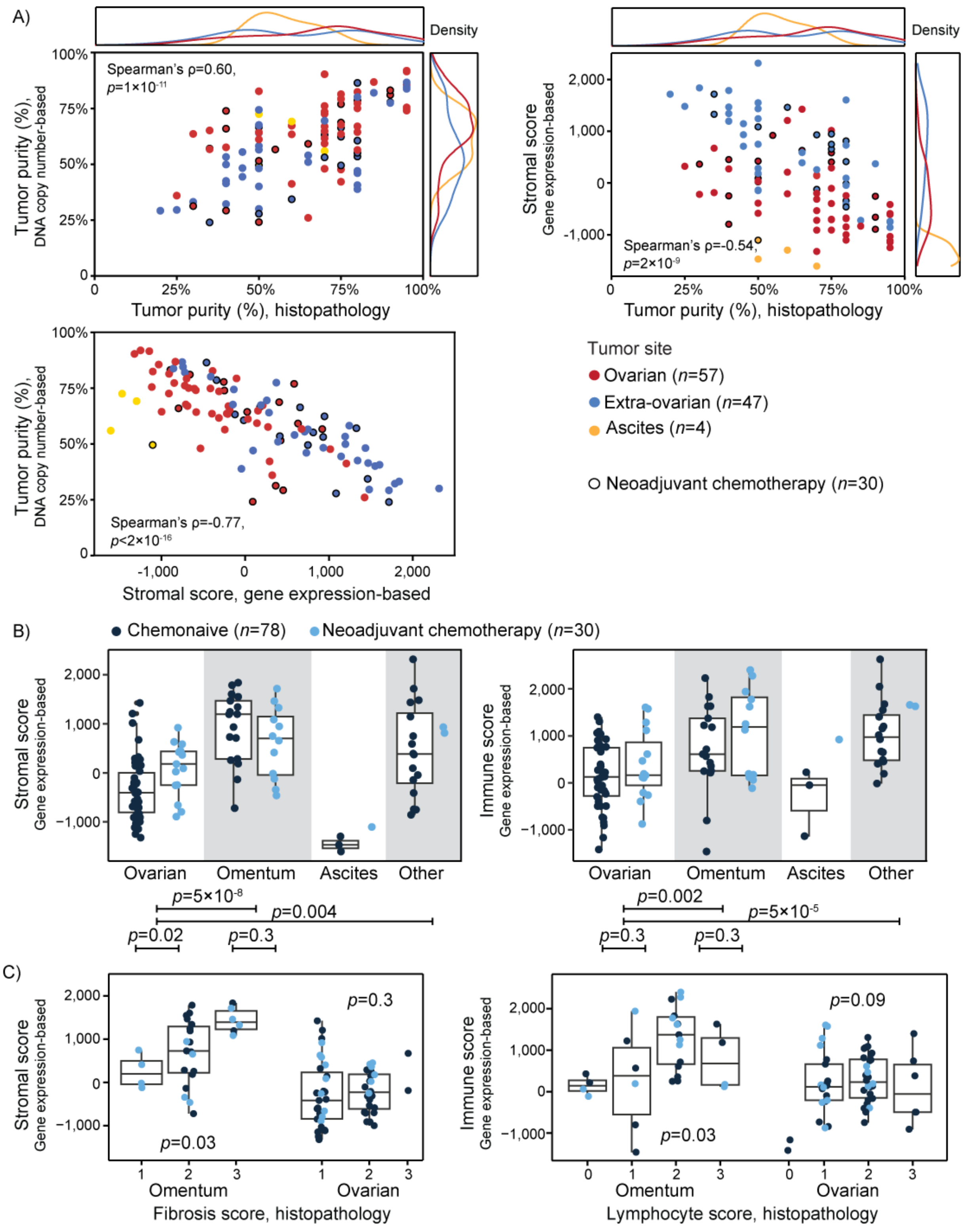

Supplemental Figure S3. Tumor sample purity

(A) Correlation of tumor purity estimates based on histopathology (evaluation of hematoxylin and eosin stains of fresh frozen tissue sections), allele-specific DNA copy number data (FACETS) and gene expression (ESTIMATE). There was no difference in histopathology-based tumor purity estimates between ovarian and extra-ovarian tumors ($p > 0.1$; Welch's t-test). (B) Gene expression-based stromal and immune scores varied according to tumor site and prior chemotherapy exposure. *p*-

values are from Welch's t-test. (C) Comparisons of stromal and immune scores from histopathology (fibrosis and lymphocyte scores) and gene expression (ESTIMATE scores) among tumors in the omentum and ovary separately. *p*-values are from Kruskal-Wallis test.

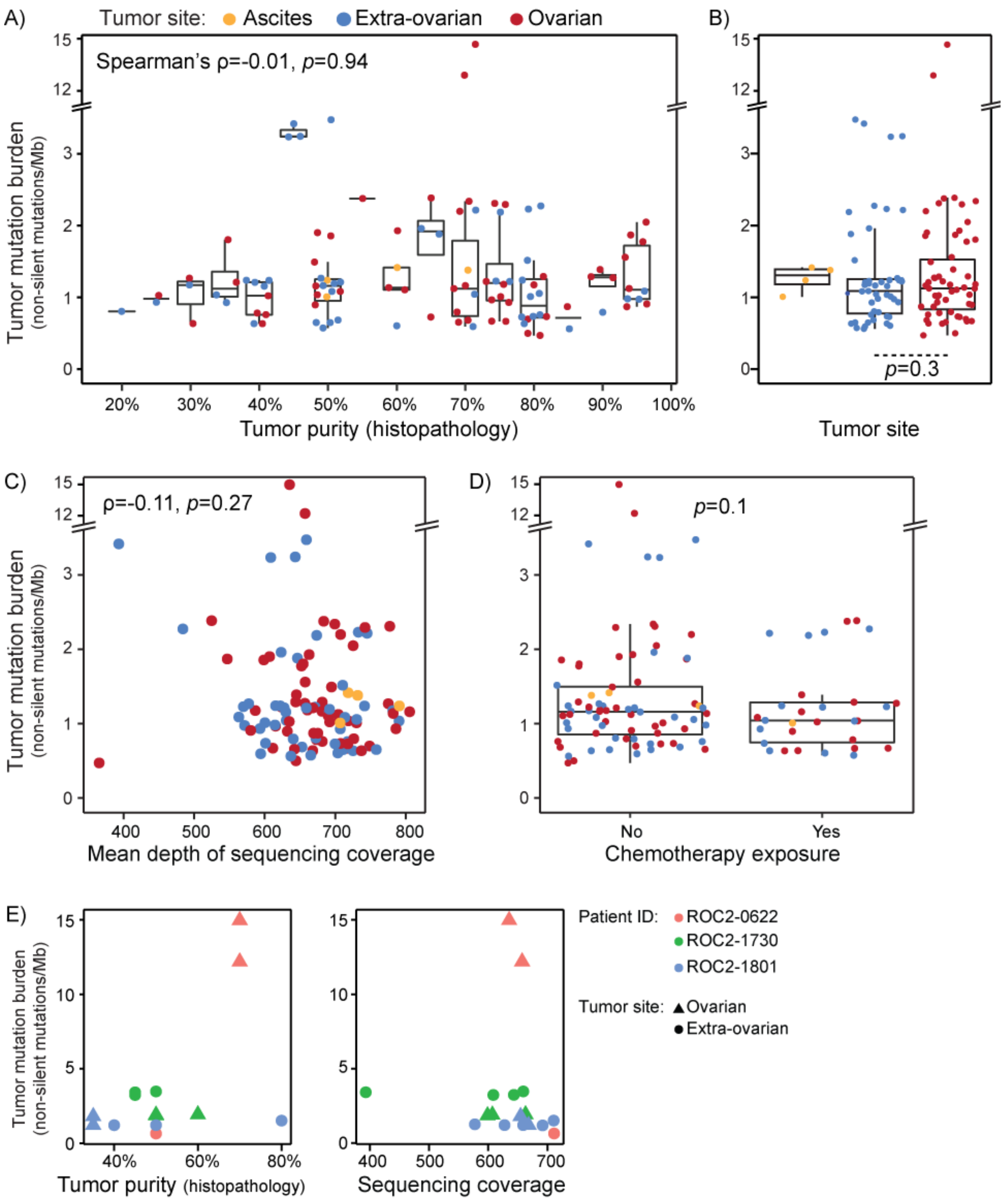

Supplemental Figure S4. Tumor mutation burden relative to sample and sequencing characteristics

The tumor mutation burden was not associated with (A) the tumor purity of the samples (categorized by pathologist with intervals of 5% based on hematoxylin and eosin stains), (B) the tumor sampling site, (C) the mean depth of sequencing coverage, or (D) previous exposure to chemotherapy. (E) This was also the case for the three patients with largest intra-patient variation in the tumor mutation burden. Color legends are the same for all plots in parts A to D. *p*-values in B and D are from Welch's t-test.

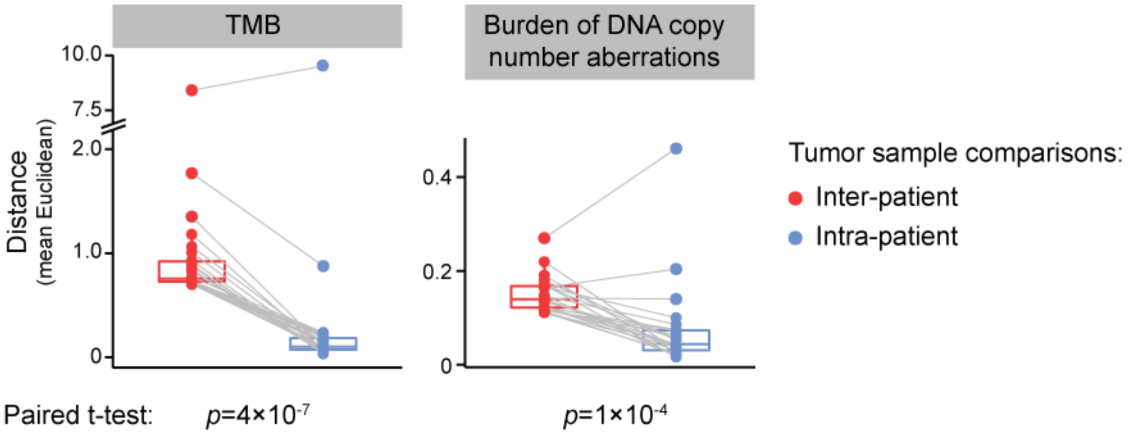

Supplemental Figure S5. Heterogeneity of the tumor mutation burden (TMB) and the burden of DNA copy number aberrations

The mean Euclidean distance of all possible pairwise inter-patient and intra-patient sample comparisons per patient (patients represented by dots) was used as estimates of heterogeneity of the TMB (left) and the burden of DNA copy number aberrations (right; based on the proportion of genes with an aberrant DNA copy number). Inter-patient heterogeneity was larger than intra-patient heterogeneity for both types of mutation burden.

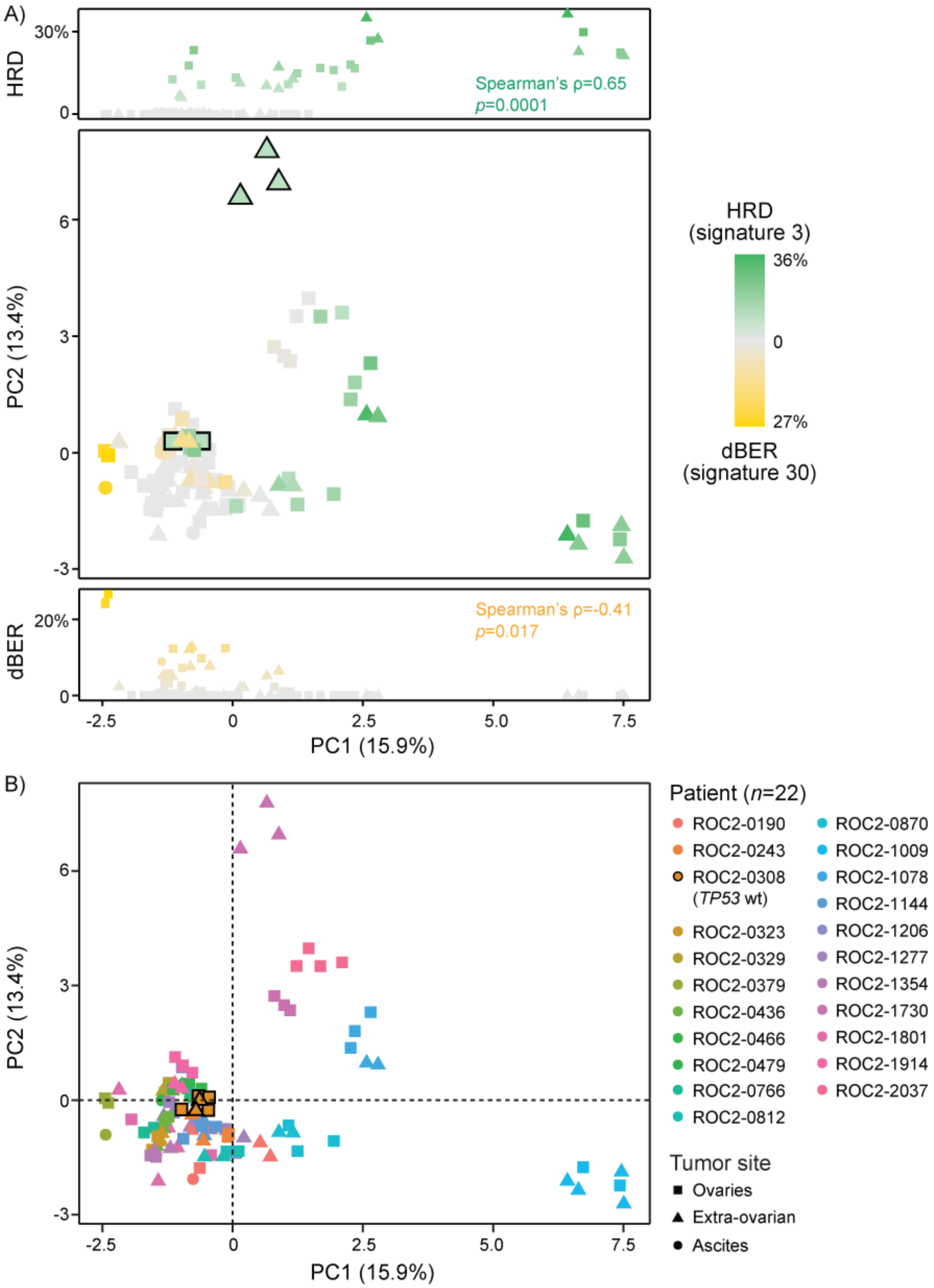

Supplemental Figure S6. Principal components analysis of mutation signatures

Principal components (PC) analysis was performed on a sample matrix representing the relative contribution of each of the COSMIC base substitution signatures to the mutations of each sample (filtered to include only signatures with a value of at least 10 in at least one sample). (A) Samples are colored according to the contribution of homologous recombination deficiency (HRD) and deficient base excision repair (dBER), and black outlines indicate samples with contributions of both.

Scatterplots indicate correlation between PC1 and HRD (top) and dBER (bottom). (B) Samples are colored according to patient, showing that samples clustered largely by patient and not by sample type. The exception was the patient with the second highest median tumor mutation burden (ROC2-1730), which had separation of the samples, indicating intra-patient heterogeneity. In addition, the patient with a hypermutated and microsatellite instability positive ovarian tumor was not included for plotting, due to the high mutation burden and deviant mutation profiles. This patient also had a high level of intra-patient mutation heterogeneity.

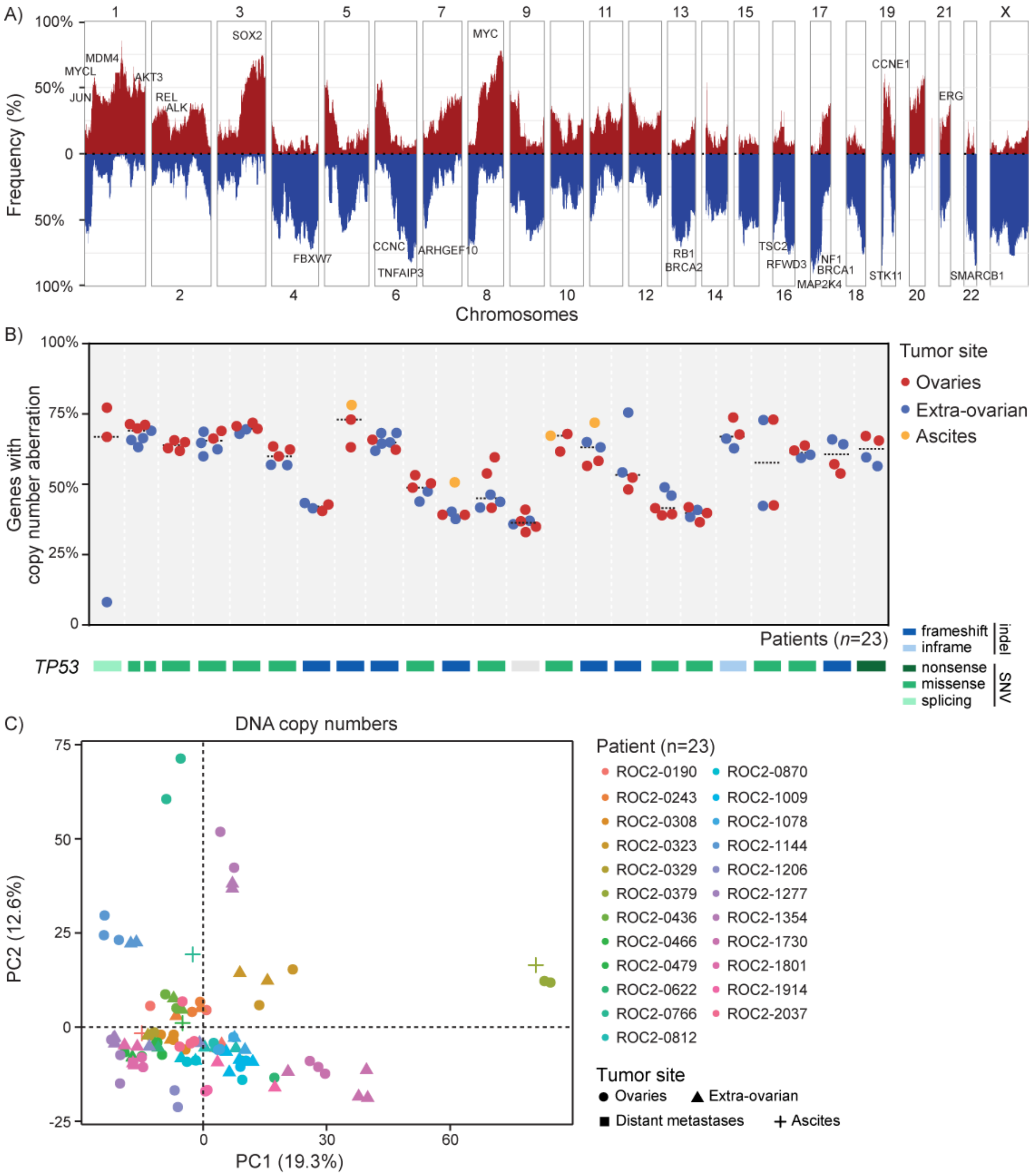

Supplemental Figure S7. DNA copy number profiles

(A) Frequency plot of DNA copy number aberrations (red: gains; blue: losses) across all cancer samples based on whole-exome sequencing. (B) The proportion of genes with a copy number aberration per sample. Samples are grouped by patient and the dashed line indicates the median proportion of aberrant genes per patient. The patients are ranked according to the tumor mutation burden of single nucleotide variants (SNVs) and insertion and deletions (indels), in the same order as in Figure 1A. (C) Principal components (PC) analysis based on DNA copy numbers of genes. The samples clustered according to the patient of origin, supporting larger inter-patient than intra-patient heterogeneity.

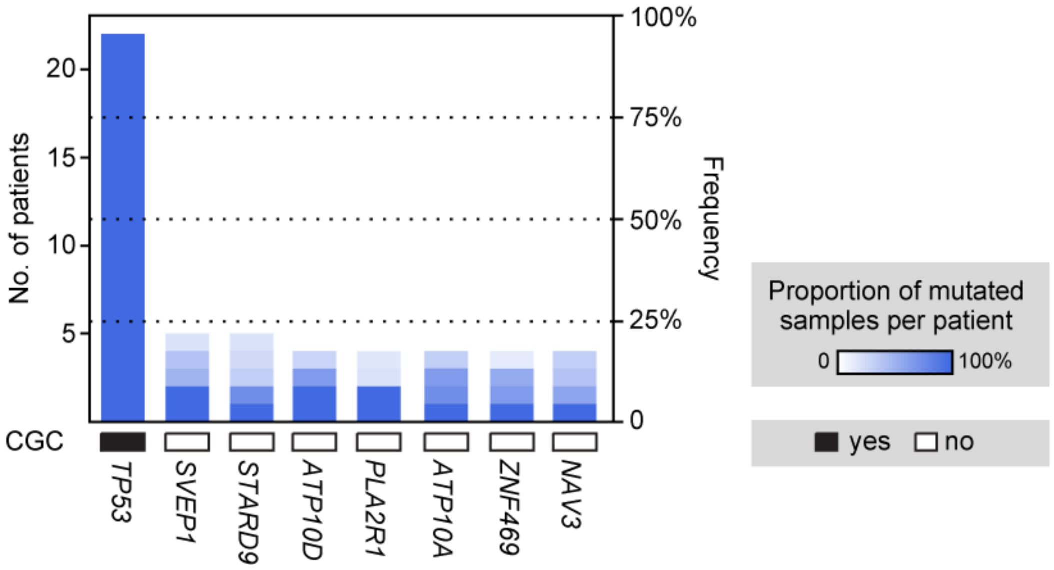

Supplemental Figure S8. Most frequently mutated genes

The mutation frequency of genes with non-silent mutations in at least one cancer sample from at least four patients. Genes indicated as "FLAGS" in Shyr et al. *BMC Med. Genomics* **2014**;7, 64 were not included. The proportion of mutated samples per patient is indicated by the blue color scale. Only *TP53* was included in the Cancer Gene Census (CGC).

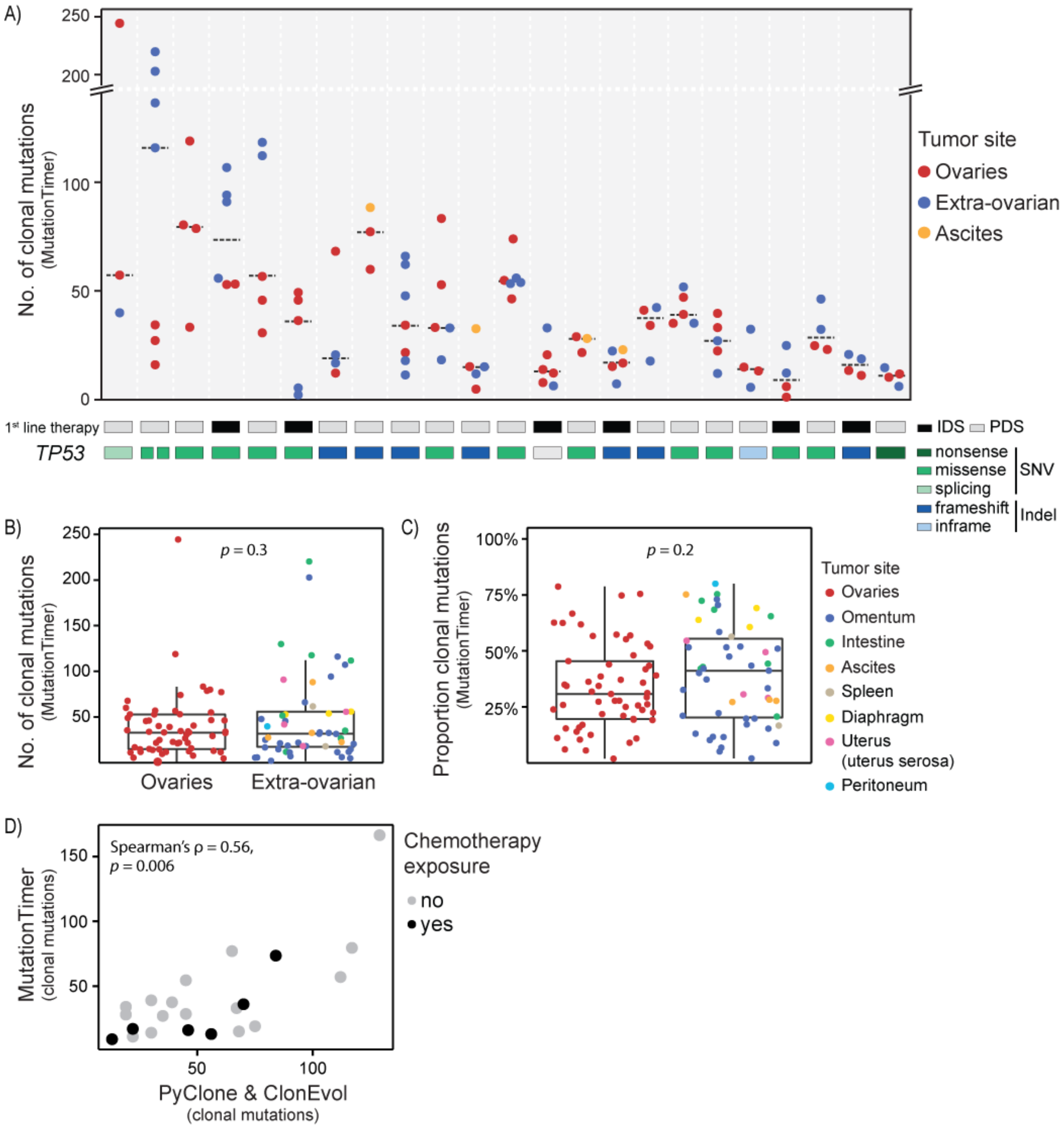

Supplemental Figure S9. Intra-patient heterogeneity of clonal mutations

(A) The burden of clonal single nucleotide variants (SNVs) and insertions and deletions (indels) per sample (clonality designated by MutationTimer). Samples are grouped by patient and colored according to tumor site. The black dashed lines indicate the median burden of clonal mutations per patient, and patients are ranked in the same order as in Figure 1A (according to a decreasing median tumor mutation burden). (B) The total number of clonal mutations and (C) the proportion of clonal mutations per sample did not differ according to tumor site. *p*-values are from Welch's t-test of ovarian versus extra-ovarian tumor samples. (D) The median burden of clonal mutations (designated by MutationTimer) per patient (vertical axis) correlated with the number of mutations in the initial clone modeled by PyClone and ClonEvol (horizontal axis).

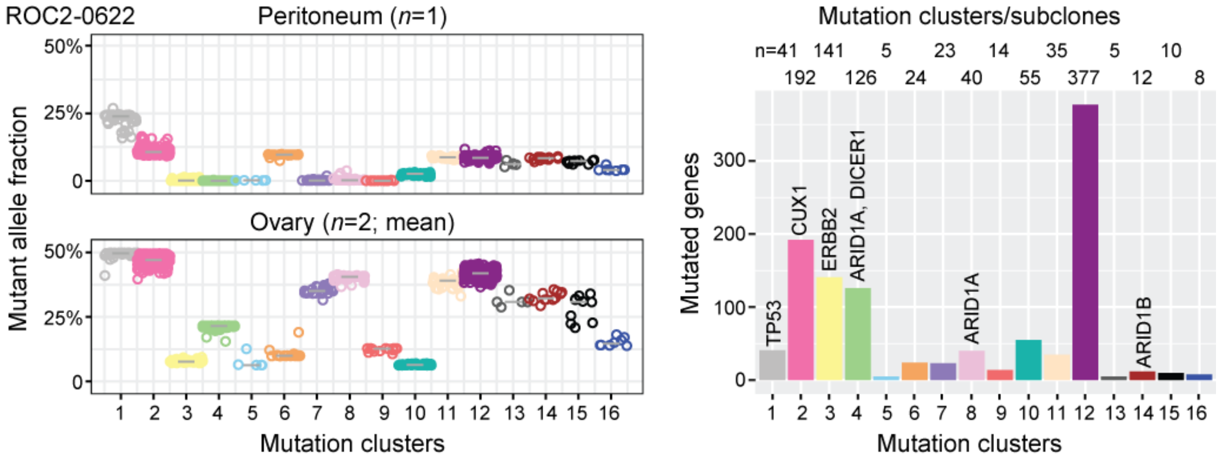

Supplemental Figure S10. Patient with hypermutated tumor and no cancer evolution model

Left plots show the mutant allele fraction of all mutations (indicated by circles) in the peritoneal sample (top) and ovarian tumor (bottom) of the patient with the highest median tumor mutation burden. Mutations are grouped according to mutation clusters (putative subclones) detected by PyClone. Bar plot to the right shows the number of mutations (non-silent and silent) per mutation cluster. Oncogenes and tumor suppressor genes with non-silent mutations are indicated. The hypermutated ovarian tumor had a large number of mutation clusters, including several that were not detected in the peritoneal sample (mutant allele fraction of zero), and the subclonal architecture of the cancer (relatedness of mutation clusters) was not possible to reconstruct. However, the cancer had a monoclonal origin, determined based on overlap of all mutations found in the peritoneal sample with mutations of the ovarian tumor. Deficient DNA mismatch repair likely developed in the ovarian tumor after the last dissemination step between the two sites, resulting in tumor heterogeneity and a large number of additional mutations in the ovarian tumor.

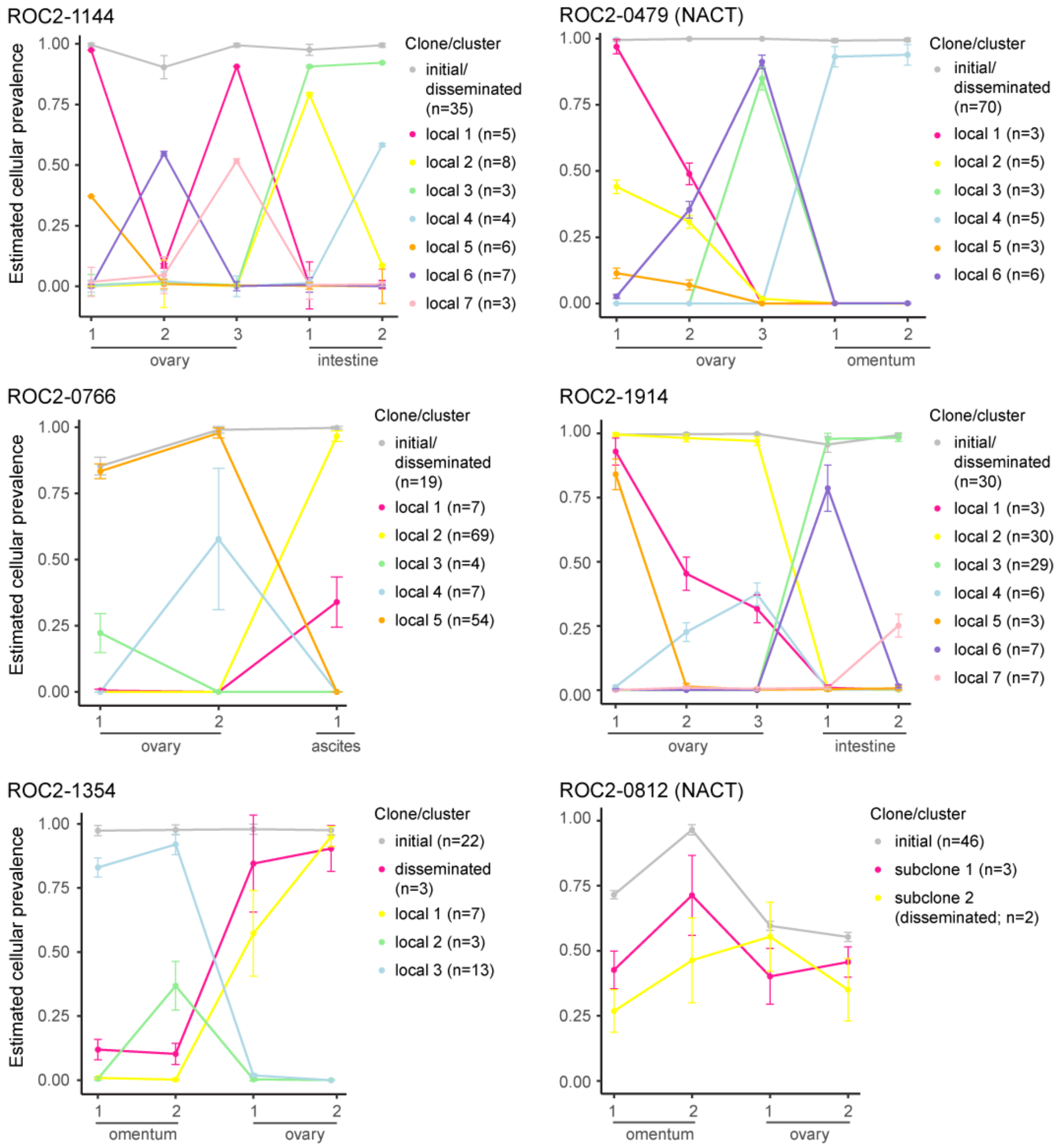

Supplemental Figure S11. Estimated cellular prevalence of cancer clones in HGSCs with monoclonal dissemination

Mutation clusters/clones and their cellular prevalence were estimated by PyClone based on mutant allele fractions of SNVs and indels (numbers of mutations per clone indicated in parenthesis), and adjusted for allele-specific copy numbers and the tumor purity of each sample. Samples are plotted on the horizontal axis and ordered by tumor site. Error bars indicate the mean standard deviation of the estimated cellular prevalence. Mutation clusters/clones with an estimated cellular prevalence of approximately one across all samples were designated as the initial clone (grey). Clones present across tumor sites at a lower and/or varying cellular prevalence were designated as disseminated, and clones present in a single tumor site as local. Standard deviations were generally largest for the local subclones. Large standard deviations of the disseminated subclone of ROC2-1354 (pink) indicate that the subclone might not be present in all cancer cells of the ovarian tumor, opening up the

possibility that the initial clone (grey) also disseminated and that the cancer had monophyletic polyclonal dissemination. ROC2-0812 had no clones with a mean estimated cellular prevalence of one, indicating uncertainty of this cancer model in general. A cluster with only two mutations (yellow; below the filtering threshold of three) was kept for modeling, but this did not have an impact on the modeled dissemination mode. The cancer was exposed to neoadjuvant chemotherapy (NACT).

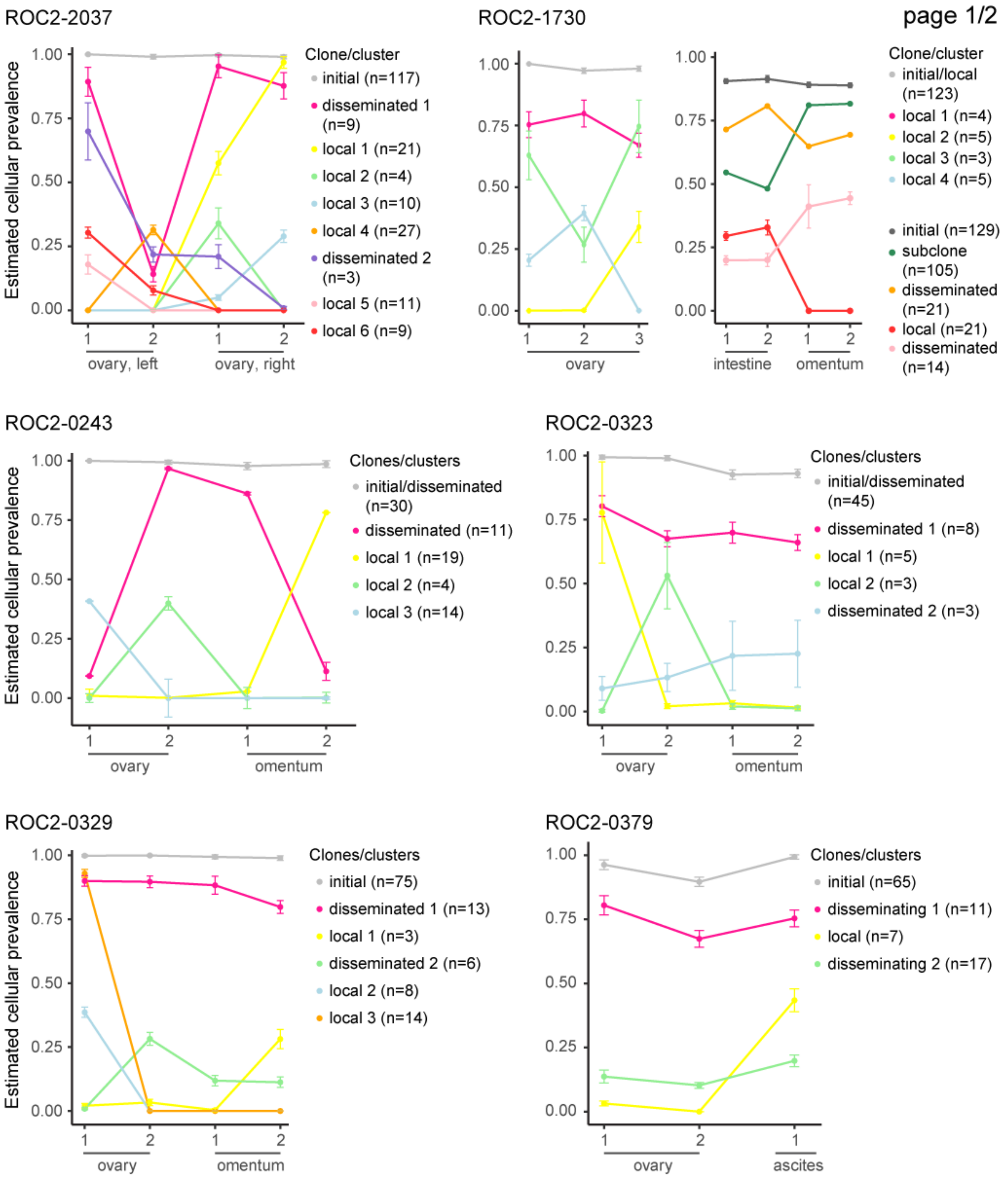
ROC2-2037
Estimated cellular prevalence
1.00
0.75
0.50
0.25
0.00
1
2
1
2
ovary, left
ovary, right
Clone/cluster
initial (n=117)
disseminated 1 (n=9)
local 1 (n=21)
local 2 (n=4)
local 3 (n=10)
local 4 (n=27)
disseminated 2 (n=3)
local 5 (n=11)
local 6 (n=9)
ROC2-1730
page 1/2
1.00
0.75
0.50
0.25
0.00
1
2
3
ovary
1.00
0.75
0.50
0.25
0.00
1
2
1
2
intestine
omentum
Clone/cluster
initial/local (n=123)
local 1 (n=4)
local 2 (n=5)
local 3 (n=3)
local 4 (n=5)
initial (n=129)
subclone (n=105)
disseminated (n=21)
local (n=21)
disseminated (n=14)
ROC2-0243
Estimated cellular prevalence
1.00
0.75
0.50
0.25
0.00
1
2
1
2
ovary
omentum
Clones/clusters
initial/disseminated (n=30)
disseminated (n=11)
local 1 (n=19)
local 2 (n=4)
local 3 (n=14)
ROC2-0323
1.00
0.75
0.50
0.25
0.00
1
2
1
2
ovary
omentum
Clones/clusters
initial/disseminated (n=45)
disseminated 1 (n=8)
local 1 (n=5)
local 2 (n=3)
disseminated 2 (n=3)
ROC2-0329
Estimated cellular prevalence
1.00
0.75
0.50
0.25
0.00
1
2
1
2
ovary
omentum
Clones/clusters
initial (n=75)
disseminated 1 (n=13)
local 1 (n=3)
disseminated 2 (n=6)
local 2 (n=8)
local 3 (n=14)
ROC2-0379
1.00
0.75
0.50
0.25
0.00
1
2
1
ovary
ascites
Clones/clusters
initial (n=65)
disseminating 1 (n=11)
local (n=7)
disseminating 2 (n=17)

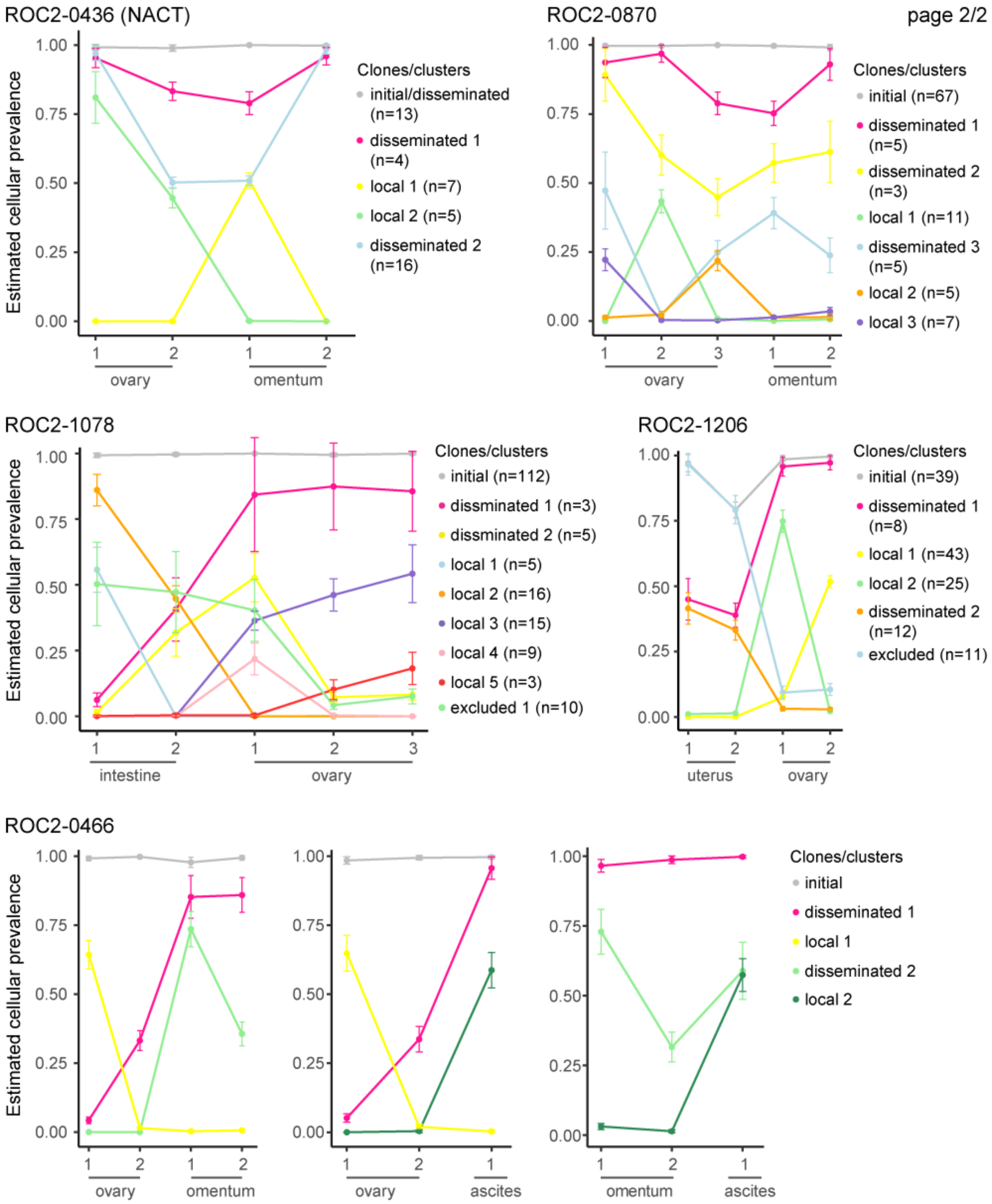

Supplemental Figure S12. Estimated cellular prevalence of cancer clones in HGSCs with monophyletic polyclonal seeding (seeding of multiple subclones of a linear evolutionary lineage)

Mutation clusters/clones and their cellular prevalence were estimated by PyClone based on mutant allele fractions of SNVs and indels (numbers of mutations per clone indicated in parenthesis), and adjusted for allele-specific copy numbers and the tumor purity of each sample. Samples are plotted on the horizontal axis and ordered by tumor site. Error bars indicate the mean standard deviation of the estimated cellular prevalence. Mutation clusters/clones with an estimated cellular prevalence of approximately one across all samples were designated as the initial clone (grey). Clones present

across tumor sites at a lower and/or varying cellular prevalence were designated as disseminated, and clones present in a single tumor site as local. ROC2-1730 had a polyclonal cancer origin and no common mutations/clones between the ovarian and extra-ovarian tumors. The green and orange subclones of the extra-ovarian cancer have been manually curated and represent a collection of two and three mutation clusters, respectively, which had similar mean estimated cellular prevalence but large within-cluster standard deviations. There is uncertainty in the relationship of the green and orange subclones. A branched evolutionary relationship (as indicated by the relative size changes at the two tumor sites) would mean polyphyletic dissemination of the orange and pink clones, but the estimated cellular prevalence is too large for the two disseminated clones to co-exist as branched subclones. For ROC2-0323, the relationship (linear or branched) of the pink and blue subclones is uncertain. Both subclones have disseminated and both monophyletic and polyphyletic dissemination modes are therefore possible. One mutation cluster from each of ROC2-1078 and ROC2-1206 were excluded from further analyses (indicated in the respective plots) because subclonal architecture models were not possible to generate with these clusters included. This did not impact the conclusion of polyclonal dissemination of the cancers.

ROC2-1801

page 1/2

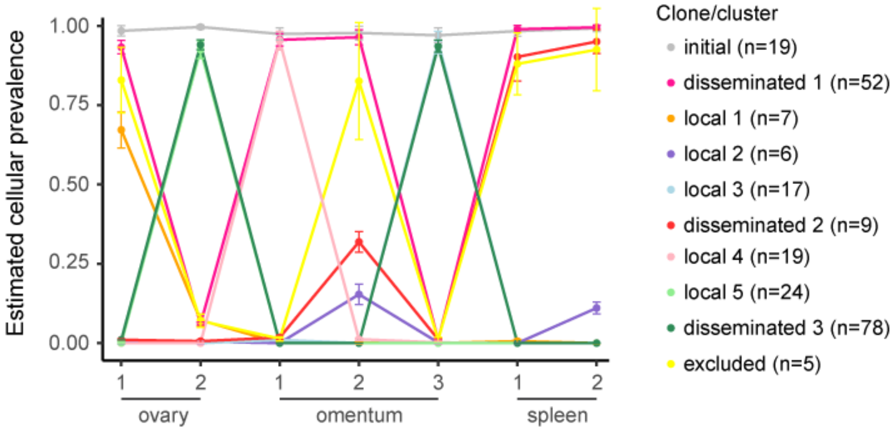

ROC2-0190 (NACT)

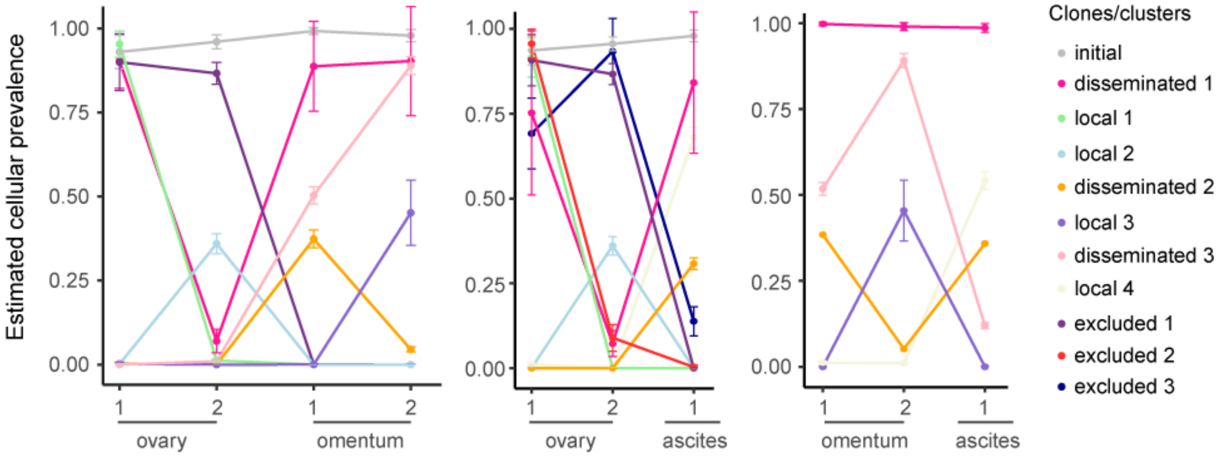

ROC2-1277

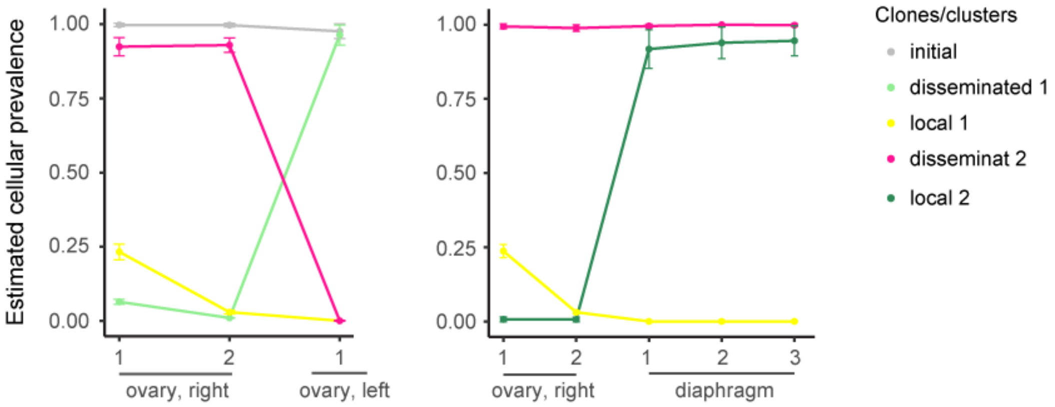

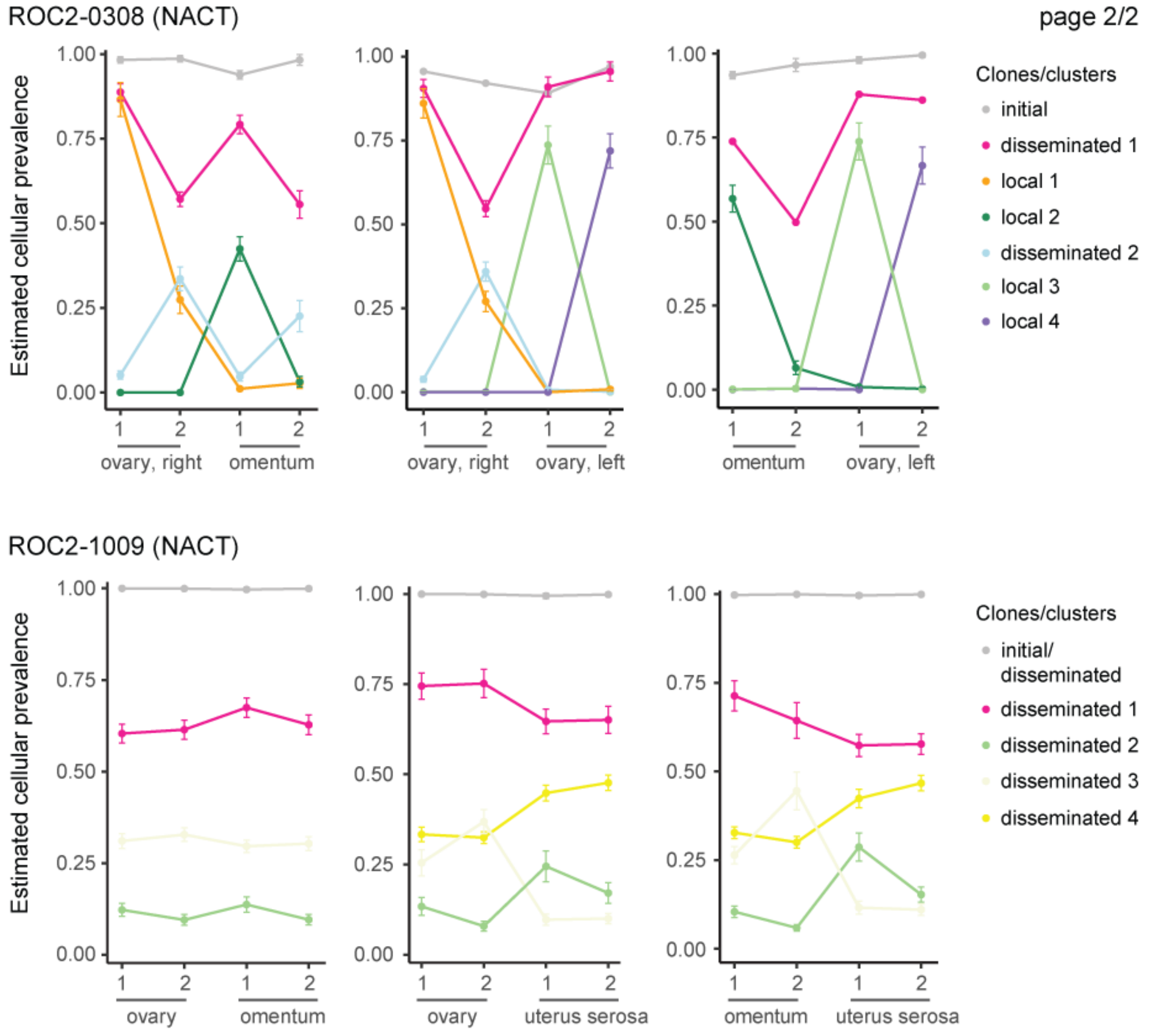

Supplemental Figure S13. Estimated cellular prevalence of cancer clones in HGSCs with polyphyletic seeding (seeding of multiple subclones of a branched evolutionary lineage)

Mutation clusters/clones and their cellular prevalence were estimated by PyClone based on mutant allele fractions of SNVs and indels (numbers of mutations per clone indicated in parenthesis), and adjusted for allele-specific copy numbers and the tumor purity of each sample. Samples are plotted on the horizontal axis and ordered by tumor site. Error bars indicate the mean standard deviation of the estimated cellular prevalence. Mutation clusters/clones with an estimated cellular prevalence of approximately one across all samples were designated as the initial clone (grey). Clones present across tumor sites at a lower and/or varying cellular prevalence were designated as disseminated, and clones present in a single tumor site as local. Selected subclones/mutation clusters were excluded or merged in three of the five cancers, but this did not impact the conclusion of polyphyletic dissemination. In ROC2-1801, one cluster of five mutations present across tumor sites was excluded as indicated. In ROC2-0190, the orange subclone is a merger of three subclones with similar cellular prevalence and large variation within each cluster in the comparison of omentum and ascites. One and three subclones were removed from the comparisons of ovary with either the omentum or ascites, respectively. In ROC2-0308, two mutation clusters were merged for comparisons including the left ovarian tumor.

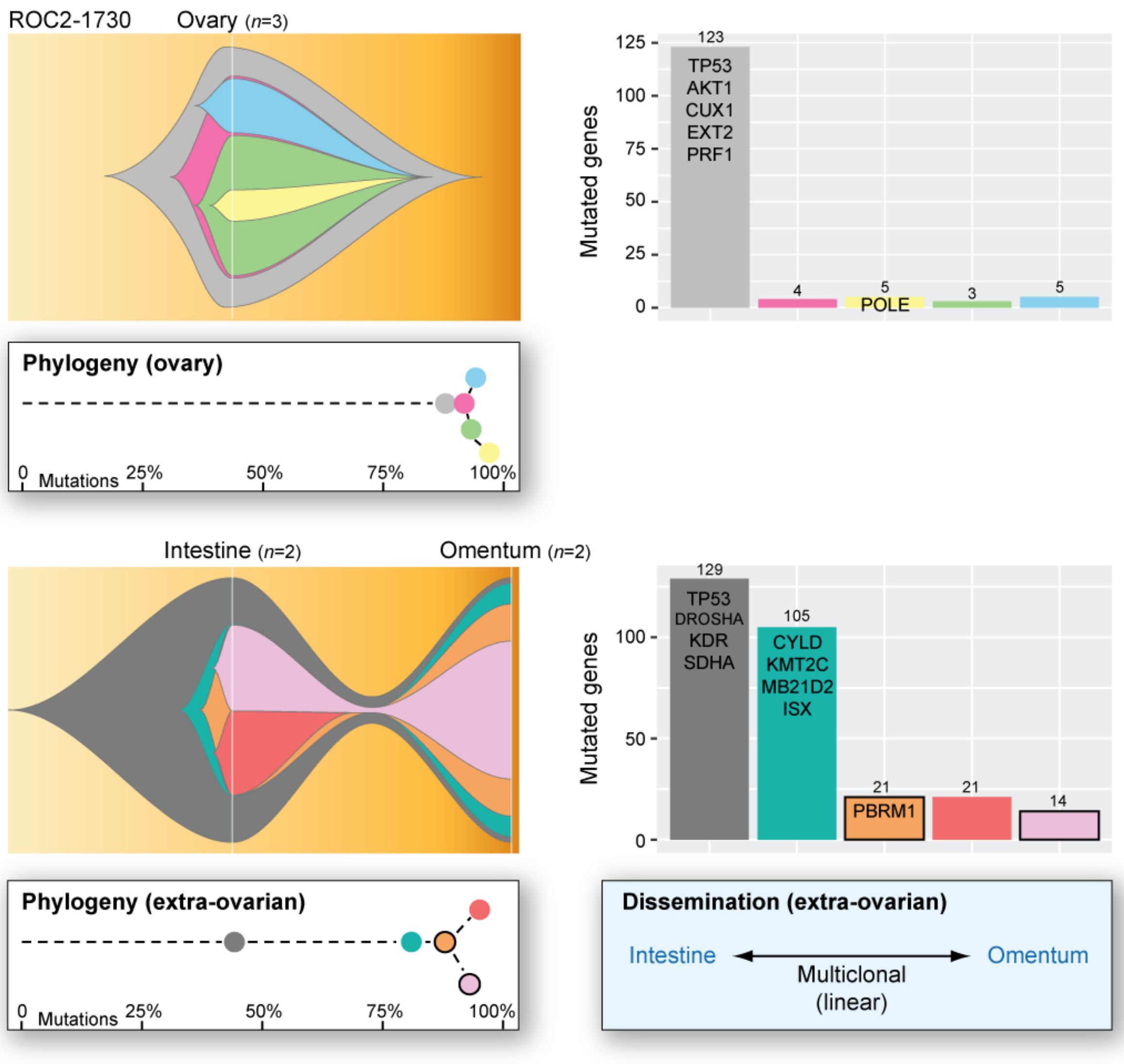

Supplemental Figure S14. Patient with polyclonal cancer origin

Fish plots illustrating the subclonal architectures and evolution of the ovarian tumor (top) and extra-ovarian tumors (bottom) of the patient with a polyclonal cancer origin (no common mutations between the ovarian and extra-ovarian cancers). Missense *TP53* mutations were found in the initial clone of both cancers, but at different loci. The ovarian tumor was subclonal and had not disseminated. The extra-ovarian cancer showed dissemination of two related subclones (established by linear evolution) between the intestine and the omentum, with an undetermined direction of seeding (possibly bidirectional). Notably, there was ambiguity in the relatedness of the green and orange subclones in the extra-ovarian cancer (Supplemental Figure S12), and the fish plot illustrates the most likely model and a linear relationship. The bar plots to the right show the number of mutations (non-silent and silent) per subclone, and oncogenes and tumor suppressor genes with non-silent mutations are indicated. The white boxes show a schematic of the phylogeny of each cancer, illustrated as a horizontal dot plot of the relative evolutionary timing of development of each clone (proportion of mutations in the clone relative to the total number of mutations in the cancer model). Black outlines mark disseminated clones, which are present in samples from more than one tumor site.

A)

page 1/2

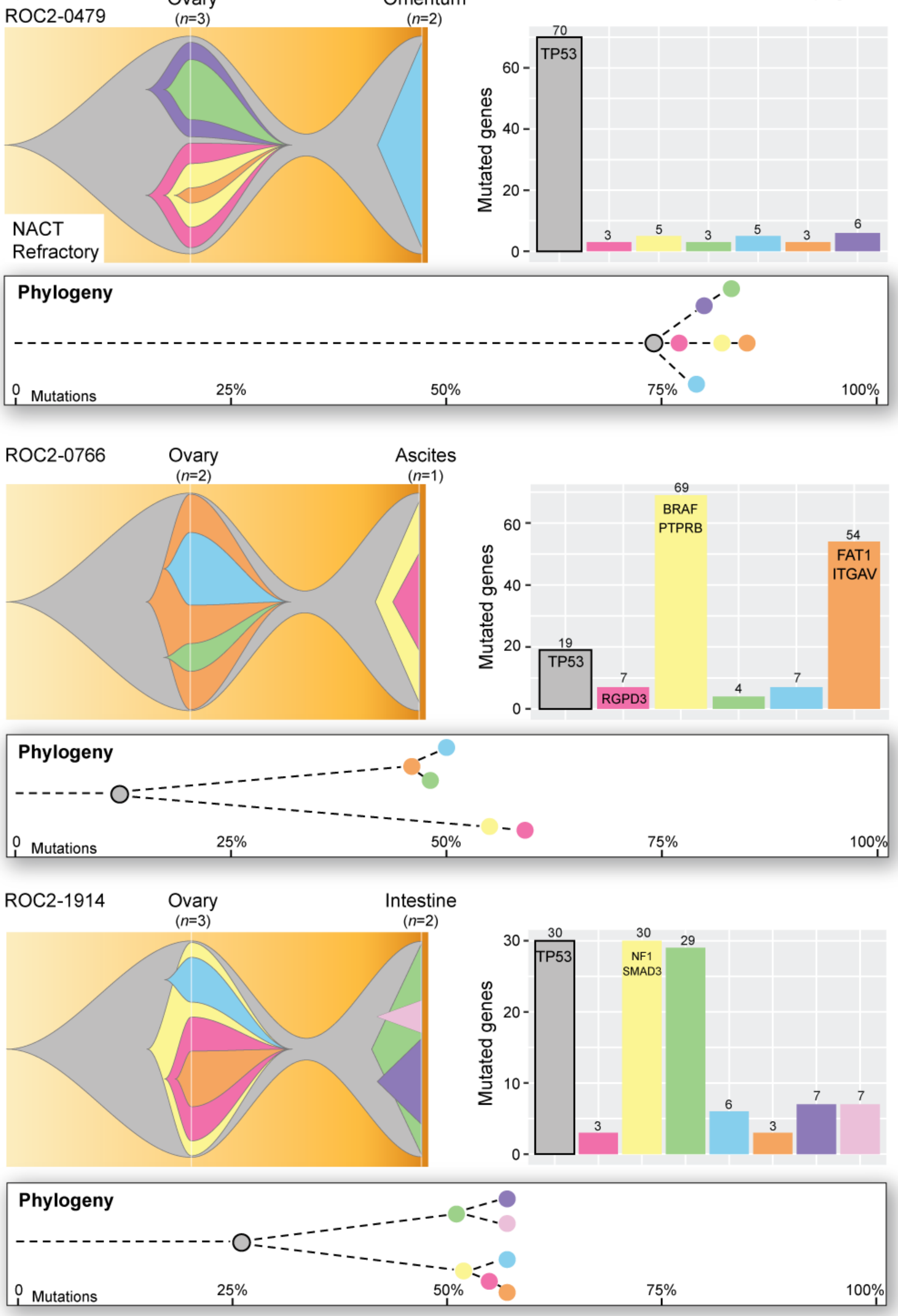
ROC2-0479
Ovary
(n=3)
Omentum
(n=2)
NACT
Refractory
Mutated genes
70
TP53
3
5
3
5
3
6
60
40
20
0
Phylogeny
0
Mutations
25%
50%
75%
100%
ROC2-0766
Ovary
(n=2)
Ascites
(n=1)
Mutated genes
19
TP53
7
RGPD3
69
BRAF
PTPRB
4
7
54
FAT1
ITGAV
60
40
20
0
Phylogeny
0
Mutations
25%
50%
75%
100%
ROC2-1914
Ovary
(n=3)
Intestine
(n=2)
Mutated genes
30
TP53
3
30
NF1
SMAD3
29
6
3
7
7
30
20
10
0
Phylogeny
0
Mutations
25%
50%
75%
100%

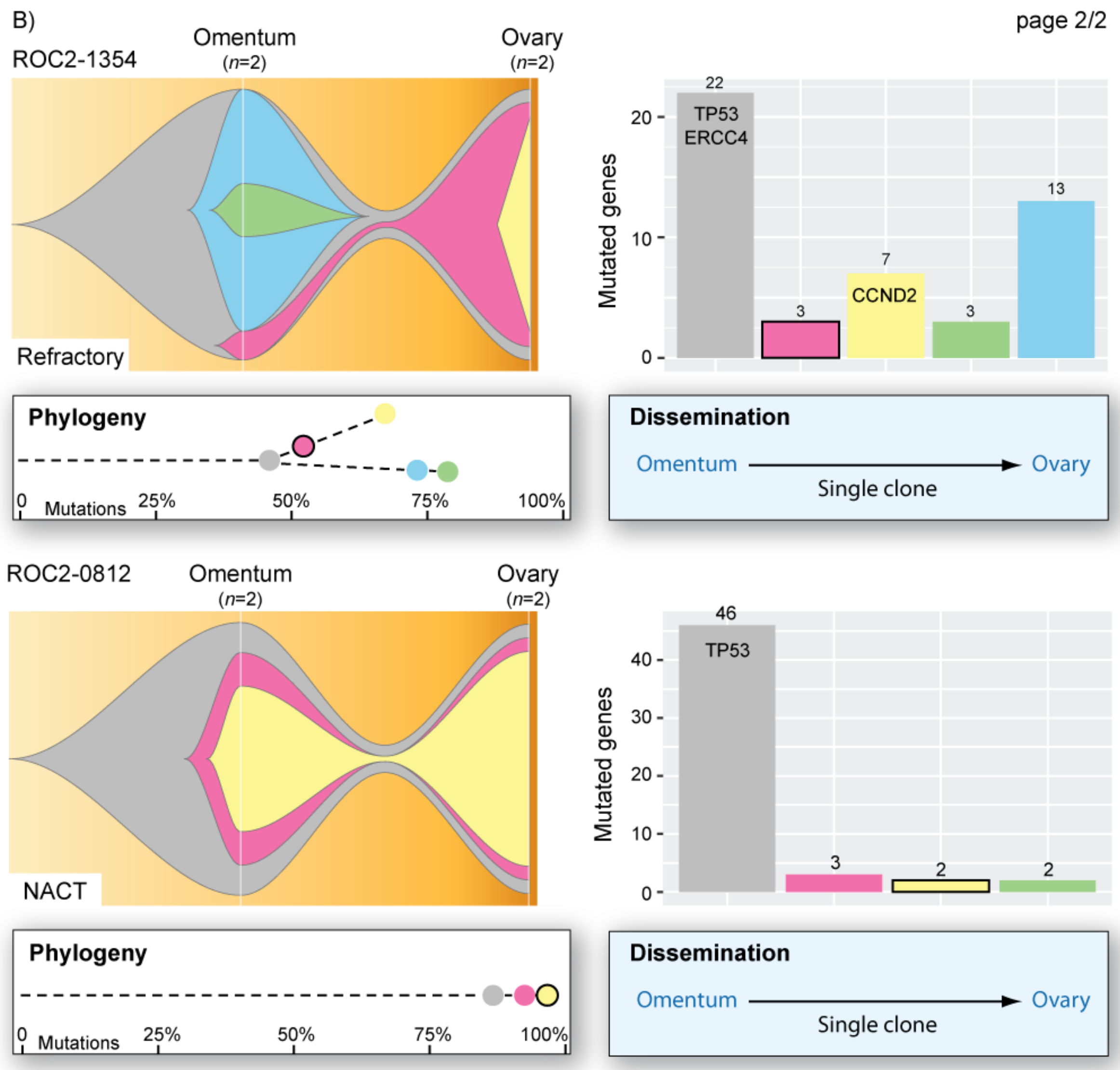

Supplemental Figure S15. Cancers with monoclonal dissemination

Fish plots of the clonal architecture, bar plots of the number of mutations per subclone, and schematics of the phylogeny indicating the relative timing of establishment of each subclone in the cancers that disseminated in a single step. The subclones have the same color in all plots per patient, and the initial clone in is grey. The single clone that disseminated is indicated by a black outline. Cancers exposed to chemotherapy prior to sampling (neoadjuvant chemotherapy, NACT) and/or were refractory to first line treatment are marked. (A) Four cancers had seeding of the initial clone (the three cancers illustrated here plus the cancer shown in Figure 3A). (B) Two cancers had seeding of a subclone from the extra-ovarian tumor site (the omentum in both cases) to the ovary. The direction of seeding was determined based on the cellular prevalence of the disseminated subclone at the separate tumor sites, and the site with the lowest prevalence was considered to be the seeding site. ROC2-1354 has a known origin in the fallopian tube (based on histopathology of diagnostic specimens). Uncertainties in the models of ROC2-1354 and ROC2-0812 are described in Supplemental Figure S11 and the best model fits are illustrated here.

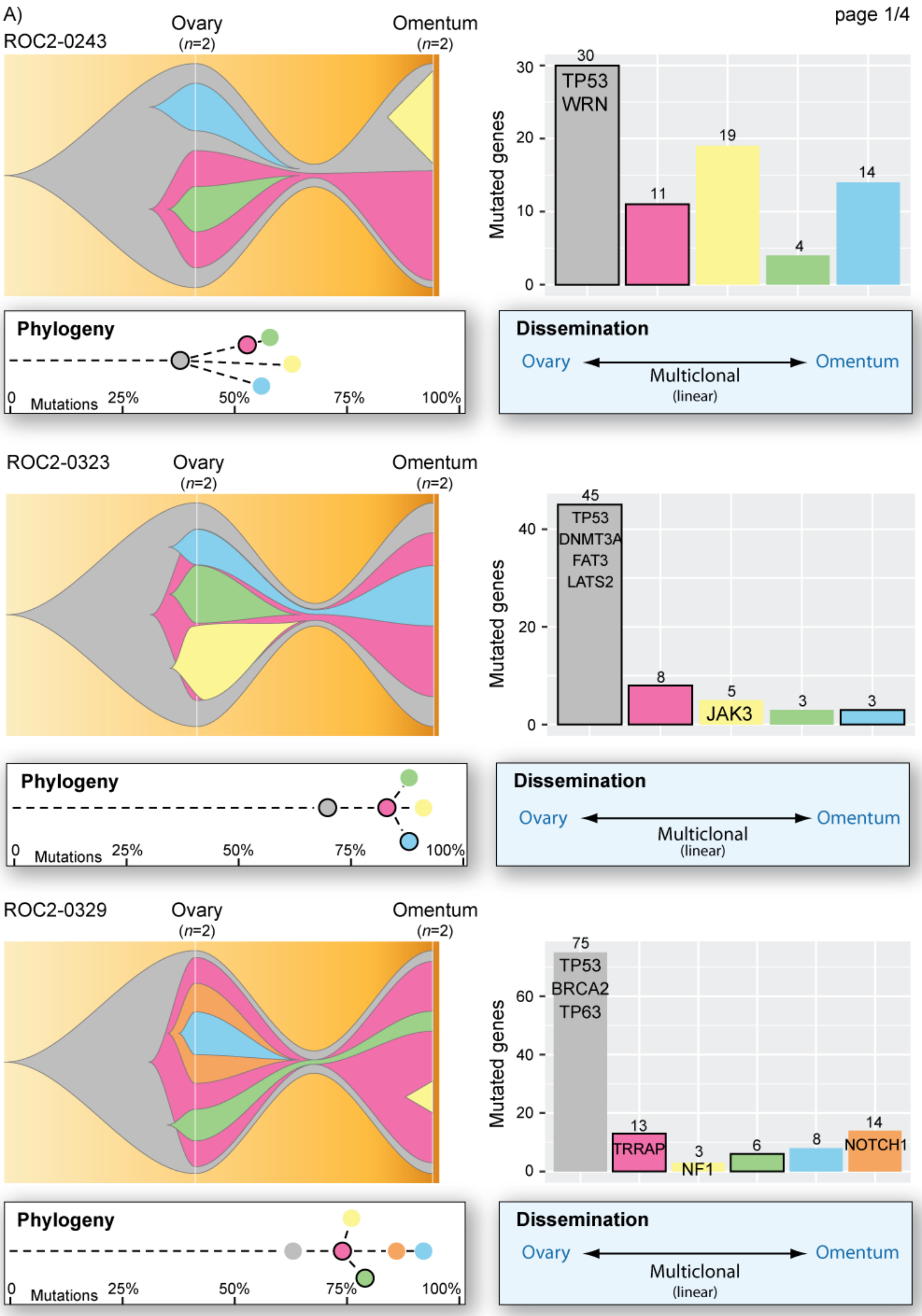
A)
page 1/4
ROC2-0243
Ovary
(n=2)
Omentum
(n=2)
Mutated genes
30
TP53
WRN
11
19
4
14
Phylogeny
0
Mutations
25%
50%
75%
100%
Dissemination
Ovary
Omentum
Multiclonal
(linear)
ROC2-0323
Ovary
(n=2)
Omentum
(n=2)
Mutated genes
45
TP53
DNMT3A
FAT3
LATS2
8
5
JAK3
3
3
Phylogeny
0
Mutations
25%
50%
75%
100%
Dissemination
Ovary
Omentum
Multiclonal
(linear)
ROC2-0329
Ovary
(n=2)
Omentum
(n=2)
Mutated genes
75
TP53
BRCA2
TP63
13
TRRAP
3
NF1
6
8
14
NOTCH1
Phylogeny
0
Mutations
25%
50%
75%
100%
Dissemination
Ovary
Omentum
Multiclonal
(linear)

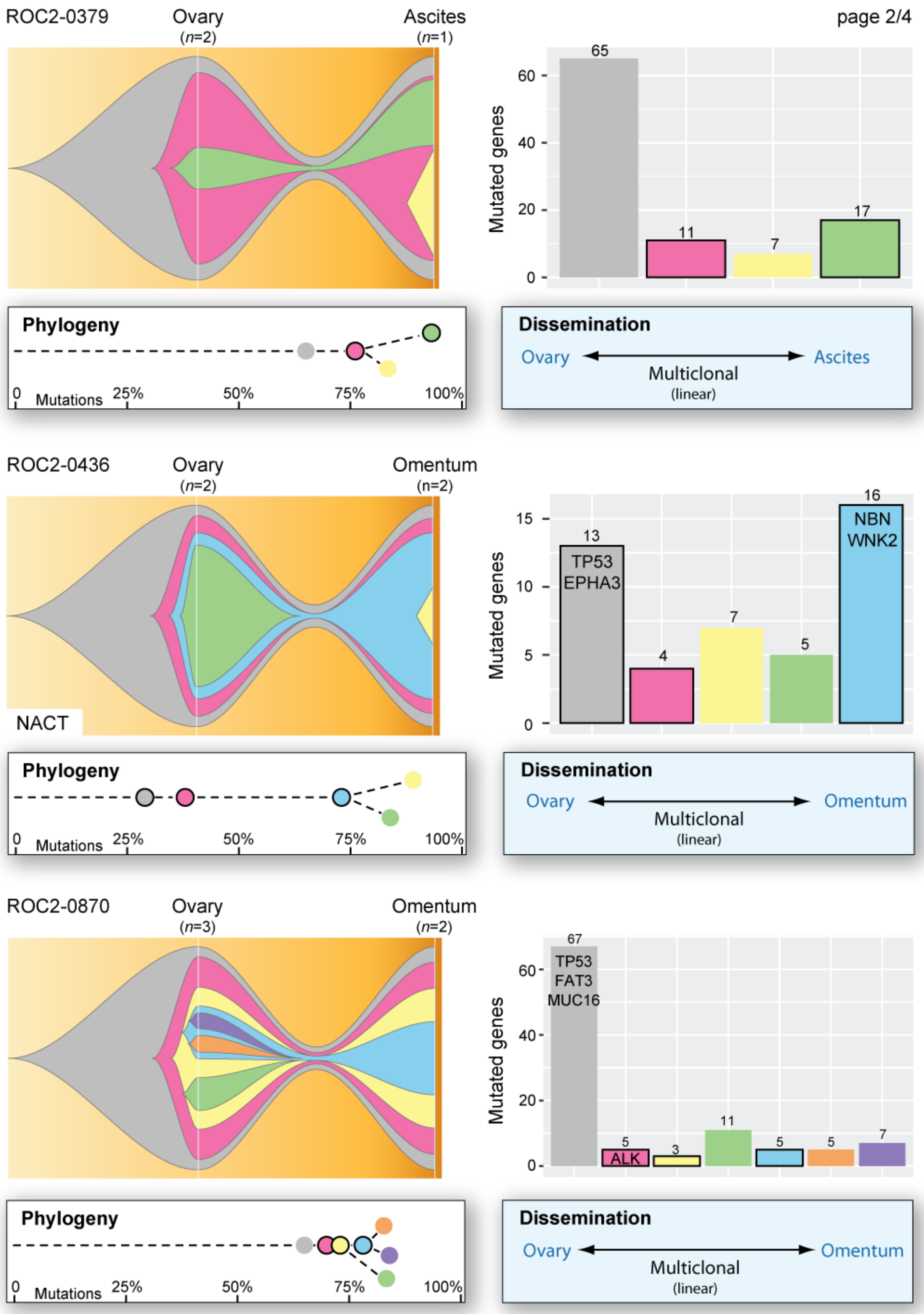
ROC2-0379
Ovary
(n=2)
Ascites
(n=1)
page 2/4
Mutated genes
65
11
7
17
60
40
20
0
Phylogeny
0
Mutations
25%
50%
75%
100%
Dissemination
Ovary
Ascites
Multiclonal
(linear)
ROC2-0436
Ovary
(n=2)
Omentum
(n=2)
NACT
Mutated genes
15
10
5
0
13
TP53
EPHA3
4
7
5
16
NBN
WNK2
Phylogeny
0
Mutations
25%
50%
75%
100%
Dissemination
Ovary
Omentum
Multiclonal
(linear)
ROC2-0870
Ovary
(n=3)
Omentum
(n=2)
Mutated genes
60
40
20
0
67
TP53
FAT3
MUC16
5
ALK
3
11
5
5
7
Phylogeny
0
Mutations
25%
50%
75%
100%
Dissemination
Ovary
Omentum
Multiclonal
(linear)

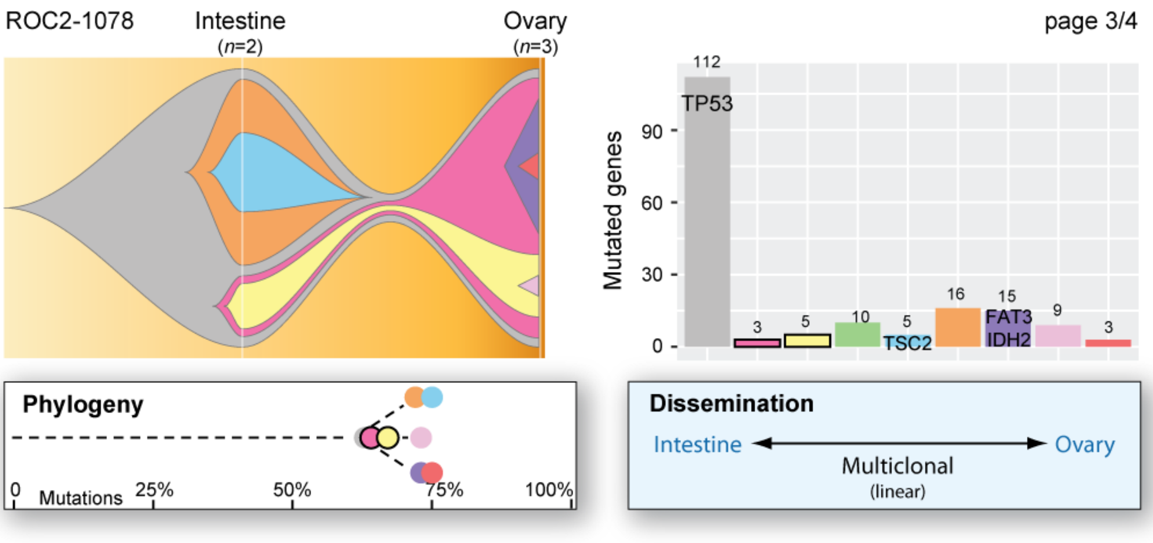
ROC2-1078
Intestine
(n=2)
Ovary
(n=3)
112
TP53
90
60
30
0
Mutated genes
3
5
10
5
TSC2
16
15
FAT3
IDH2
9
3
Phylogeny
0
Mutations
25%
50%
75%
100%
Dissemination
Intestine
Ovary
Multiclonal
(linear)

B)

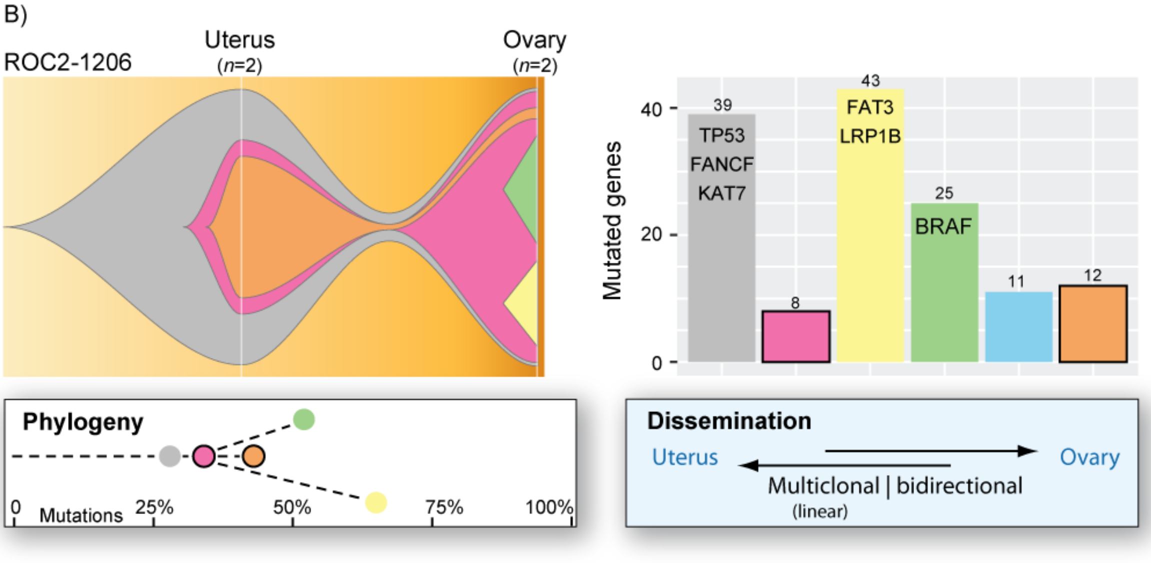
ROC2-1206
Uterus
(n=2)
Ovary
(n=2)
40
20
0
Mutated genes
39
TP53
FANCF
KAT7
8
43
FAT3
LRP1B
25
BRAF
11
12
Phylogeny
0
Mutations
25%
50%
75%
100%
Dissemination
Uterus
Ovary
Multiclonal | bidirectional
(linear)

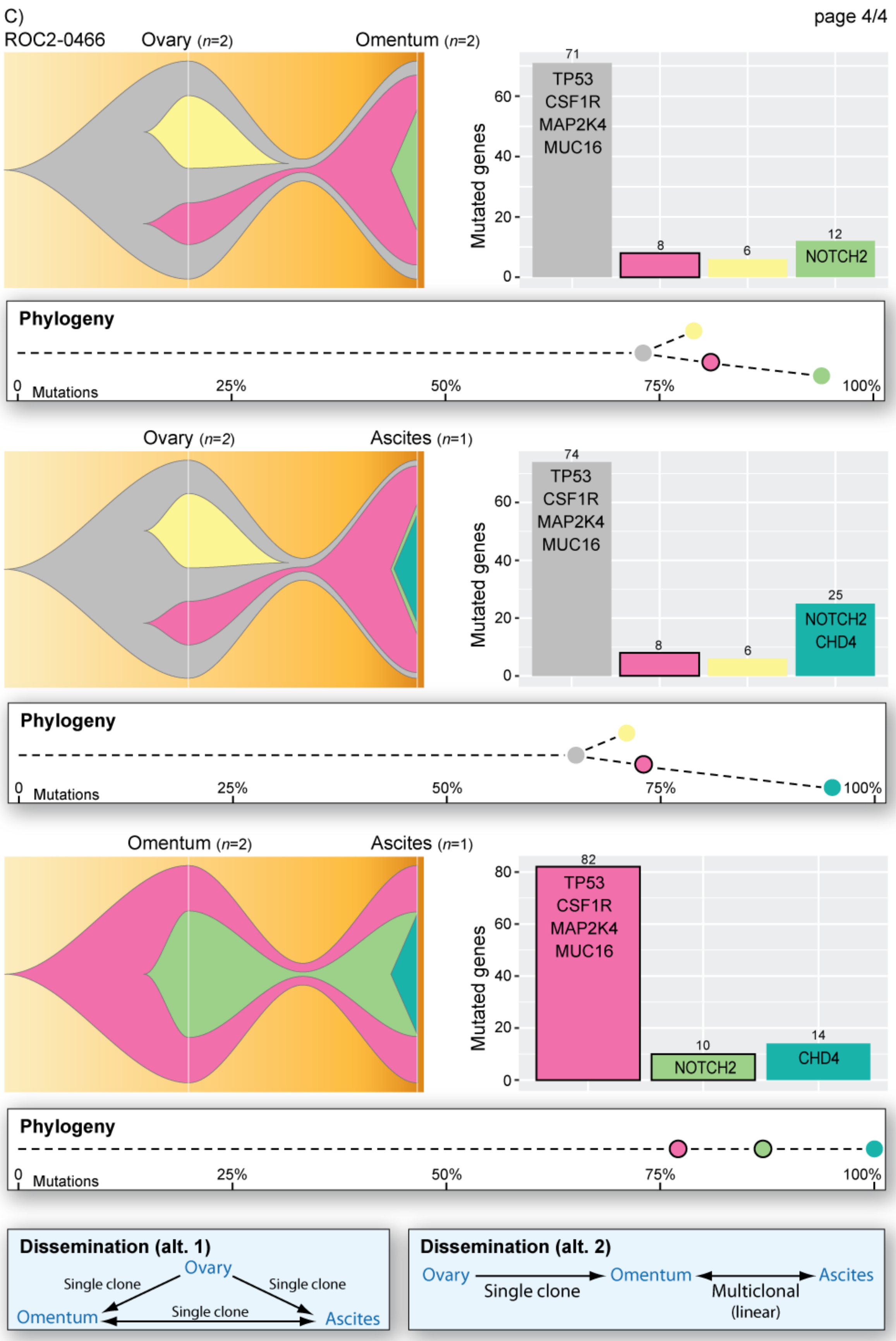
C)
page 4/4
ROC2-0466
Ovary (n=2)
Omentum (n=2)
Mutated genes
71
TP53
CSF1R
MAP2K4
MUC16
8
6
12
NOTCH2
Phylogeny
0 Mutations
25%
50%
75%
100%
Ovary (n=2)
Ascites (n=1)
Mutated genes
74
TP53
CSF1R
MAP2K4
MUC16
8
6
25
NOTCH2
CHD4
Phylogeny
0 Mutations
25%
50%
75%
100%
Omentum (n=2)
Ascites (n=1)
Mutated genes
82
TP53
CSF1R
MAP2K4
MUC16
10
NOTCH2
14
CHD4
Phylogeny
0 Mutations
25%
50%
75%
100%
Dissemination (alt. 1)
Ovary
Single clone
Single clone
Omentum
Single clone
Ascites
Dissemination (alt. 2)
Ovary
Single clone
Omentum
Multiclonal
(linear)
Ascites

Supplemental Figure S16. Cancers with monophyletic polyclonal seeding (seeding of multiple subclones of a linear evolutionary lineage)

Fish plots of the clonal architecture, bar plots of the number of mutations per subclone, and schematics of the phylogeny indicating the relative timing of establishment of each subclone in cancers with dissemination of multiple (at least two) related subclones resulting from linear evolution. There were totally 11 (50%) cancers that showed this mode of dissemination, nine of which are illustrated in this figure. The remaining two are shown in Figure 3B and in Supplemental Figure S14 (the extra-ovarian cancer from the patient with polyclonal cancers). (A) Cancers (*n* = 7) with unknown direction of seeding (indicated by bidirectional arrows). Notably, the relationship of two of the disseminated subclones in ROC2-0323 (pink and blue) is uncertain (Supplemental Figure S12). ROC2-0870 had multiple possible models of relatedness among the blue, purple, orange, and green subclones. The cancer exposed to chemotherapy prior to sampling is marked (neoadjuvant chemotherapy, NACT). (B) Cancers (*n* = 1; the second is shown in Figure 3B) with bidirectional seeding of subclones between the two tumor sites, first the pink subclone from the uterus to the ovary, followed by seeding of the orange subclone in the opposite direction. (C) Cancer with three tumor sites (all three possible pairwise comparisons are shown). The model indicates that the ovary was the first tumor site, with dissemination of the pink subclone to the omentum and the ascites (alternative 1). Alternatively, the pink subclone disseminated from the omentum to the ascites, with no dissemination between the ovary and ascites (alternative 2). Dissemination of the green subclone between the omentum and ascites has an unknown direction.

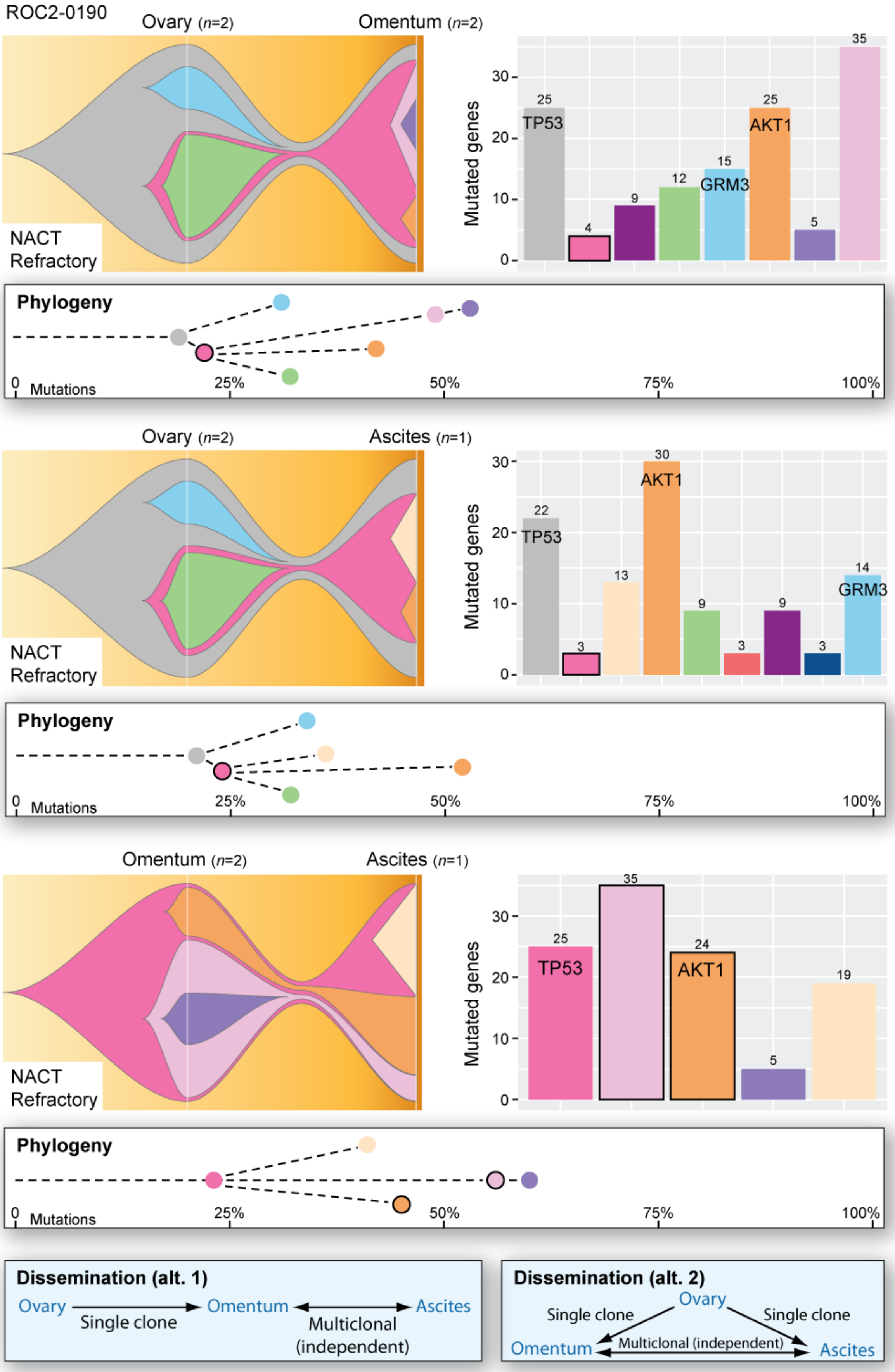

ROC2-0190
Ovary (n=2)
Omentum (n=2)
NACT Refractory
Mutated genes
TP53
AKT1
GRM3
Phylogeny
Mutations
25%
50%
75%
100%
Ovary (n=2)
Ascites (n=1)
NACT Refractory
Omentum (n=2)
Ascites (n=1)
NACT Refractory
Dissemination (alt. 1)
Ovary
Single clone
Omentum
Ascites
Multiclonal (independent)
Dissemination (alt. 2)
Ovary
Single clone
Single clone
Omentum
Multiclonal (independent)
Ascites

Supplemental Figure S17. Cancer with polyphyletic seeding (seeding of multiple subclones of a branched evolutionary lineage) (1)

Fish plots of the clonal architecture, bar plots of the number of mutations per subclone, and schematics of the phylogeny indicating the relative timing of establishment of each subclone in a cancer with dissemination of three subclones, including two independent subclones resulting from branched evolution (orange and light pink). This cancer had three sampled tumor sites, and all three possible pairwise comparisons among sites are shown. There are two possible routes to dissemination based on the best model fit. Alternative 1 involved dissemination of the pink subclone from the ovary to the omentum, followed by dissemination of the two independent clones between the omentum and ascites in an unknown order and direction. In alternative 2, the pink subclone disseminated from the ovary to both the omentum and the ascites. The cancer was exposed to chemotherapy prior to sampling, and was refractory to the treatment.

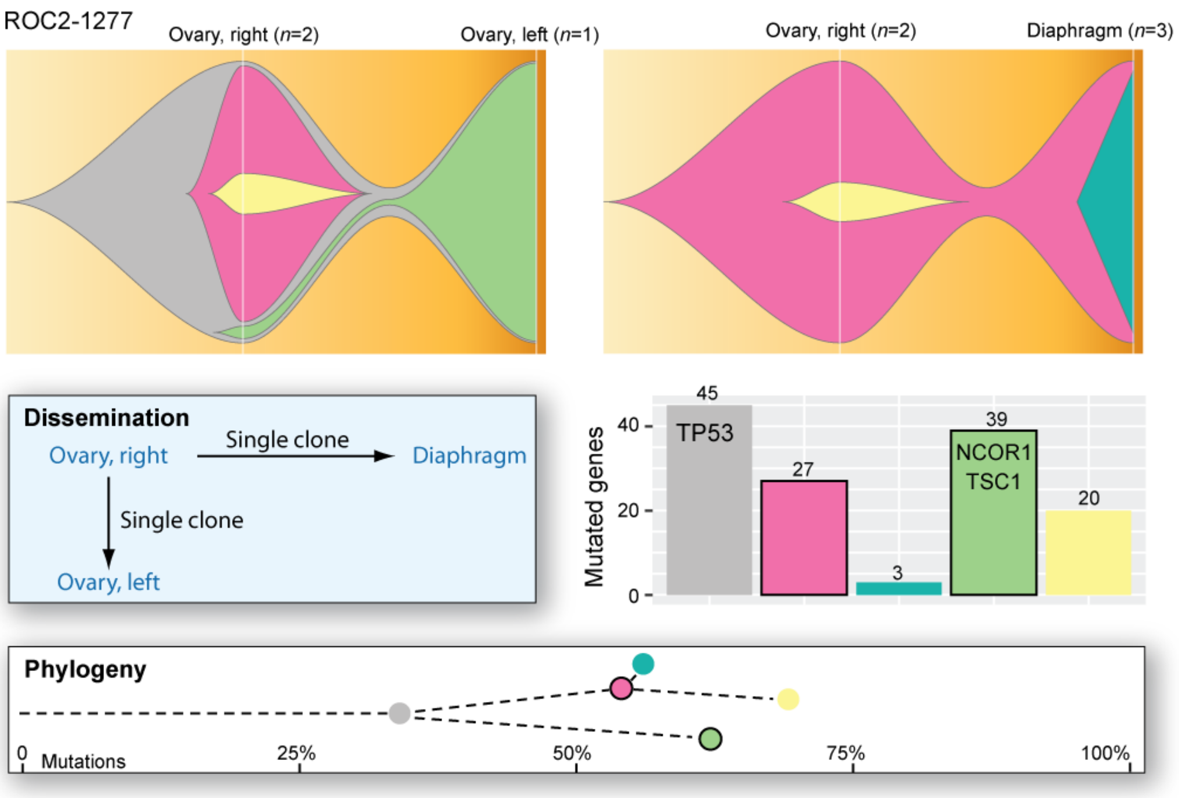

Supplemental Figure S18. Cancer with polyphyletic seeding (seeding of multiple subclones of a branched evolutionary lineage) (2)

Fish plots of the clonal architecture, bar plot of the number of mutations per subclone, and a schematic of the phylogeny indicating the relative timing of establishment of each subclone in a cancer with dissemination of two independent subclones resulting from branched evolution (pink and green). This cancer had three sampled tumor sites, but no dissemination between the left ovarian tumor and the tumor in the diaphragm, and the two possible pairwise comparisons among sites are shown. According to the model, the cancer spread from the right ovary to the left ovary and the diaphragm in two separate steps and by independent subclones.

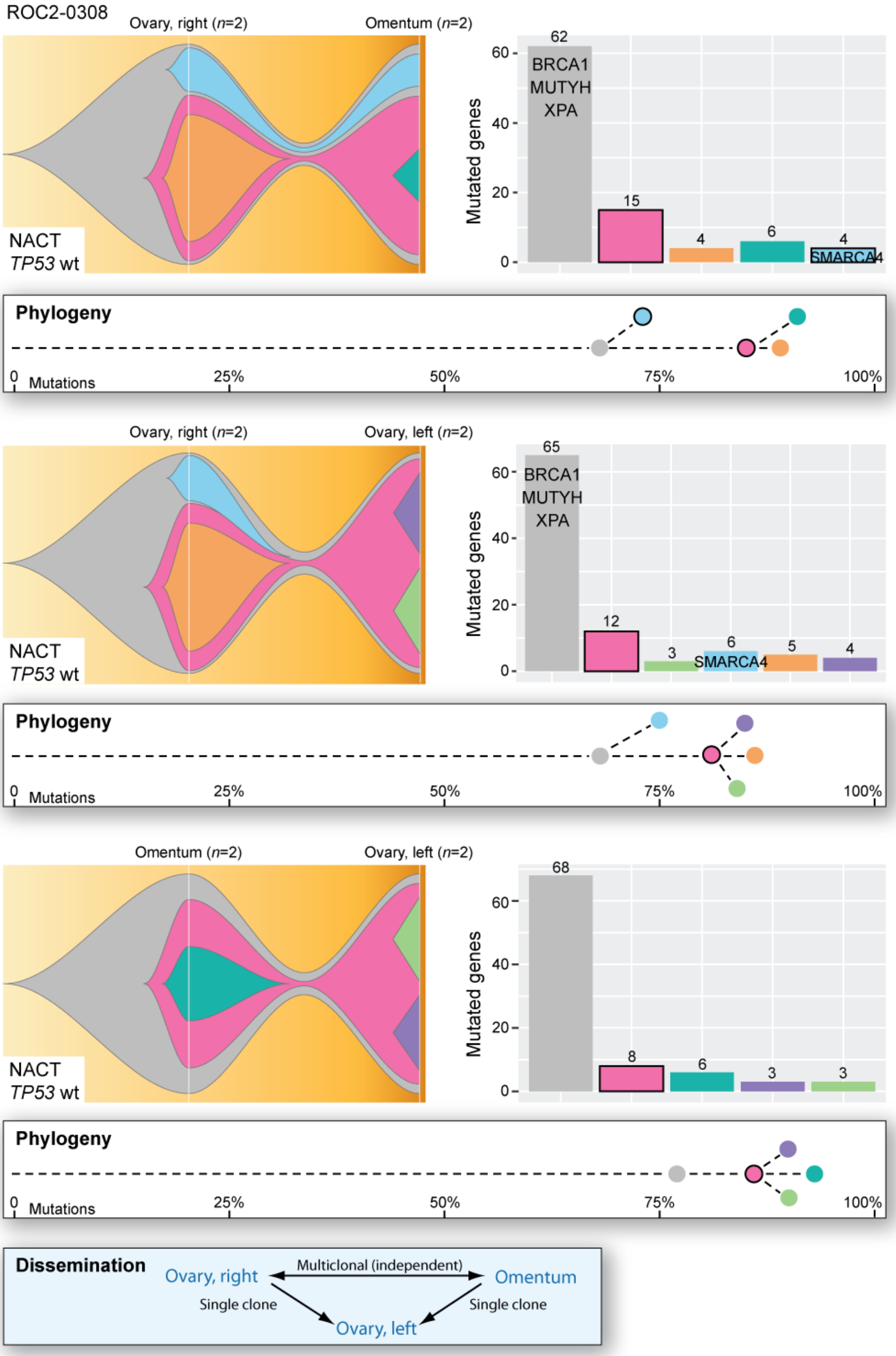
ROC2-0308
Ovary, right (n=2)
Omentum (n=2)
NACT
TP53 wt
Mutated genes
62
BRCA1
MUTYH
XPA
15
4
6
4
SMARCA4
Phylogeny
0 Mutations
25%
50%
75%
100%
Ovary, right (n=2)
Ovary, left (n=2)
NACT
TP53 wt
Mutated genes
65
BRCA1
MUTYH
XPA
12
3
6
SMARCA4
5
4
Phylogeny
0 Mutations
25%
50%
75%
100%
Omentum (n=2)
Ovary, left (n=2)
NACT
TP53 wt
Mutated genes
68
8
6
3
3
Phylogeny
0 Mutations
25%
50%
75%
100%
Dissemination
Ovary, right
Multiclonal (independent)
Omentum
Single clone
Single clone
Ovary, left

Supplemental Figure S19. Cancer with polyphyletic seeding (seeding of multiple subclones of a branched evolutionary lineage) (3)

Fish plots of the clonal architecture, bar plots of the number of mutations per subclone, and schematics of the phylogeny indicating the relative timing of establishment of each subclone in a cancer with dissemination of two independent subclones resulting from branched evolution (blue and pink). This cancer had three sampled tumor sites, and all three possible pairwise comparisons among sites are shown. According to the model, the pink subclone disseminated from the right ovary and/or the omentum to the left ovary, and both the pink and blue subclones disseminated between the right ovary and the omentum in an unknown order and direction. This is the only cancer in the study that was wild-type for *TP53*, and the cancer has a somatic *BRCA1* mutation in the initial clone. The cancer was exposed to chemotherapy prior to sampling.

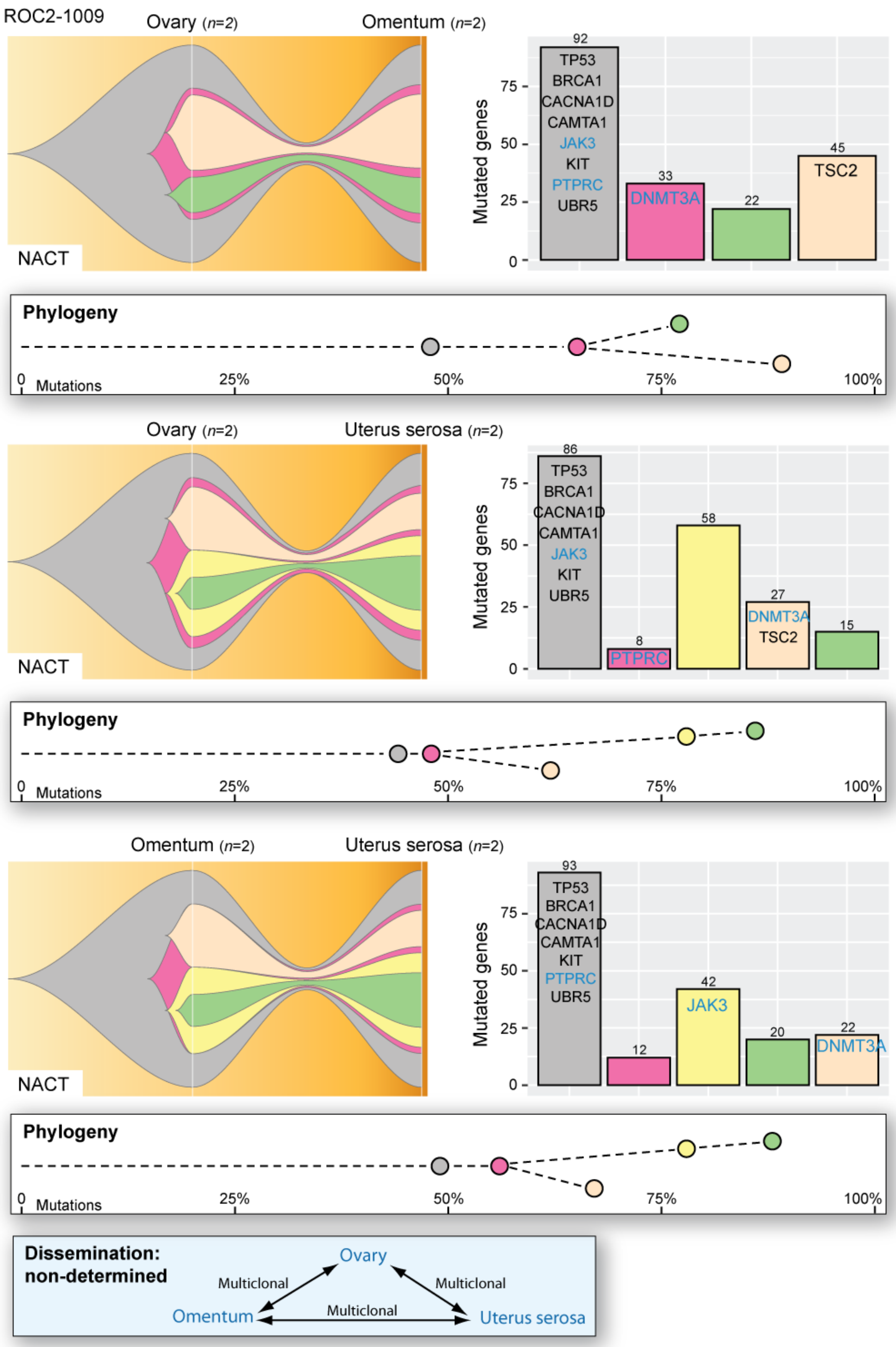
ROC2-1009
Ovary (n=2)
Omentum (n=2)
NACT
Mutated genes
0
25
50
75
92
TP53
BRCA1
CACNA1D
CAMTA1
JAK3
KIT
PTPRC
UBR5
33
DNMT3A
22
45
TSC2
Phylogeny
0
Mutations
25%
50%
75%
100%
Ovary (n=2)
Uterus serosa (n=2)
NACT
86
TP53
BRCA1
CACNA1D
CAMTA1
JAK3
KIT
UBR5
8
PTPRC
58
27
DNMT3A
TSC2
15
Phylogeny
Omentum (n=2)
Uterus serosa (n=2)
NACT
93
TP53
BRCA1
CACNA1D
CAMTA1
KIT
PTPRC
UBR5
12
42
JAK3
20
22
DNMT3A
Phylogeny
Dissemination:
non-determined
Ovary
Multiclonal
Multiclonal
Omentum
Multiclonal
Uterus serosa

Supplemental Figure S20. Cancer with polyphyletic seeding (seeding of multiple subclones of a branched evolutionary lineage) (4)

Fish plots of the clonal architecture, bar plots of the number of mutations per subclone, and schematics of the phylogeny indicating the relative timing of establishment of each subclone in a cancer with dissemination of five subclones, including two independent subclones resulting from branched evolution (beige and yellow). This cancer had three sampled tumor sites, and all three possible pairwise comparisons among sites are shown. It was not possible to determine the direction of seeding among any of the tumor sites. The relatedness of the beige, yellow, and green subclones was uncertain, and the most likely model is illustrated. Genes highlighted in blue changed their allocation between two subclones in the different sample comparisons. The cancer was exposed to chemotherapy prior to sampling.

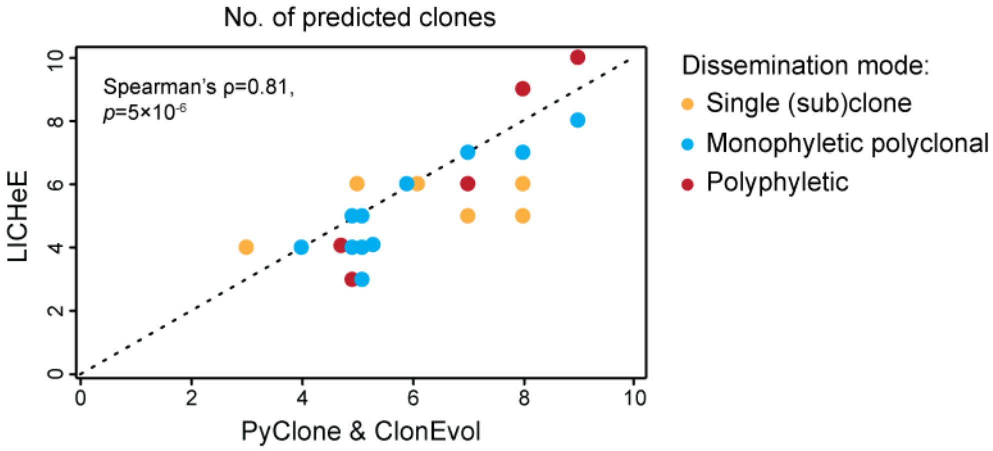

Supplemental Figure S21. Two computational methods for subclone prediction

The scatterplot shows proportionality of the number of subclones predicted by PyClone and ClonEvol relative to LICHeE in each HGSC. Cancers are colored according to the three proposed categories of dissemination. One subclone predicted by PyClone was manually excluded in each of five cancers due to incompatibility with phylogenetic models (failed modeling by ClonEvol). These are illustrated in Supplemental Figures S22 to S24 (ROC2-0812, ROC2-1078, ROC2-1206, ROC2-1801, and ROC2-0190).

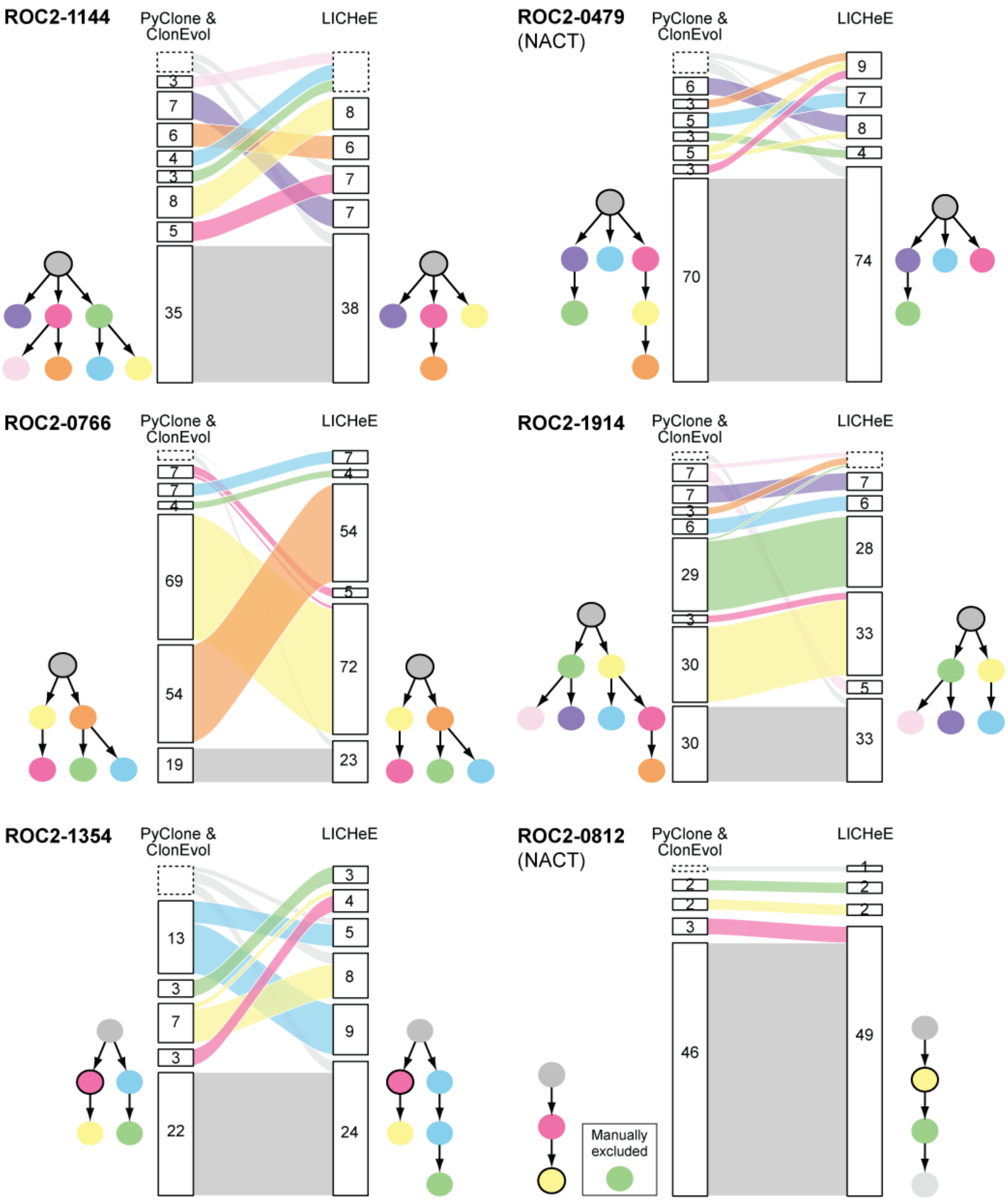

Supplemental Figure S22. Comparison of mutation clusters and phylogenetic inferences by two computational methods in HGSCs with monoclonal dissemination

The alluvial plots show mutation overlaps between mutation clusters/predicted subclones from PyClone and ClonEvol versus LICHeE. The number of mutations is indicated. Dashed outlines mark mutations excluded by each method due to incompatibility with the resulting phylogenetic models. Phylogenetic lineage trees were drawn based on the corresponding fish plots for PyClone and ClonEvol (left), or plotted from the LICHeE output (right). The disseminated clone is marked by a black outline. Both computational methods supported monoclonal dissemination of each cancer, and the inferred relationship of corresponding clones matched.

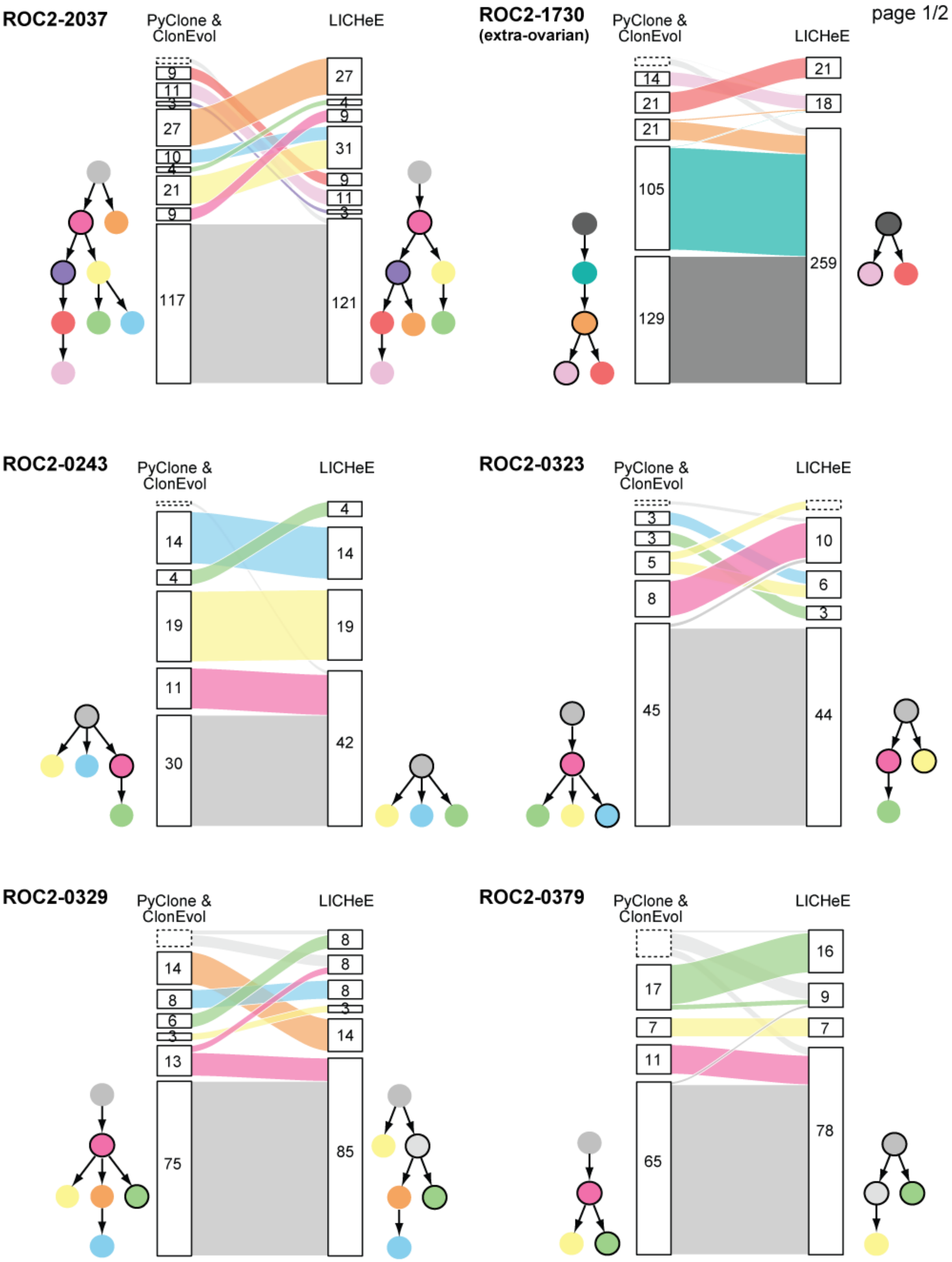
page 1/2
ROC2-2037
PyClone & ClonEvol
LICHeE
ROC2-1730 (extra-ovarian)
PyClone & ClonEvol
LICHeE
ROC2-0243
PyClone & ClonEvol
LICHeE
ROC2-0323
PyClone & ClonEvol
LICHeE
ROC2-0329
PyClone & ClonEvol
LICHeE
ROC2-0379
PyClone & ClonEvol
LICHeE

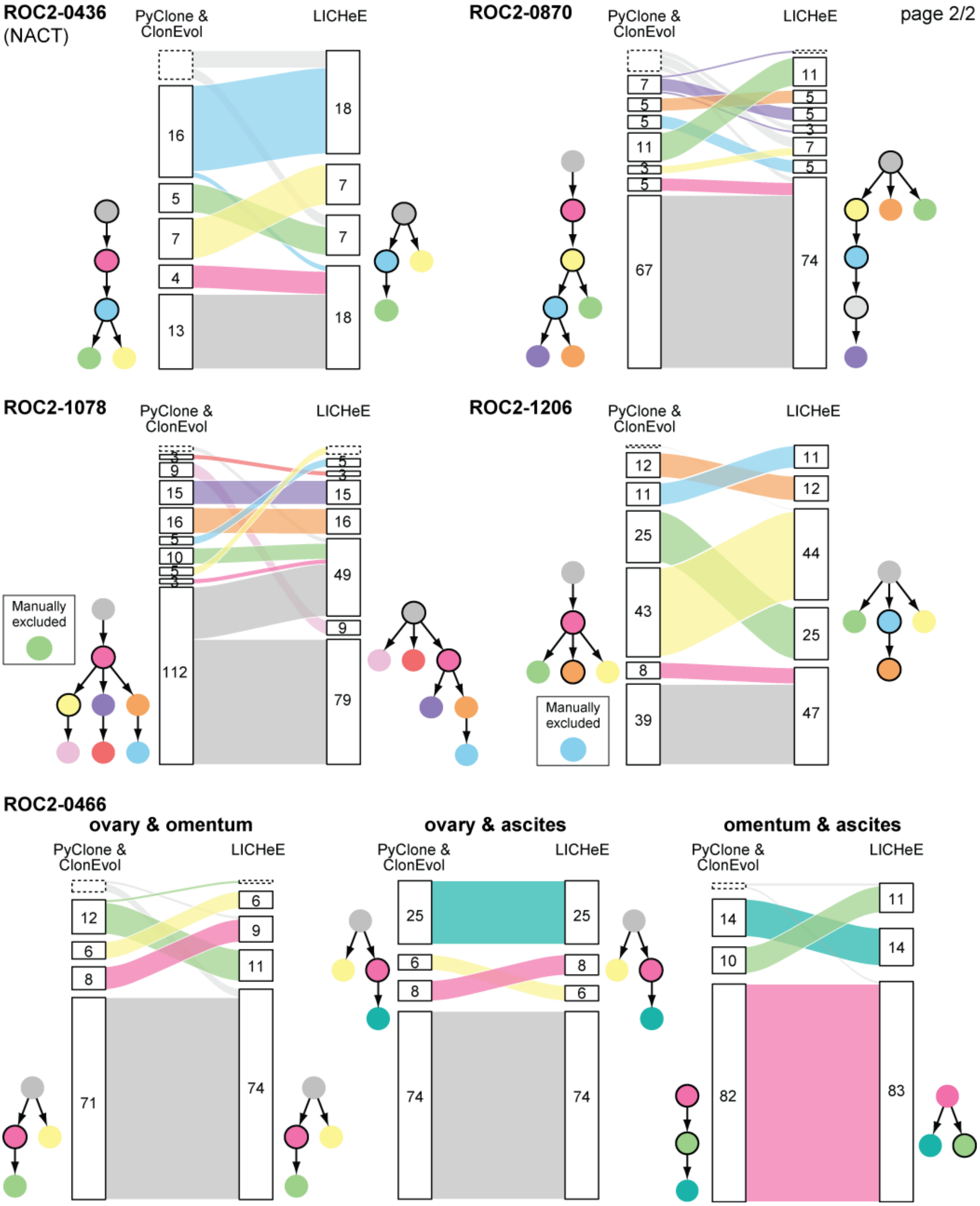

Supplemental Figure S23. Comparison of mutation clusters and phylogenetic inferences by two computational methods in HGSCs with monophyletic polyclonal seeding (seeding of multiple subclones of a linear evolutionary lineage)

The alluvial plots show mutation overlaps between mutation clusters/predicted subclones from PyClone and ClonEvol versus LICHeE. The number of mutations in each subclone is indicated. Dashed outlines mark mutations excluded by each method due to incompatibility with the resulting phylogenetic models. Phylogenetic lineage trees were drawn based on the corresponding fish plots for PyClone and ClonEvol (left), or plotted from the LICHeE output (right). The disseminated clones

are marked by black outlines. Both computational methods supported monophyletic polyclonal seeding of the plotted cancers, with the exception that LICHeE predicted monoclonal dissemination of ROC2-0243 and polyphyletic seeding of ROC2-0323 and ROC2-0379, due to merging and/or a predicted branched evolutionary relationship between two disseminated clones. However, the discordant models predicted by LICHeE were poorly supported by the estimated cellular prevalence of the mutation clusters (Supplemental Figure S12).

ROC2-1801

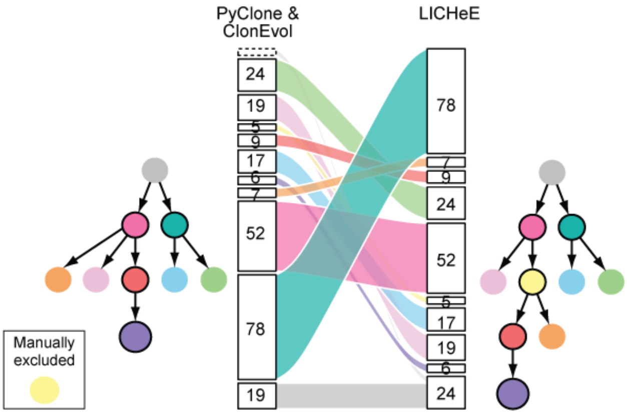

ROC2-0190
(NACT)

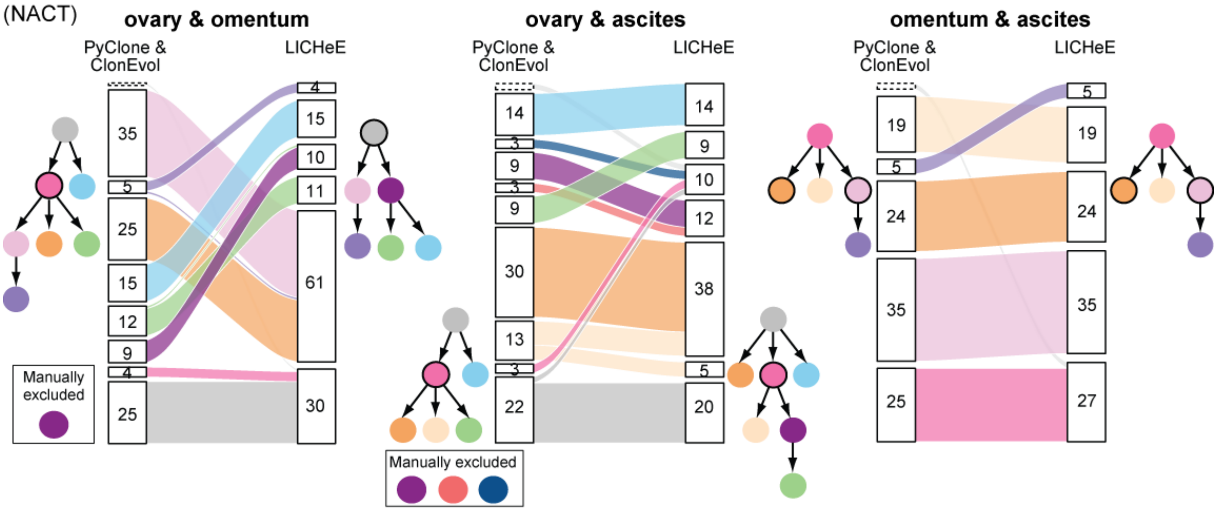

ROC2-1277

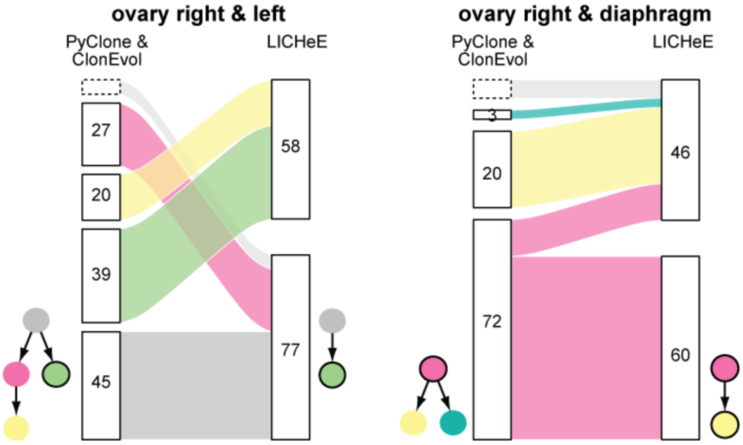

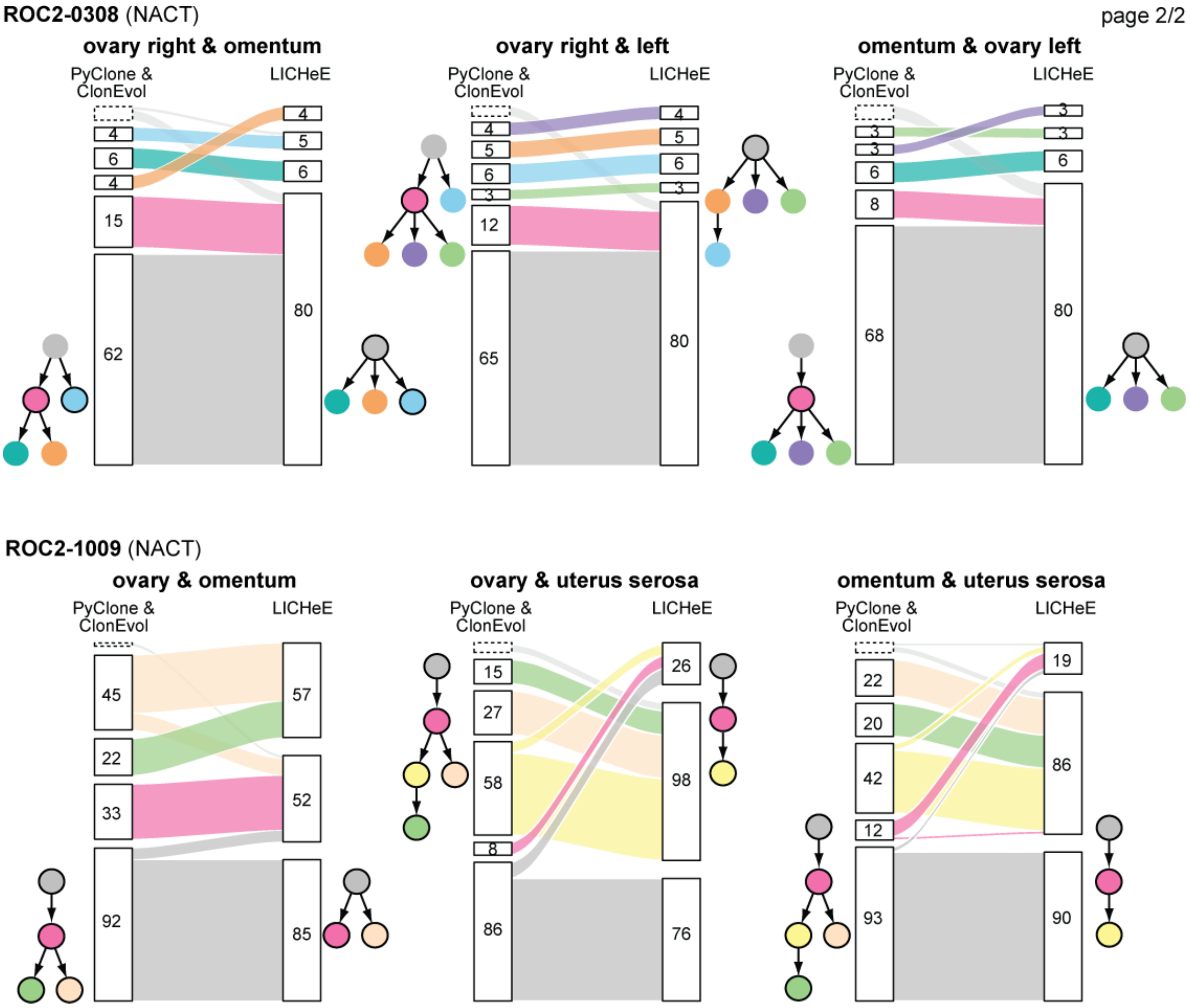

Supplemental Figure S24. Comparison of mutation clusters and phylogenetic inferences by two computational methods in HGSCs with polyphyletic seeding (seeding of multiple subclones of a branched evolutionary lineage)

The alluvial plots show mutation overlaps between mutation clusters/predicted subclones from PyClone and ClonEvol versus LICHeE. The number of mutations in each subclone is indicated. Dashed outlines mark mutations excluded by each method due to incompatibility with the resulting phylogenetic models. Phylogenetic lineage trees were drawn based on the corresponding fish plots for PyClone and ClonEvol (left), or plotted from the LICHeE output (right). The disseminated clones are marked by black outlines. Both computational methods supported polyphyletic seeding of the plotted cancers, with the exception that LICHeE predicted monophyletic polyclonal seeding of ROC2-0308, due to merging of disseminated clones (grey and pink). However, this subclone merger was not supported by the cellular prevalence of the mutation clusters (Supplemental Figure S13).

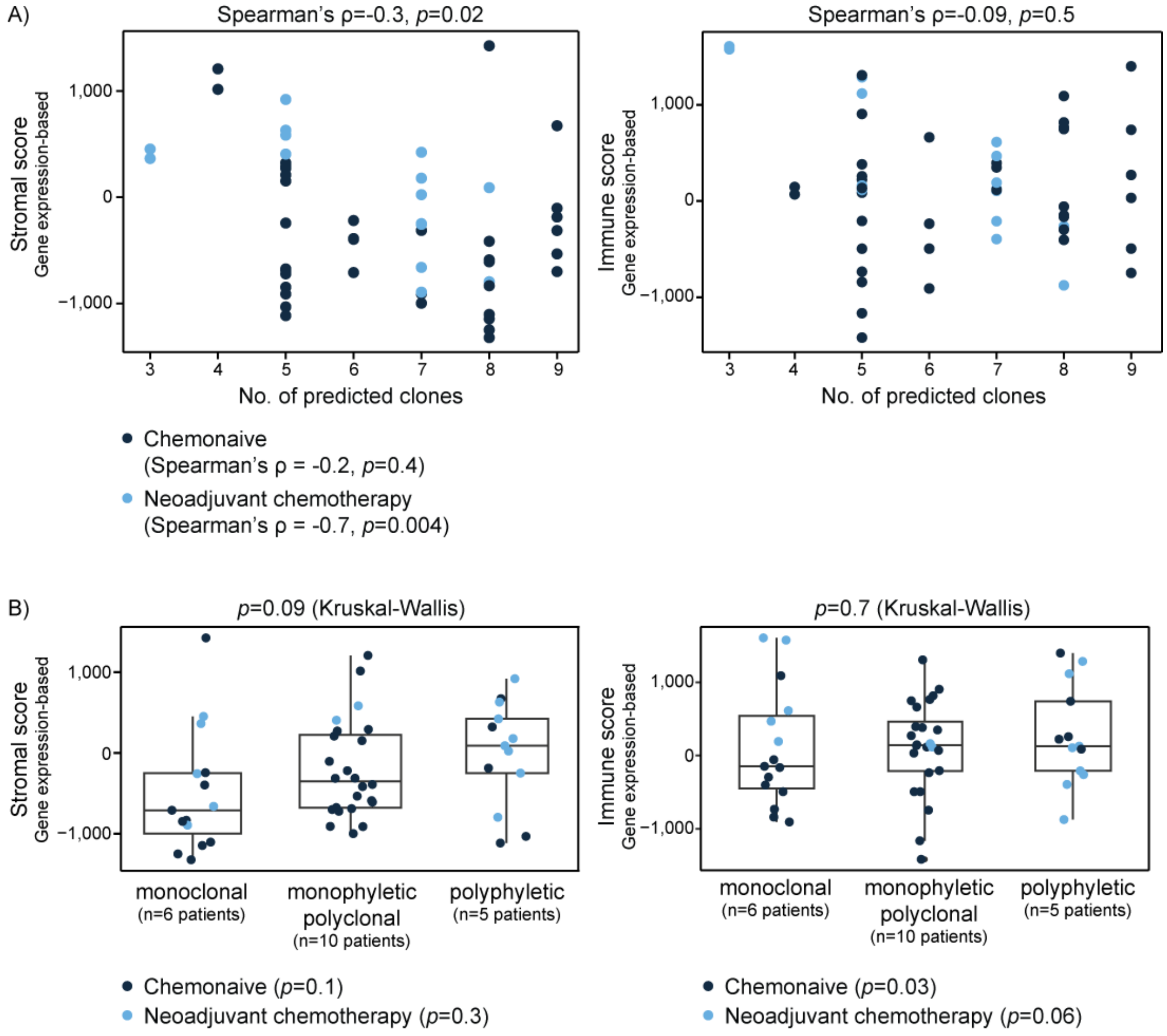

Supplemental Figure S25. Stromal and immune infiltration in ovarian tumors

Stromal and immune scores of ovarian tumors (evaluated by RNA sequencing and ESTIMATE gene expression signatures) according to (A) the number of predicted clones and (B) the dissemination pattern of each HGSC. Only ovarian tumors were included in the analyses, since tumor microenvironment estimates varied according to tumor site (Supplemental Fig. 3; the ovaries were the only tumor site sampled for all patients). The stromal score was weakly negatively correlated to the number of predicted clones per cancer (consistent with results from Lahtinen A et al, *Cancer Cell* 2023;41(6):1103-1117.e1112), but not among chemonaive cancers separately. The stromal score also increased with the complexity of the dissemination pattern, from monoclonal dissemination to polyclonal dissemination of monophyletic and polyphyletic clones, although the difference was not statistically significant (*p*-values from Kruskal-Wallis test). Corresponding analyses of the immune score according to dissemination patterns showed conflicting results in chemonaive and exposed cancers.

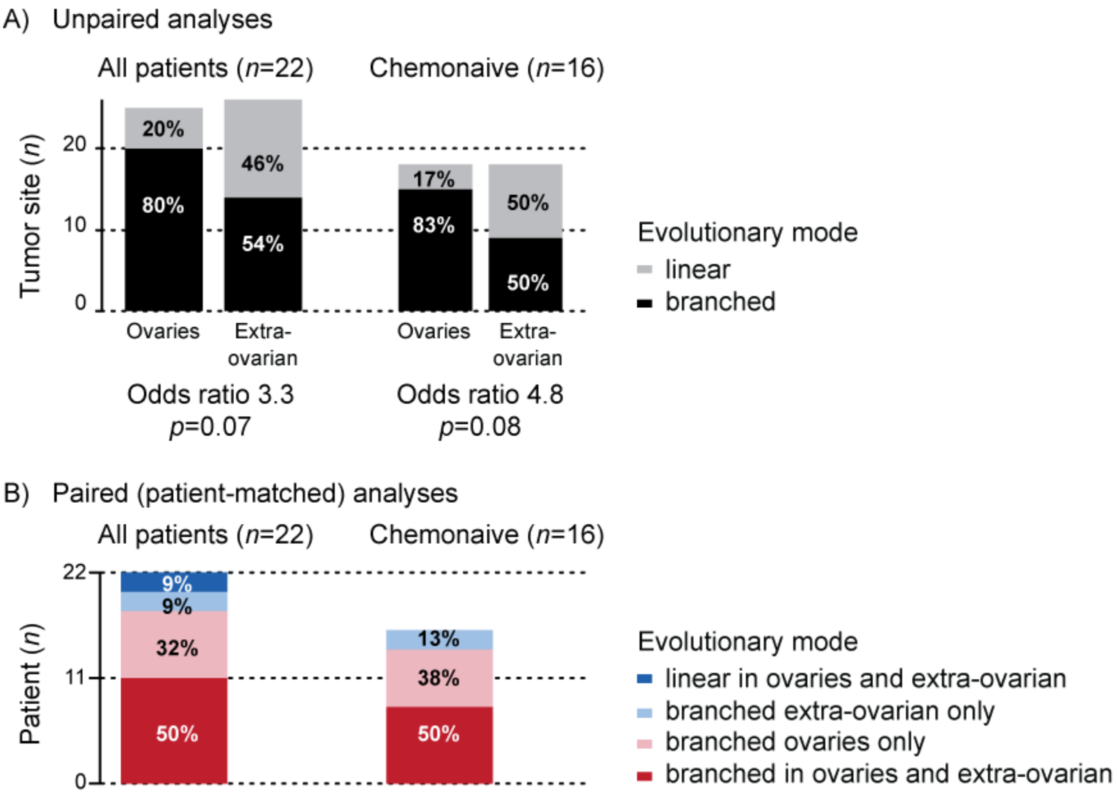

Supplemental Figure S26. More frequent branched evolution in ovarian than extra-ovarian tumors

The evolutionary mode (linear or branched) was summarized per tumor site in each patient, and compared between ovarian and extra-ovarian tumors from all evaluable patients and chemonaive patients in (A) unpaired (not patient-matched) analyses and (B) paired (patient-matched) analyses. There was a higher frequency of branched evolution in ovarian tumors in all analyses, although not significantly different. Odds ratios and *p*-values are from Fisher's exact test.
